# Multi-channel pricing strategies for pharmaceutical supply chains considering channel power and price competition

**DOI:** 10.1371/journal.pone.0322143

**Published:** 2025-05-02

**Authors:** Yan Wen, Yan Wei, Xiyuan Yu

**Affiliations:** School of Business, Qingdao University, Qingdao, China; Universitat Jaume I, SPAIN

## Abstract

In the context of the reality that pharmaceutical manufacturers and retailers are simultaneously opening online sales channels, three different multi-channel pharmaceutical supply chain game models were constructed based on game theory, considering channel power, triple price competition among channels, and health insurance reimbursement policies, among others, comparing and analyzing the effects of each influencing factor on pharmaceutical pricing and profits in the pharmaceutical supply chain. It was found that inter-channel price competition did not always reduce the prices of a retailer’s pharmaceuticals; price competition between physical retail channels and online retail channels could lead to higher pharmaceutical prices, but direct sales channels effectively reduced pharmaceutical prices. Furthermore, as the channel power of pharmaceutical retailers strengthens, retail prices will increase, and retailers’ profits will rise. However, as the channel power of pharmaceutical manufacturers increases, their profits will grow, but retail prices will correspondingly decrease. Appropriate increasing in the health insurance reimbursement rate could improve pharmaceutical pricing in each channel and the total profits of the pharmaceutical supply chains.

## 1 Introduction

The Chinese pharmaceutical market is characterized by distinct features, primarily reflected in the hospital-dominated healthcare system and the strict government regulation of pharmaceutical prices [1]. Additionally, China’s healthcare insurance system provides extensive support to patients, offering subsidies for both hospital visits and pharmaceutical purchases at designated pharmacies through the resident medical insurance program [2]. Although reimbursement rates vary across different hospitals and pharmacies, this subsidy mechanism significantly reduces the financial burden on patients. In contrast, the pharmaceutical markets in countries such as the United States and Germany operate under different models. In the United States, the pharmaceutical market is highly market-driven, with pharmaceutical prices determined by supply and demand, and private health insurance plays a dominant role, leading to higher out-of-pocket expenses for patients [3]. In Germany, the social health insurance system is central, and pharmaceutical prices are negotiated between the government and insurance companies, which helps maintain lower out-of-pocket costs for patients. These international comparisons highlight the unique policy interventions and healthcare coverage features of China’s pharmaceutical market [4].

Additionally, the rapid development of online pharmaceutical commerce has further accelerated the transformation of pharmaceutical sales models within this unique market environment. An increasing number of pharmaceutical manufacturers are adopting a dual-channel sales strategy, selling pharmaceutical s both online and offline, particularly over-the-counter medications. Due to the limitations of physical pharmacies in terms of product variety and service capacity, online pharmaceutical platforms have quickly gained favor among consumers, especially during the COVID-19 pandemic, when their convenience and efficiency were even more apparent [5]. According to iiMedia Research, the pharmaceutical e-commerce market reached a scale of 248.6 billion RMB in 2022, representing a 10.0% increase from 226.0 billion RMB in 2021. It is projected that the market size will exceed 340.0 billion RMB by 2026. On the one hand, large pharmaceutical manufacturers such as Baiyunshan and Hapharm Group have ventured into e-commerce, opening direct sales channels on platforms such as Jingdong and Tmall to increase profitability and enhance corporate influence, while on the other hand, large pharmacy chains such as Nepstar and Shuyu Civilian Pharmacy have opened online retail channels on platforms such as Ele.me, Meituan, and others, while continuously expanding the scale of their offline stores [6,7]. The multi-channel distribution of pharmaceuticals increases the flexibility of the pharmaceutical supply chain and intensifies inter-channel price competition.

The widespread adoption of this multi-channel sales model has further highlighted the differences in channel power among various entities within the supply chain and their impact on market competition. In the vertical structure formed by pharmaceutical manufacturers and retailers, channel relationships exhibit both similarities and significant differences [8]. For example, when the vertical channel power of upstream and downstream firms is considered, this vertical structure exhibits a different channel power structure. In transactions between pharmaceutical manufacturers such as Pfizer, Novartis, and GlaxoSmithKline and ordinary pharmacies or hospitals, the pharmaceutical manufacturers dominate with position and stronger channel power compared with retailers, resulting in a “strong-weak” channel power structure. In contrast, in the case of Yunnan Baiyao and Tasly’s transactions with Guoda Pharmacy and LBX Pharmacy, both manufacturers and retailers have strong positions and channel power in the supply chain, demonstrating a “strong-strong” channel power structure. While in transactions between small pharmaceutical manufacturers and large retailers such as Yifeng Pharmacy and Yixintang Pharmaceutical, the small manufacturers have a weak market position and little say in the market, and thus display a “weak-strong” channel power structure in the sale of their pharmaceuticals. It can be seen that the same vertical structure of the supply chain undergoes significant changes when channel power is taken into account, resulting in different vertical channel power structures [8]. Therefore, when faced with different channel forces, how to coordinate the price competition between channels becomes particularly important.

Based on the above analysis, this paper aims to explore the impact of different channel forces and inter-channel price competition in the context of pharmaceutical e-commerce. The paper will address the following three questions:

1. How do different channel forces affect the profitability of pharmaceutical supply chain members and the pricing of pharmaceuticals across channels differently?
2. Is inter-channel price competition always conducive to lowering retail pharmaceutical prices?
3. What are the impacts of two different online channel models, namely, the manufacturer’s direct channel and the retailer’s online channel, on the profitability and overall performance of various members of the pharmaceutical supply chain?

To answer the above questions, we considered a multi-channel pharmaceutical supply chain consisting of over the counter (OTC) pharmaceutical manufacturers and a single pharmaceutical retailer (a health insurance designated retail pharmacy). Three different multichannel pharmaceutical supply chain game models were also constructed by combining channel power, triple price competition among channels, and health insurance reimbursement policies, and compared the effects of the intensity of price competition and changes in the health insurance reimbursement ratio as well as the opening of online sales channels and changes in competitive power, on the pricing of pharmaceuticals and the profit in the pharmaceutical supply chain. It is hoped that this analysis will provide a reference for the long-term development of pharmaceutical supply chains and the choice of multi-channel strategies.

The contribution of this paper is twofold. First, in terms of theoretical research, it enriches research on the multichannel sales of pharmaceuticals by pharmaceutical manufacturers and retailers, and extends research into the pricing of multichannel pharmaceutical supply chains. Second, its practical significance lies in the potential for enhancing the efficiency of the entire pharmaceutical supply chain by assessing the impact of price competition and forces in different channels on pharmaceutical pricing and the profits of each member of the pharmaceutical supply chain, as well as providing a decision-making basis for the formulation of pharmaceutical prices, effectively promoting the healthy and stable development of the pharmaceutical industry.

The rest of the paper is organized as follows. We review the relevant literature in Sect. 2 and describe the problem description and basic assumptions in Sect. 3. Section 4 constructs the model and derives the equilibrium solution. Section 5 validates the previous conclusions by numerically analyzing the key parameters and section 6 summarizes the conclusions drawn in the paper and provides managerial insights.

## 2 Literature review

In this section, the relevant literature will be reviewed from three aspects: pharmaceutical supply chain, online and offline multichannel models, and channel forces.

### 2.1 Pharmaceutical supply chain

The pharmaceutical supply chain is a functional network around a core enterprise [9], mainly involving raw material supply enterprises, pharmaceutical enterprises, terminal pharmaceutical sales organizations, patients and other links [10,11]. Currently, there are many scholars who have conducted in-depth research on the operation and management of the pharmaceutical supply chain around the centralized procurement of pharmaceuticals [12,13], pharmaceutical quality [14], pharmaceutical prices [15], pharmaceutical market demand [16], and the selection of innovative and generic pharmaceuticals [17,18]. One of the most relevant to this paper is the study of pharmaceutical market demand and pharmaceutical prices. Zhou et al. [19] analyzes the impact of the zero-price-added pharmaceutical policy on the level of medical services, the price of medical services, and the price of pharmaceuticals by constructing a game model of the pharmaceutical supply chain composed of pharmaceutical suppliers and public hospitals; Iacocca and Mahar [20] discusses the impact of the value of partnership between mail order and pharmacy chains on pharmaceutical pricing by developing a mathematical model; Wu et al. [21] explores the impact of patient sensitivity to pharmaceutical prices and demand uncertainty on pricing and production decisions for pharmaceuticals; Yang et al. [8] discusses optimal pricing, optimal performance and optimal social welfare of pharmaceuticals under price cap regulation in different power structures; Son [22] explores the relationship between generic pharmaceutical prices and market competitiveness in Korea and finds rare price competition among a large number of generic pharmaceutical manufacturers.

Although more literature has analyzed the impact of factors such as pharmaceutical price regulation and demand uncertainty on pharmaceutical pricing, little literature has considered the impact of different channel power and the intensity of cross-price competition among different channels on pharmaceutical pricing. Therefore, this paper will explore the pricing strategy and performance of multichannel pharmaceutical supply chains by considering the variation in the intensity of cross-price competition among multiple channels and combining factors such as channel power and health insurance regulation.

### 2.2 Online and offline multi-channel model

The choice of sales channel model is an important research area in e-tailing [23]. At present, many scholars conduct in-depth research on the selection of sales channel mode for different products and industries, mainly focusing on electronic products [24], fresh products [25,26], financial industry [27], shipping industry [28] and other fields.

At the same time, with the deepening research on sales channel models, the issue of multi-channel adoption in the pharmaceutical industry has gradually become a focal point of academic discussion. For example, Wang and Yang [7], who consider the impact of consumer preferences on manufacturers’ behavior in opening online channels. The results suggest that an increase in consumer preference for online channels increases the demand, price, and manufacturer profitability of the pharmaceutical online channel, and that manufacturers are more inclined to open online channels. Lan et al. [29] examined the impact of healthcare reimbursement systems and patient preferences on manufacturers’ online channel incursions and further analyzed the impact of perceived quality of pharmaceuticals on social welfare. Meanwhile Zheng et al. [6] argues that the impact of price limit policies on channel selection of pharmaceuticals cannot be ignored, and for this reason, it considers a two-stage supply chain consisting of pharmaceutical manufacturers and pharmacies, and investigates the opening of manufacturers’ online channels under the price cap model. Karray and Sigué [30] focuses on whether traditional retailers add an online channel to their offline channel sales. Consider one of the retailers leading the launch of the online channel, with the other retailer following suit. The second is that both offline retailers simultaneously consider whether to develop an online presence. Yang et al. [8] Explore optimal pricing, optimal performance and optimal social welfare under different power structures under the premise that pharmaceutical retailers’ pharmacies open O2O channels.

Unfortunately, most of the above literature focuses on the opening of online channels by pharmaceutical manufacturers, and less on the opening of online sales channels by retailers. Therefore, this paper takes into account the situation where pharmaceutical manufacturers and retailers open online and offline sales channels at the same time.

### 2.3 Channel power

In the process of marketing, channel power is primarily the influence, control, and relative profitability of individual members of a distribution channel (e.g., retailers, wholesalers, etc.) over other members. Each member of the supply chain aspires to pursue its own profitability through a powerful position. Therefore, different power structures determine different decision sequences of supply chain members [31].

The channel power relationships between pharmaceutical manufacturers and retailers in the pharmaceutical supply chain are similarly distinctly different. The studies in the existing literature can be broadly classified into two categories: first, studies on pharmaceutical supply chains where pharmaceutical manufacturers are dominant and have decision-making dominance. For example, Chen et al. [32] comparatively analyze the impact of different price control policies on pharmaceutical pricing strategies in manufacturer-dominated pharmaceutical supply chains; Huang and Wu [33] compared and analyzed the cooperative decision-making model and the decentralized decision-making model by taking a dual-channel supply chain dominated by a pharmaceutical manufacturer as the object of study and considering the effects of the quality of the pharmaceuticals and the sales efforts of the pharmaceutical retailers on the demand. The results show that the overall profit of the supply chain system under cooperative decision-making is always higher than the overall profit under decentralized decision-making. Secondly, with the emergence of large retail platforms such as Jingdong and Tmall or large pharmacy chains such as Nepstar and Shuyu Civilian Pharmacy, prompting a shift in the channel power of the pharmaceutical supply chain to retailers, the focus of some scholars’ research has gradually shifted to the pharmaceutical supply chain where pharmaceutical retailers are the decision-makers. For example, Zheng et al. [6] studied the impact of price controls on pharmaceutical pricing and profitability in a pharmacy-dominated pharmaceutical supply chain; Yang et al. [8] studied pharmaceutical supply chains with pharmacies as Stackelberg leaders to explore optimal pricing, optimal performance, and optimal social welfare under this power structure in order to understand the impact of price controls and power structures on pharmaceutical supply chains.

However, most of the existing literature studies the impact of different power structures on the decision-making and profitability of each member under a fixed channel model, and few studies have addressed the pharmaceutical pricing problem of multichannel pharmaceutical supply chains under different channel powers, while the research in this paper bridges this gap.

Existing research primarily focuses on pharmaceutical supply chain pricing within traditional channel models, lacking a comprehensive analysis of multi-channel sales models. Unlike previous studies, this paper simultaneously considers the scenarios where both pharmaceutical manufacturers and retailers operate online and offline channels. Based on this, we analyze the impact of factors such as channel power, price competition intensity, and consumer out-of-pocket costs on the profits of supply chain members and overall performance. This multidimensional perspective provides deeper theoretical support and practical guidance for pharmaceutical supply chain pricing. To further compare this study with existing studies, the studies related to this study are listed in Table 1.

**Table 1:** Comparison among related studies.

Paper	Pricing strategy	Manufacturer-led	Retailer-led	Multichannel
Zheng et al. [6]	No	No	Yes	No
Yang et al. [8]	Yes	No	Yes	No
Iacocca and Mahar [20]	Yes	No	Yes	No
Wu et al. [21]; Son [22]	Yes	Yes	No	No
Wang and Yang [7]; Jayachandran [17]; Huang and Wu [33]	No	Yes	No	No
This paper	Yes	Yes	Yes	Yes

## 3 Problem description and modeling

### 3.1 Description of the problem

**Fig 1:**
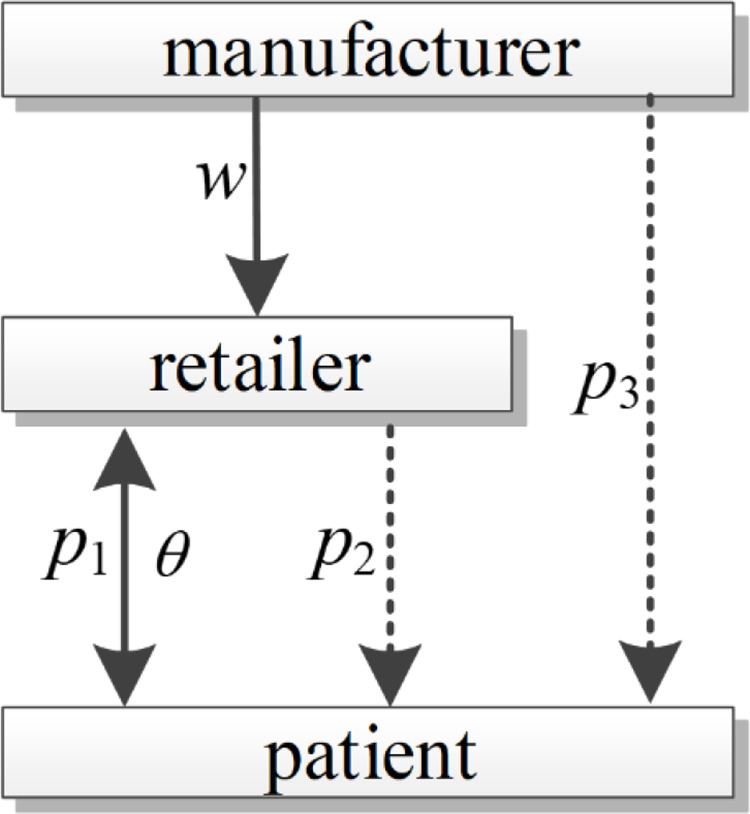
Multi-channel pharmaceutical supply chain model architecture.

This paper constructs a pharmaceutical supply chain system consisting of a single OTC pharmaceutical manufacturer and a single pharmaceutical retailer (health insurance designated retail pharmacy). The pharmaceutical retailer purchases the pharmaceuticals from the pharmaceutical manufacturer at wholesale price *w* and sells the pharmaceuticals to the patients through offline physical channels as well as online channels such as Ele.me, Meituan and others at retail price $p_{1}$ and $p_{2}$ respectively. In addition, pharmaceutical manufacturers have opened an additional direct sales channel on platforms such as Jingdong and Tmall to sell pharmaceuticals directly to patients at the price of $p_{3}$. The model architecture is described in Fig 1. The symbols and meanings of key parameters and decision variables are shown in Table 2.

**Table 2:** Symbols and meanings.

Symbols	Meanings
$p_{\text{1}}\left(t\right)$	Retail prices of pharmaceuticals in offline pharmacies
$p_{2}\left(t\right)$	Online pharmacy pharmaceutical retail prices
$p_{3}\left(t\right)$	Retail price of pharmaceuticals sold directly from the platform
*w*	Wholesale prices for pharmaceutical retailers
*a*	Market size
*θ*	Consumers’ out-of-pocket percentage for offline channel purchases
*λ*	Commission rates charged by the platform
*ϕ*	Internet pharmaceutical market share
*Φ*	Online pharmacy market as a percentage of the online pharmaceutical market
*β*	Proportion of market size increase and expansion after the opening of two online sales channels
$k_{i}(i=1,2,3)$	Price elasticity of demand coefficients for physical retail channels, online retail channels, and direct sales channels
$\gamma_{1}$	Cross-price elasticity between physical and online retail channels
$\gamma_{2}$	Cross-price elasticity between the brick-and-mortar retail channel and the manufacturer’s direct sales channel
$\gamma_{3}$	Cross-price elasticity between the online retail channel and the manufacturer’s direct sales channel

### 3.2 Model assumptions

**Assumption 1** Based on the relevant regulations of China’s health insurance system, the policy that patients can enjoy health insurance reimbursement benefits when purchasing pharmaceuticals through online sales channels is still in the pilot process, so this paper assumes that patients can enjoy health insurance reimbursement benefits when purchasing pharmaceuticals through physical channels only, and draws on Wen and Liu’s [34] setting of health insurance reimbursement ratio, assuming that the patient’s out-of-pocket payment ratio is θ∈(0,1), then the corresponding health insurance reimbursement ratio is 1−θ.

**Assumption 2** When a manufacturer or retailer sells pharmaceuticals in an online sales channel, both are required to pay a certain percentage of platform commissions based on sales [35,36], assuming that the commissions of different platforms are the same as a percentage of sales, and both are λ∈(0,1).

**Assumption 3** Manufacturers incur unit production costs of c, similar to Yan [37] we normalize unit production costs to zero, which does not affect our main results.

To summarize, drawing on the modeling of pharmaceutical demand and price competition in the literature [38,39], it is assumed that the demand functions for pharmaceuticals in the brick-and-mortar channel of a pharmaceutical retailer, the online channel, and the direct sales channel of a manufacturer are as follows


$$\begin{array}{l} Q_{1}=(1-\phi )a-\theta (k_{\text{1}}+\gamma_{1}+\gamma_{2})p_{1}+\gamma_{1}p_{2}+\gamma_{2}p_{3}\\ Q_{2}=\Phi (\phi +\beta )a-(k_{\text{2}}+\gamma_{1}+\gamma_{3})p_{2}+\theta \gamma_{1}p_{1}+\gamma_{3}p_{3}\\ Q_{\text{3}}=(1-\Phi )(\phi +\beta )a-(k_{3}+\gamma_{2}+\gamma_{3})p_{3}+\theta \gamma_{2}p_{1}+\gamma_{3}p_{2} \end{array}$$ (1)

Where *a* represents the overall size of the market. ϕ∈(0,1) represents the proportion of the online sales channel’s diversion from the physical channel’s market size. Φ∈(0,1) represents the share of online channels in the total market size of online sales channels. Whether manufacturers or retailers, the addition of online sales channels will increase brand awareness and generate additional sales for pharmaceuticals, denote *β* as the proportion of the market size increased by the opening of the two online sales channels. Assuming that $k_{1}$, $k_{2}$, and $k_{3}$ are the price elasticity of demand coefficients for the brick-and-mortar channel, the online channel, and the direct sales channel, respectively. These coefficients satisfy $1>k_{2}>k_{1}>0$ and $1>k_{3}>k_{1}>0$ since patients have higher price sensitivity to pharmaceuticals sold through the two online distribution channels [29]. $\gamma_{1}$ denotes the cross-price elasticity between the physical channel and the online channel, $\gamma_{2}$ denotes the cross-price elasticity between the physical channel and the manufacturer’s direct sales channel, and $\gamma_{3}$ denotes the cross-price elasticity between the online channel and the manufacturer’s direct sales channel, i.e., $\gamma_{1}$, $\gamma_{2}$, and $\gamma_{3}$ are measures of the degree of price competition among different channels, respectively.

To simplify the formula, denote the potential market sizes of the retailer’s physical channel, the online channel, and the manufacturer’s direct sales channel by $a_{1}$, $a_{2}$, and $a_{3}$, i.e., $a_{1}=(1-\phi )a$, $a_{2}=\Phi (\phi +\beta )a$, and $a_{3}=(1-\Phi )(\phi +\beta )a$, respectively, and such that $f_{1}=k_{\text{1}}+\gamma_{1}+\gamma_{2}$, $f_{2}=k_{\text{2}}+\gamma_{1}+\gamma_{3}$, and $f_{3}=k_{3}+\gamma_{2}+\gamma_{3}$, the demand function can be further expressed as


$$\begin{array}{l} Q_{1}=a_{1}-\theta f_{1}p_{1}+\gamma_{1}p_{2}+\gamma_{2}p_{3}\\ Q_{2}=a_{2}-f_{2}p_{2}+\theta \gamma_{1}p_{1}+\gamma_{3}p_{3}\\ Q_{3}=a_{3}-f_{3}p_{3}+\theta \gamma_{2}p_{1}+\gamma_{3}p_{2} \end{array}$$ (2)

This leads to a profit function for the pharmaceutical retailer and the pharmaceutical manufacturer respectively as


$$\begin{array}{l} \pi_{\text{R}}=(p_{1}-w)Q_{1}+(1-\lambda )(p_{2}-w)Q_{2}\\ \pi_{\text{M}}=(1-\lambda )p_{3}Q_{3}+w(Q_{1}+Q_{2}) \end{array}$$ (3)

## 4 Model analysis

### 4.1 Nash game model with equal channel power (Model N)

In Model N, the pharmaceutical manufacturer and the pharmaceutical retailer have equal channel power, each with its own profit maximization as its business objective, and each will make decisions simultaneously, rationally and independently during the planning period. For example, in the transactions between well-known domestic pharmaceutical manufacturers such as Hengrui and China Shijiazhuang Pharmaceutical and large pharmacy chains such as Sinopharm and Nepstar, pharmaceutical manufacturers and pharmacy chains are in a stronger position in the pharmaceutical supply chain, with comparable channel power and no clear dominant relationship. The objective functions of the pharmaceutical retailer and the pharmaceutical manufacturer are


$$\begin{array}{l} \underset{p_{1},p_{2}}{\max}V_{\text{R}}^{N}=\left(p_{1}-w\right)\left(a_{1}-\theta f_{1}p_{1}+\gamma_{1}p_{2}+\gamma_{2}p_{3}\right)+\left(1-\lambda \right)\left(p_{2}-w\right)\left(a_{2}-f_{2}p_{2}+\theta \gamma_{1}p_{1}+\gamma_{3}p_{3}\right)\\ \underset{p_{3}}{\max}V_{\text{M}}^{N}=p_{3}\left(1-\lambda \right)\left(a_{3}-f_{3}p_{3}+\theta \gamma_{2}p_{1}+\gamma_{3}p_{2}\right)+w\left(a_{1}-\theta f_{1}p_{1}+\gamma_{1}p_{2}+\gamma_{2}p_{3}+a_{2}-f_{2}p_{2}+\theta \gamma_{1}p_{1}+\gamma_{3}p_{3}\right) \end{array}$$ (4)

**Proposition 1** Under the model N scenario, the online and offline retail prices of pharmaceutical retailers are


$$\begin{array}{l} p_{1}^{N*}=A_{1}+B_{1}\frac{\left(1-\lambda \right)\left(a_{3}+\theta \gamma_{2}A_{1}+\gamma_{3}C_{1}\right)+w\left(\gamma_{2}+\gamma_{3}\right)}{\left(1-\lambda \right)\left(2f_{3}-\theta \gamma_{2}B_{1}-\gamma_{3}D_{1}\right)}\\ p_{2}^{N*}=C_{1}+D_{1}\frac{\left(1-\lambda \right)\left(a_{3}+\theta \gamma_{2}A_{1}+\gamma_{3}C_{1}\right)+w\left(\gamma_{2}+\gamma_{3}\right)}{\left(1-\lambda \right)\left(2f_{3}-\theta \gamma_{2}B_{1}-\gamma_{3}D_{1}\right)} \end{array}$$ (5)

Pharmaceutical manufacturer direct channel sale prices is


$$p_{3}^{N*}=\frac{\left(1-\lambda \right)\left(a_{3}+\theta \gamma_{2}A_{1}+\gamma_{3}C_{1}\right)+w\left(\gamma_{2}+\gamma_{3}\right)}{\left(1-\lambda \right)\left(2f_{3}-\theta \gamma_{2}B_{1}-\gamma_{3}D_{1}\right)}$$ (6)

where


$$\{\begin{array}{l} {\text{A}}_{1}=\frac{2f_{2}\left(1-\lambda \right)\left[a_{1}-\theta \gamma_{1}\left(1-\lambda \right)w+\theta f_{1}w\right]+\gamma_{1}\left[1+\theta \left(1-\lambda \right)\right]\left[a_{2}\left(1-\lambda \right)-\gamma_{1}w+f_{2}\left(1-\lambda \right)w\right]}{4\theta f_{1}f_{2}\left(1-\lambda \right)-\gamma_{1}^{2}{\left[1+\theta \left(1-\lambda \right)\right]}^{2}}\\ {\text{B}}_{1}=\frac{\left(1-\lambda \right)\left[1+\theta \left(1-\lambda \right)\right]\gamma_{1}\gamma_{3}+2\gamma_{2}f_{2}\left(1-\lambda \right)}{4\theta f_{1}f_{2}\left(1-\lambda \right)-\gamma_{1}^{2}{\left[1+\theta \left(1-\lambda \right)\right]}^{2}}\\ {\text{C}}_{1}=\frac{a_{2}\left(1-\lambda \right)-\gamma_{1}w+f_{2}\left(1-\lambda \right)w+\gamma_{1}\left[1+\theta \left(1-\lambda \right)\right]{\text{A}}_{1}}{2f_{2}\left(1-\lambda \right)}\\ {\text{D}}_{1}=\frac{\gamma_{1}\left[1+\theta \left(1-\lambda \right)\right]{\text{B}}_{1}+\gamma_{3}\left(1-\lambda \right)}{2f_{2}\left(1-\lambda \right)} \end{array}$$ (7)

Further, bringing $p_{1}^{N*}$, $p_{2}^{N*}$, and $p_{3}^{N*}$ back into the equation yields the optimal profits $\pi_{\text{R}}^{\text{N}*}$ and $\pi_{\text{M}}^{\text{N}*}$ for the pharmaceutical retailer and pharmaceutical manufacturer, which are not shown here due to the complexity of the equation.

### 4.2 Retailer-led Stackelberg game model (Model RS)

In model RS, the pharmaceutical retailer with stronger channel power has the dominant decision-making power as the dominant player in the pharmaceutical supply chain. For example, in the process of cooperation between a large pharmaceutical retailer, GuoDa Pharmaceutical store, and an unknown pharmaceutical manufacturer, the smaller manufacturer has a weaker market position relative to GuoDa Drugstore and has almost no channel power. Under this model, the pharmaceutical retailer first determines the retail price of the pharmaceutical for both online and offline physical channels, followed by the pharmaceutical manufacturer determining the wholesale price of the pharmaceutical and the retail price of the pharmaceutical for the online direct sales channel. The objective functions of the pharmaceutical manufacturer and the pharmaceutical retailer are


$$\begin{array}{l} \underset{p_{1},p_{2}}{\max}V_{\text{R}}^{\text{RS}}=\left(p_{1}-w\right)\left(a_{1}-\theta f_{1}p_{1}+\gamma_{1}p_{2}+\gamma_{2}p_{3}\right)+\left(1-\lambda \right)\left(p_{2}-w\right)\left(a_{2}-f_{2}p_{2}+\theta \gamma_{1}p_{1}+\gamma_{3}p_{3}\right)\\ \underset{p_{3}}{\max}V_{\text{M}}^{\text{RS}}=p_{3}\left(1-\lambda \right)\left(a_{3}-f_{3}p_{3}+\theta \gamma_{2}p_{1}+\gamma_{3}p_{2}\right)+w\left(a_{1}-\theta f_{1}p_{1}+\gamma_{1}p_{2}+\gamma_{2}p_{3}+a_{2}-f_{2}p_{2}+\theta \gamma_{1}p_{1}+\gamma_{3}p_{3}\right) \end{array}$$ (8)

**Proposition 2** In the model RS scenario, the online and offline retail prices of pharmaceutical retailers are


$$\begin{array}{l} p_{1}^{\text{RS}*}=\frac{{\text{A}}_{2}+{\text{B}}_{2}{\text{C}}_{2}}{1-{\text{B}}_{2}{\text{D}}_{2}}\\ p_{2}^{\text{RS}*}={\text{C}}_{2}+\frac{{\text{D}}_{2}\left({\text{A}}_{2}+{\text{B}}_{2}{\text{C}}_{2}\right)}{1-{\text{B}}_{2}{\text{D}}_{2}} \end{array}$$ (9)

The price at which a pharmaceutical manufacturer sells in the direct sales channel is


$$p_{3}^{\text{RS}*}={\text{E}}_{2}+\frac{\left(1-\lambda \right)a_{3}+w\left(\gamma_{2}\text{+}\gamma_{3}\right)}{\text{2}f_{3}\left(1-\lambda \right)}$$ (10)

where


$$\{\begin{array}{l} {\text{A}}_{2}=\frac{\left(1-\lambda \right)\left[\text{2}f_{3}a_{1}+w\theta \left[\text{2}f_{3}f_{1}-\gamma_{2}^{2}-\left(1-\lambda \right)\left(\text{2}f_{3}\gamma_{1}+\gamma_{2}\gamma_{3}\right)\right]\right]+\gamma_{2}\left[\left(1-\lambda \right)a_{3}+w\left(\gamma_{2}\text{+}\gamma_{3}\right)\right]}{2\theta \left(1-\lambda \right)\left(\text{2}f_{3}f_{1}-\gamma_{2}^{2}\right)}\\ {\text{B}}_{2}=\frac{\left[\text{1+}\theta \left(1-\lambda \right)\right]\left(\text{2}f_{3}\gamma_{1}+\gamma_{2}\gamma_{3}\right)}{2\theta \left(\text{2}f_{3}f_{1}-\gamma_{2}^{2}\right)}\\ {\text{C}}_{2}=\frac{\left(1-\lambda \right)\left[\text{2}f_{3}a_{2}+\text{w}\left(\text{2}f_{3}f_{2}-\gamma_{3}^{2}\right)+\gamma_{3}a_{3}\right]+w\left(\gamma_{3}^{2}-\text{2}f_{3}\gamma_{1}\right)}{2\left(1-\lambda \right)\left(\text{2}f_{3}f_{2}-\gamma_{3}^{2}\right)}\\ {\text{D}}_{2}=\frac{\text{2}f_{3}\gamma_{1}+\gamma_{2}\gamma_{3}+\theta \left(\text{2}f_{3}\gamma_{1}\text{+}\gamma_{2}\right)\left(1-\lambda \right)}{2\left(1-\lambda \right)\left(\text{2}f_{3}f_{2}-\gamma_{3}^{2}\right)}\\ {\text{E}}_{2}=\frac{\theta \gamma_{2}}{\text{2}f_{3}}\frac{{\text{A}}_{2}{\text{+B}}_{2}{\text{C}}_{2}}{1-{\text{B}}_{2}{\text{D}}_{2}}\text{+}\frac{\gamma_{3}}{\text{2}f_{3}}\left[{\text{C}}_{2}+\frac{{\text{D}}_{2}\left({\text{A}}_{2}{\text{+B}}_{2}{\text{C}}_{2}\right)}{1-{\text{B}}_{2}{\text{D}}_{2}}\right] \end{array}$$ (11)

Further, bringing $p_{1}^{\text{RS}*}$, $p_{2}^{\text{RS}*}$, and $p_{3}^{\text{RS}*}$ back into the equation yields the optimal profits $\pi_{\text{R}}^{\text{RS}*}$ and $\pi_{\text{M}}^{\text{RS}*}$ for the pharmaceutical retailer and pharmaceutical manufacturer, which are not shown here due to the complexity of the equation.

### 4.3 Manufacturer-dominated Stackelberg game model (Model MS)

In the model MS, the pharmaceutical manufacturer, as the dominant player in the pharmaceutical supply chain, has the dominant decision-making power. Such as Pfizer, Johnson & Johnson and other large international pharmaceutical research and development and production enterprises, such enterprises research and development and production of original research and development pharmaceuticals have a very high degree of irreplaceability, so that they are in a strong position in the pharmaceutical supply chain. The objective functions of the pharmaceutical manufacturer and the pharmaceutical retailer are


$$\begin{array}{l} \underset{p_{3}}{\max}V_{\text{M}}^{\text{MS}}=p_{3}\left(1-\lambda \right)\left(a_{3}-f_{3}p_{3}+\theta \gamma_{2}p_{1}+\gamma_{3}p_{2}\right)+w\left(a_{1}-\theta f_{1}p_{1}+\gamma_{1}p_{2}+\gamma_{2}p_{3}+a_{2}-f_{2}p_{2}+\theta \gamma_{1}p_{1}+\gamma_{3}p_{3}\right)\\ \underset{p_{1},p_{2}}{\max}V_{\text{R}}^{\text{MS}}=\left(p_{1}-w-c_{1}\right)\left(a_{1}-\theta f_{1}p_{1}+\gamma_{1}p_{2}+\gamma_{2}p_{3}\right)+\left(1-\lambda \right)\left(p_{2}-w-c_{2}\right)\left(a_{2}-f_{2}p_{2}+\theta \gamma_{1}p_{1}+\gamma_{3}p_{3}\right) \end{array}$$ (12)

**Proposition 3** In this scenario, the online and offline retail prices of the pharmaceutical retailers are


$$\begin{array}{l} p_{1}^{\text{MS}*}={\text{A}}_{3}+{\text{B}}_{3}\left({\text{E}}_{3}+{\text{F}}_{3}\right)\\ p_{2}^{\text{MS}*}={\text{C}}_{3}+{\text{D}}_{3}\left({\text{E}}_{3}+{\text{F}}_{3}\right) \end{array}$$ (13)

The direct sales channel of the pharmaceutical manufacturer sells at


$$p_{3}^{\text{M S}*}={\text{E}}_{3}+{\text{F}}_{3}$$ (14)

where


$$\{\begin{array}{l} {\text{A}}_{3}=\frac{2f_{2}\left(1-\lambda \right)\left[a_{1}-\theta \gamma_{1}\left(1-\lambda \right)w+\theta f_{1}w\right]+\gamma_{1}\left[1+\theta \left(1-\lambda \right)\right]\left[a_{2}\left(1-\lambda \right)-\gamma_{1}w+f_{2}\left(1-\lambda \right)w\right]}{4\theta f_{1}f_{2}\left(1-\lambda \right)-\gamma_{1}^{2}{\left[1+\theta \left(1-\lambda \right)\right]}^{2}}\\ {\text{B}}_{3}=\frac{\left(1-\lambda \right)\left[1+\theta \left(1-\lambda \right)\right]\gamma_{1}\gamma_{3}+2\gamma_{2}f_{2}\left(1-\lambda \right)}{4\theta f_{1}f_{2}\left(1-\lambda \right)-\gamma_{1}^{2}{\left[1+\theta \left(1-\lambda \right)\right]}^{2}}\\ {\text{C}}_{3}=\frac{a_{2}\left(1-\lambda \right)-\gamma_{1}w+f_{2}\left(1-\lambda \right)w+\gamma_{1}\left[1+\theta \left(1-\lambda \right)\right]{\text{A}}_{3}}{2f_{2}\left(1-\lambda \right)}\\ {\text{D}}_{3}=\frac{\gamma_{1}\left[1+\theta \left(1-\lambda \right)\right]{\text{B}}_{3}+\gamma_{3}\left(1-\lambda \right)}{2f_{2}\left(1-\lambda \right)}\\ {\text{E}}_{3}=\frac{w\left[\left(\gamma_{1}-f_{1}\right)\theta {\text{B}}_{3}+\left(\gamma_{1}-f_{2}\right){\text{D}}_{3}+\gamma_{2}+\gamma_{3}\right]}{2\left(1-\lambda \right)\left(f_{3}-\theta \gamma_{2}{\text{B}}_{3}-\gamma_{3}{\text{D}}_{3}\right)}\\ {\text{F}}_{3}=\frac{a_{3}+\theta \gamma_{2}{\text{A}}_{3}+\gamma_{3}{\text{C}}_{3}}{2\left(f_{3}-\theta \gamma_{2}{\text{B}}_{3}-\gamma_{3}{\text{D}}_{3}\right)} \end{array}$$ (15)

Further, bringing, and $p_{3}^{\text{MS}*}$ back into the equation yields the optimal profits $\pi_{\text{R}}^{\text{MS}*}$ and $\pi_{\text{M}}^{\text{MS}*}$ for the pharmaceutical retailer and pharmaceutical manufacturer, which are not shown here due to the complexity of the equation.

Propositions 1–3 show that: under the three game models of N, RS, and MS, the retail prices of the physical, online, and direct sales channels are all jointly affected by the triple price competition intensity *γ*, the platform commission percentage *λ*, and the health insurance reimbursement percentage 1−θ. (1) Price competition between any two channels, although there is no direct participation of the third channel, but all will have a different degree of impact on the retail pricing of the third channel, so the formulation and adjustment of the retail price of pharmaceuticals should fully take into account the existence of hidden price competition between different sales channels; (2) Although platform commissions are only charged for both online channels and direct sales channels, they not only affect the retail pricing of pharmaceuticals in online sales channels, but also have different degrees of impact on the pricing of pharmaceuticals in brick-and-mortar channels due to the increase in the costs of online sales channels as a result of platform commissions; (3) Currently, China’s health insurance reimbursement policy is only for pharmaceuticals sold in physical channels and does not apply to retailers’ online channels and manufacturers’ direct sales channels. However, in the three game models, since the price concessions brought by health insurance reimbursement will expand the consumer market in physical channels and affect the profits of pharmaceutical manufacturers and pharmacies, the retail prices of online and direct sales channels should be formulated by taking into full consideration of the impact of the health insurance factor, and developing a more Therefore, retail price setting in online and direct sales channels should fully consider the impact of health insurance factors and develop more attractive pricing strategies to attract consumers in physical channels.

## 5 Analysis of numerical examples

In view of the complexity of the above theoretical results, this section will combine the above model assumptions and the actual situation of the pharmaceutical industry, through the assignment of exogenous variables to carry out the arithmetic simulation, visually compare the pharmaceutical pricing strategy and pharmaceutical supply chain performance under the three game models, and analyze and get the managerial insights. Considering the validity and generality of all models, the basic parameters are set as follows[34,36,40], drawing on the parameter assignments of related literature: a=100, ϕ=0.6, Φ=0.5, β=0.1, $k_{1}=0.8$, $k_{2}=0.9$, $k_{3}=0.9$, λ=0.5, $\gamma_{1}=0.\text{5}$, $\gamma_{2}=0.\text{5}$, $\gamma_{3}=0.\text{5}$, θ=0.3.

### 5.1 Impact of changes in health insurance reimbursement rates on pharmaceutical pricing and profits

**Fig 2:**
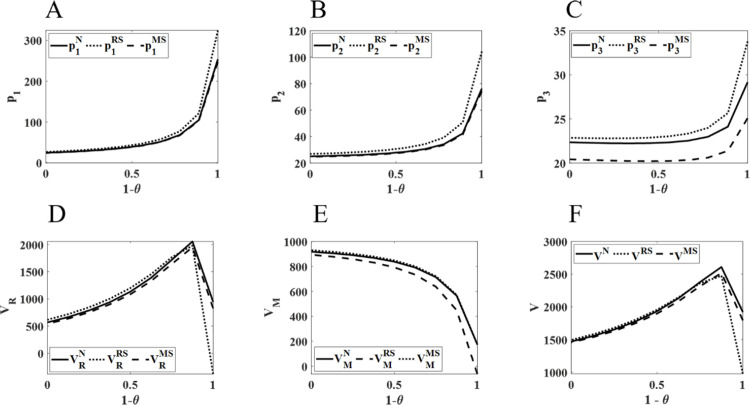
Impact of changes in health insurance reimbursement rates on pharmaceutical pricing and profits. (A) Retail prices in the physical channel; (B) Online channel retail prices; (C) Retail prices for direct sales channels; (D) Pharmaceutical retailer profits; (E) Pharmaceutical manufacturer profits; (F) Pharmaceutical supply chain total profits.

As shown in Fig 2, under different channel power conditions, the retail prices of each channel increase with the rise in health insurance reimbursement rates. When the reimbursement rate is low, the increase in retail prices is relatively gradual. However, once the reimbursement rate exceeds a certain threshold, the retail prices exhibit an exponential growth trend, with the pricing change in the physical channels being the most significant. Additionally, both the profit of pharmaceutical retailers and the total supply chain profit increase as the reimbursement rate rises. However, when the reimbursement rate becomes excessively high, both profits experience a sharp decline. In contrast, the pharmaceutical manufacturer’s profit continuously decreases.

It is evident that as the health insurance reimbursement rate increases, the price advantage of pharmaceuticals sold through physical channels gradually emerges. The lower purchase price after insurance reimbursement allows greater room for price increases in the retail prices of pharmaceuticals in physical channels. To increase profitability, physical channels gradually raise the retail prices of pharmaceuticals. Meanwhile, due to the reduced out-of-pocket costs for patients, more patients opt to purchase pharmaceuticals through physical channels, thus expanding the market share of physical channels. This results in a significant decline in sales for online and direct sales channels, and the profits of pharmaceutical manufacturers continue to decline. Although the increase in the reimbursement rate significantly boosts sales in physical channels, thereby enhancing the overall profit of pharmaceutical retailers and the supply chain, when the reimbursement rate becomes excessively high, competition between channels intensifies, leading to varying degrees of profit decline for the supply chain and retailers. Given the national 15% markup restriction on retail prices, the actual retail prices and profits for each member of the supply chain will fall below the theoretical values shown in Fig 2, potentially forcing some companies to exit the market. Therefore, when formulating health insurance reimbursement policies, the government should carefully consider the price competition between physical, online, and direct sales channels to ensure the profitability of pharmaceutical manufacturers and retailers. Additionally, the determination of pharmaceutical retail prices should closely align with health insurance reimbursement policies, fully considering the impact of these policies on price adjustments.

### 5.2 The impact of changes in competitive intensity on pharmaceutical pricing and profits

**Fig 3:**
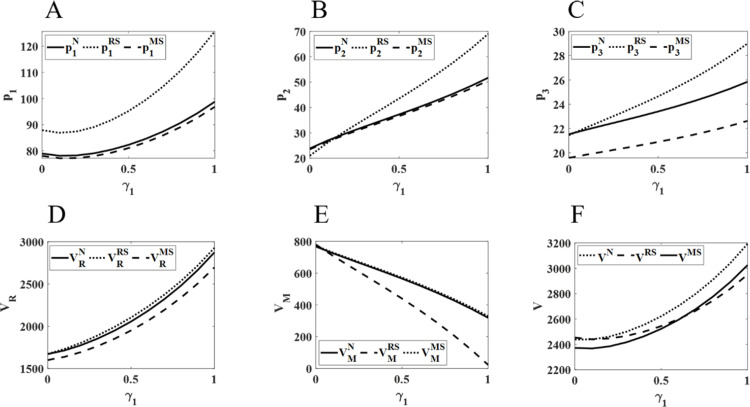
Impact of changes in $\gamma_{1}$ on pharmaceutical pricing and profits. The diagram (ABCDEF) represents the same meaning as Fig 2.

Fig 3 shows that as the competition between physical and online channels intensifies, the retail prices of online and direct sales channels exhibit an upward trend. Conversely, the retail price in physical channels first decreases and then increases. Meanwhile, the overall profits of pharmaceutical retailers and the supply chain show a growth trend, while the profits of manufacturers consistently decline. Among all channels, the retail price increase is most prominent in online channels. The price gap between physical and online channels gradually narrows. Additionally, pharmaceutical retailers achieve higher profits under the RS model, while their profits are lower under the MS model. In contrast, manufacturers’ profits are higher under the MS model and lower under the RS model. Finally, the total supply chain profit is most significant under the N model.

As competition between the two channels within the retailer (physical and online channels) intensifies, the online channel, with its lower sales costs and convenient purchasing methods, maintains a lower and continuously rising retail price compared to the physical channel. Among the three channels, the online channel experiences the largest price increase. However, the introduction of the online channel leads to a loss of some customers from the physical channel. When competition is weak, the physical channel attempts to maintain customer traffic through price reductions, but the impact of this measure is limited, thus not significantly harming the retailer’s profits. As competition intensifies, retailers are forced to adjust their pricing, raising the retail price of the physical channel. However, the price increase in physical channels is smaller than in the online channel in order to balance the competition between the two sales channels, reduce their negative impact, and increase profitability. However, the internal competition between the two channels within the retailer is disadvantageous to the manufacturer. The pricing and sales coordination between the two channels lead to a substantial loss of direct sales channel customers, resulting in a decline in the manufacturer’s profits. Despite the manufacturer raising the retail prices of the direct sales channel, the downward profit trend remains unchanged. Therefore, pharmaceutical manufacturers should increase wholesale prices and reasonably adjust retail prices to attract more consumers, minimize the negative effects of competition, and thereby improve sales and their own profits. Finally, as the retail prices of each channel continue to rise, the total supply chain profit gradually increases. Since each party in the pharmaceutical supply chain can obtain higher profits when they hold a dominant position and lower profits when they are in a weaker position, the total supply chain profit reaches its highest when there is a balance in the power between the two channels.

**Fig 4:**
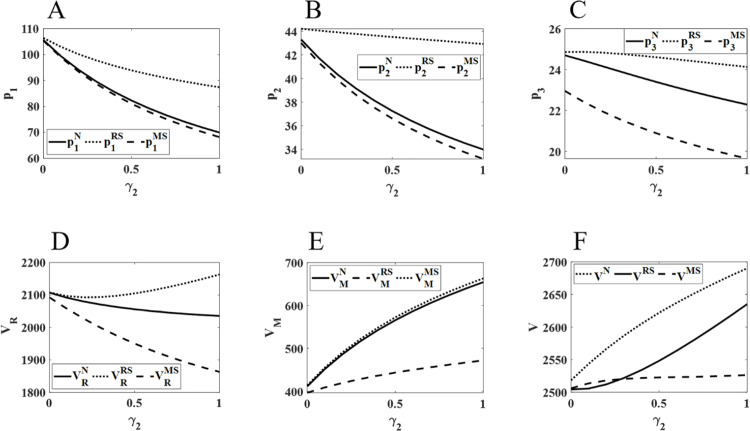
Impact of changes in $\gamma_{2}$ on pharmaceutical pricing and profits. The diagram (ABCDEF) represents the same meaning as Fig 2.

As shown in Fig 4, as the intensity of competition between the physical and direct sales channels increases, pharmaceutical retail prices across all three sales channels decrease to varying extents. Specifically, under the RS model, the retail prices in each channel are the highest, with the smallest decline. Furthermore, both manufacturer profits and total supply chain profits exhibit an upward trend. However, retailer profits show growth only under the RS model, while they decline to different degrees under the N and MS models. Additionally, compared to the other models, retailer profits are higher under the RS model and lower under the MS model. Manufacturer profits exhibit an opposite trend to that of retailers. Total supply chain profits consistently remain higher under the RS model.

It is evident that in the price competition between the physical and direct sales channels, the direct sales channel, benefiting from its price advantage, has captured a larger share of the market. This has impacted the sales of both physical and online channels to varying extents. Additionally, this has caused a decline in the profits of retailers with weaker channel power, compelling them to reduce retail prices in both the physical and online channels in order to sustain sales and profits. However, under the RS model, pharmaceutical retailers hold a dominant position within the supply chain, enjoying stronger channel power. As a result, the negative impact of the direct sales channel is minimal, and their profits exhibit a steady increase. Furthermore, as price competition between the physical and direct sales channels intensifies, retailers continue to lower the retail prices in both channels. This weakens the price advantage of pharmaceutical manufacturers. To maintain competitiveness, manufacturers are forced to slightly reduce retail prices in the direct sales channel to ensure profit growth. Finally, within the pharmaceutical supply chain, when one party holds a dominant position, its stronger channel power and control over decision-making enable it to generate higher profits, which significantly negatively impacts the profits of the other party. Therefore, the total supply chain profit reaches its maximum only when the channel powers of both parties are balanced.

**Fig 5:**
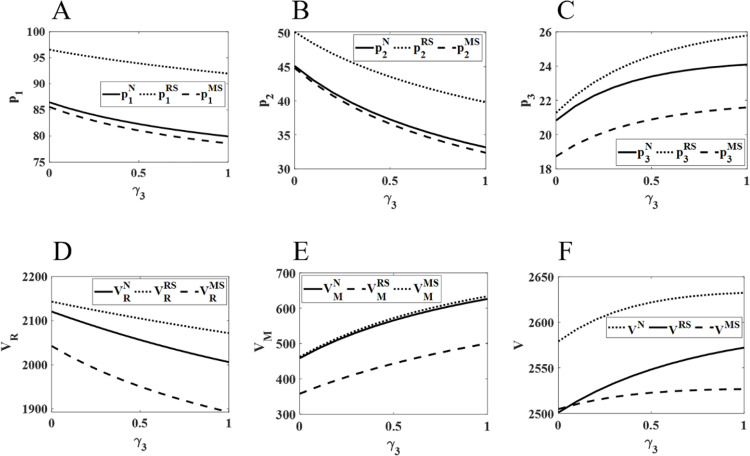
Impact of changes in $\gamma_{3}$ on pharmaceutical pricing and profits. The diagram (ABCDEF) represents the same meaning as Fig 2.

Fig 5 illustrates that as competition between the online and direct sales channels intensifies, both the retail prices of the physical and online channels and the profits of pharmaceutical retailers show a declining trend. In contrast, the retail price of the direct sales channel continues to rise, with the online channel experiencing the most significant fluctuations. Additionally, as competition increases, the profits of pharmaceutical retailers consistently decrease, while the profits of manufacturers and the total supply chain profits rise. Among the three models, retailer profits, manufacturer profits, and total supply chain profits are highest under the RS, MS, and N models, respectively.

It can be inferred that in the price competition between online and direct sales channels, the direct sales channel holds a significant price advantage due to its lower cost structure. This has attracted a large number of patients who prefer online sales channels to purchase lower-priced pharmaceuticals through direct sales, thereby negatively impacting the sales of the online channel. Moreover, the price competition between online sales channels has expanded the overall consumer market, resulting in a continuous decline in physical channel sales and a sustained reduction in retailer profits. Consequently, to attract customers, retailers are compelled to lower the retail prices of pharmaceuticals in both the online and physical channels, aiming to increase sales and safeguard profitability. Nevertheless, the direct sales channel still has some room for price increases amidst fierce price competition, with its retail price steadily rising and profits continuing to grow. Finally, when retailers hold a dominant position in the market, their stronger channel power enables them to set higher retail prices, generating higher profits for themselves and the entire supply chain.

### 5.3 The impact of changes in competitive strength of retailers’ online channels on pharmaceutical pricing and profits

As mentioned in the hypothesis, parameter $\gamma_{1}$ measures the degree of price competition between the online channel and the physical channel, and $\gamma_{3}$ measures the degree of price competition between the online channel and the direct sales channel. Compared to the other two channels, the weaker the channel competition strength of the online channel, the lower $\gamma_{1}$ and $\gamma_{3}$; the stronger the channel competition strength of the online channel, the higher $\gamma_{1}$ and $\gamma_{3}$; and when the online channel withdraws from the competitive ranks of the pharmaceutical sales channel, i.e., retailers do not open the online channel to participate in the sale of pharmaceuticals and the price competition, and the sales of pharmaceuticals in the other two channels are not affected by the online channel, then $\gamma_{1}$ and $\gamma_{3}$ are both zero. Therefore, in this section, $\gamma_{1}$ and $\gamma_{3}$ are used as independent variables to compare and analyze the impact of the opening of retailers’ online channels and the changes in their competitive strengths on the pricing and profitability of pharmaceuticals across all channels of the pharmaceutical supply chain in a more intuitive manner by using MATLAB three-dimensional plotting.

**Fig 6:**
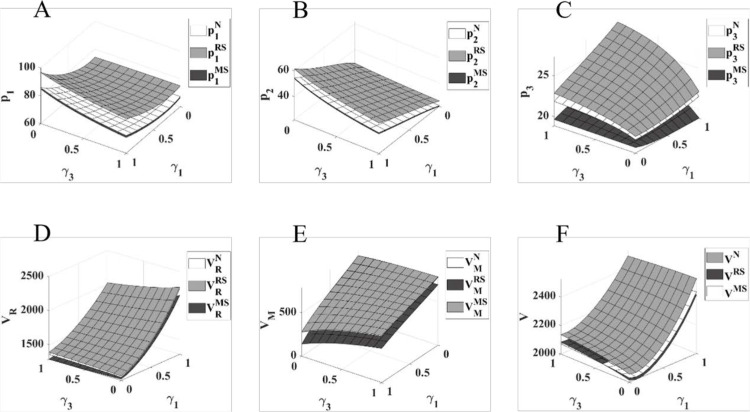
Impact of changes in competitive strength of online channels on pharmaceutical pricing and profits. The diagram (ABCDEF) represents the same meaning as Fig 2.

As shown in Fig 6, in the three game models of N, RS, and MS, with the improvement of $\gamma_{1}$ and $\gamma_{3}$, the retail price of physical channels and direct sales channels as a whole shows a growing trend, and the retail price of online channels as a whole shows a decreasing trend; the profit of pharmaceutical retailers with the improvement of $\gamma_{1}$ and $\gamma_{3}$ as a whole shows a growing trend, and the growth rate is relatively obvious, although the profit of pharmaceutical manufacturers with the improvement of $\gamma_{1}$ and $\gamma_{3}$ as a whole shows a decreasing trend, but due to the small decrease, the total profit of the pharmaceutical Although the profit of pharmaceutical manufacturers is decreasing with the increase of $\gamma_{1}$ and $\gamma_{3}$, due to the smaller decrease, the total profit of the pharmaceutical supply chain has increased considerably with the increase of $\gamma_{1}$ and $\gamma_{3}$.

In summary, Fig 6 visualizes the impact of the retailer online channel and changes in the competitive power of this sales channel on pharmaceutical pricing and supply chain performance in each channel under different channel power, using $\gamma_{1}$ and $\gamma_{3}$ as independent variables. Specifically, the opening of retailers’ online channels increases the flexibility of retailers’ pharmaceutical sales, expands the retailers’ consumer market, and breaks the monopoly of manufacturers’ direct sales channels over the online sales market. At the same time, the manufacturer’s channel power is weakened, and the impact of the manufacturer’s direct channel on the sales of the physical channel is offset to a certain extent, creating higher profits for the retailer and the supply chain. Pharmaceutical manufacturers can avoid the negative impact of online channels by improving their channel power and the competitive strength of their direct sales channels due to a slight decline in profits from lower sales. In addition, as the competitive power of online channels gradually increases, retail prices of pharmaceuticals have increased to different degrees in all channels, with retailer-led retail prices being the highest, manufacturer-led retail prices being the lowest, and retail prices in online channels increasing the most.

### 5.4 The impact of changes in the competitive strength of manufacturers’ direct distribution channels on pharmaceutical pricing and profits

As mentioned in the hypothesis, parameter $\gamma_{2}$ measures the degree of price competition between the direct sales channel and the physical channel, and $\gamma_{3}$ measures the degree of price competition between the direct sales channel and the online channel. Compared to the other two channels, the weaker the channel competition strength of the manufacturer’s direct sales channel, the lower $\gamma_{2}$ and $\gamma_{3}$ are; the stronger the channel competition strength of the direct sales channel, the higher $\gamma_{2}$ and $\gamma_{3}$ are; and when the direct sales channel withdraws from the competitive ranks of the pharmaceutical sales channel, i.e., the manufacturer has not opened the direct sales channel to participate in the sale of pharmaceuticals and the price competition, and the sale of pharmaceuticals in the other two channels is not affected by the direct sales channel, then both $\gamma_{2}$ and $\gamma_{3}$ are zero. Therefore, in this section, $\gamma_{2}$ and $\gamma_{3}$ are used as independent variables to compare and analyze the impact of the opening of the manufacturer’s direct sales channel and the changes in its competitive strength on the pricing and profitability of pharmaceuticals across all channels of the pharmaceutical supply chain in a more visual and comparative manner by using MATLAB three-dimensional plotting.

**Fig 7:**
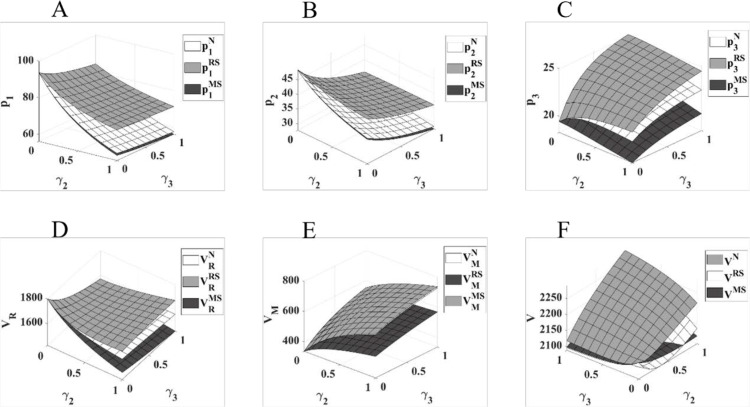
Impact of changes in competitive strength of direct marketing channels on pharmaceutical pricing and profits. The diagram (ABCDEF) represents the same meaning as Fig 2.

Fig 7 shows that: in the three game models of N, RS and MS, with the improvement of $\gamma_{2}$ and $\gamma_{3}$, the retail price of physical channels and online channels as a whole shows a decreasing trend, and the price of direct sales channels as a whole shows a growing trend; the profit of retailers as a whole has a small decrease with the improvement of $\gamma_{2}$ and $\gamma_{3}$, and the profit of manufacturers as a whole shows a growing trend with the improvement of $\gamma_{2}$ and $\gamma_{3}$. With the improvement of $\gamma_{2}$ and $\gamma_{3}$, the total profit of the pharmaceutical supply chain under the N and MS models shows a different magnitude, while the total profit of the pharmaceutical supply chain under the RS model shows a decreasing and then increasing trend. With the increase of $\gamma_{2}$ and $\gamma_{3}$, the total profit of pharmaceutical supply chain under the N and MS models shows an overall increase of different magnitudes, while the total profit of pharmaceutical supply chain under the RS model shows an overall trend of decreasing and then increasing.

In summary, Fig 7 visualizes the impact of the manufacturer’s direct sales channel and changes in the competitive strength of that sales channel on pharmaceutical pricing and supply chain performance across channels, using $\gamma_{2}$ and $\gamma_{3}$ as independent variables. Specifically, compared to the two channels within the retailer, the manufacturer’s direct sales channel sells pharmaceuticals at a lower price due to the lack of retail markups in the distribution chain and the higher cost of sales in the physical channel. As a result, the opening of direct sales channels reduces the consumer market occupied by retailers to a greater extent, forcing retailers to adjust downward the retail prices of their two internal sales channels in order to narrow the retail price difference with the direct sales channels, thus increasing sales volume and safeguarding profits. With the gradual increase in the competitive strength of the direct sales channel, in addition to a small increase in the retail price of the direct sales channel, the retail price of both channels within the retailer gradually decreased, with the biggest decrease in the retail price of the brick-and-mortar channel. The opening of direct sales channels has therefore reduced the maximum market price of pharmaceuticals on sale, greatly improving the accessibility of pharmaceuticals and the level of medical protection for China’s residents. The opening of direct sales channels has also increased the profitability of manufacturers and expanded the market for online sales channels for pharmaceutical products, which is favorable to the profitability of manufacturers and the overall profitability of the supply chain. For pharmaceutical retailers, who are affected by the cutbacks in the consumer market and whose profits are decreasing, they can seek to dominate the pharmaceutical supply chain by improving their own channel power and maximizing their returns in an unfavorable competitive environment.

## 6 Conclusions and managerial implications

This paper combines the triple price competition among sales channels and health insurance reimbursement policy and other factors to construct a game model of N, RS, and MS with three different channel powers, and analyzes the intensity of inter-channel competition, changes in health insurance reimbursement ratios, and the impact of different online sales channels on the pricing of pharmaceuticals and supply chain performance of each channel under different channel powers.

### 6.1 Conclusions

First, channel power affects the profits of pharmaceutical supply chain members and the pricing of pharmaceuticals in each channel. When the retailer is more powerful and dominant in the pharmaceutical supply chain, the pricing of pharmaceuticals in each channel is higher; because stronger channel power can effectively reduce the negative impact of price competition on itself, the profit of either the retailer or the manufacturer is higher when it is in a dominant position, and the total profit of the pharmaceutical supply chain is higher when the channel power of both members is equal.

Second, as the reimbursement rate of health insurance increases, the profit of pharmaceutical retailers and the pricing of pharmaceuticals in each channel will increase, but there is a certain negative impact on pharmaceutical manufacturers; while when the reimbursement rate is too high, the total profit of the supply chain and the profit of each member of the supply chain have a relatively large decline due to the intensification of channel competition.

Third, price competition between channels does not always reduce the retail price of pharmaceuticals for pharmaceutical retailers. Price competition between direct sales channels and online or physical channels can effectively reduce the retail price of pharmaceuticals for pharmaceutical retailers, while price competition between online and physical channels has the opposite effect and is not conducive to reducing the burden of pharmaceuticals on patients.

Fourth, while retailers’ expansion into online channels can increase their own profits and contribute to the overall profit growth of the pharmaceutical supply chain, it also suppresses the profitability of pharmaceutical manufacturers and is detrimental to reducing pharmaceutical retail prices. In contrast, pharmaceutical manufacturers can effectively lower the retail prices of pharmaceuticals in both physical and online channels by opening direct sales channels. This approach not only narrows the retail price gap between channels and alleviates the financial burden on patients but also mitigates the negative impact of online channels on manufacturers’ profits.

### 6.2 Managerial implications

Based on the above conclusions, the following recommendations are made for pharmaceutical supply chain-related enterprises and departments:

First of all, considering the impact of channel power on the profits of supply chain members, pharmaceutical retailers should continuously explore new sales channels to reach a broader consumer base. For instance, in recent years, Ali Health and JD Pharmacy have successfully attracted a large number of young consumers through the development of online stores and mobile apps. This not only expanded their sales reach but also enhanced their bargaining power. Similarly, pharmaceutical manufacturers should focus on brand development and R&D investment to increase the irreplaceability of their own products. For example, Hengrui Pharmaceuticals has consistently launched innovative pharmaceuticals through substantial investments in research and development. This not only enhanced the company’s market competitiveness and strengthened its brand position, but also increased its negotiation leverage with retailers, thereby boosting its profits within the supply chain.

Secondly, the cost advantages of direct sales channels for manufacturers have caused significant disruption to both retail physical and online channels. Manufacturers should actively explore cooperative policies such as revenue sharing and cost subsidies, based on their own circumstances. These policies can effectively motivate retailers to improve their pharmaceutical sales efforts, enhance service levels, and ultimately increase pharmaceutical sales, leading to greater profits.

Finally, the medical insurance reimbursement policy for pharmaceuticals significantly influences consumers’ preferences for purchasing channels, which in turn affects the market sales volume of pharmaceuticals across different channels and the profits of supply chain members. To further promote the healthy and sustainable development of the pharmaceutical industry, relevant authorities should fully consider the impact of reimbursement policies on market structure and establish reasonable reimbursement rates. For example, the Shanghai Municipal Medical Security Bureau successfully expanded the coverage of cancer pharmaceuticals under medical insurance by adjusting its reimbursement policy. This made these pharmaceuticals more affordable for a larger number of consumers, while also driving sales growth for pharmaceutical manufacturers. Additionally, relevant departments should further promote the widespread adoption of online medical insurance payment functions (which are already being piloted in some cities) to reduce the negative competitive impacts between online and offline channels.

### 6.3 Future research directions

This study has some limitations and areas for further expansion. Firstly, regarding the conclusion that the establishment of direct sales channels by pharmaceutical manufacturers intensifies price competition across various channels, future research could build upon this study by designing coordination contracts to align the performance of supply chain members. These contracts could help motivate pharmaceutical retailers to actively engage in pharmaceutical sales, thereby achieving a win-win outcome. Secondly, with the growing adoption of omnichannel capabilities in pharmaceutical payment systems, future research could explore the impact of varying channel power and competitiveness on the overall performance and development of the pharmaceutical supply chain. Finally, this paper primarily focuses on the OTC pharmaceutical supply chain and does not delve deeply into the prescription pharmaceutical supply chain. Given the differences between the two, future studies could investigate pricing strategies and dynamics within the prescription pharmaceutical supply chain.

## Supporting information

S1 File This file contains the complete source code used to generate all the graphs in the numerical examples section of the text. (PDF)
