# Supplementary material for: Multi-channel pricing strategies for pharmaceutical supply chains considering channel power and price competition
Source: PLoS One. 2025 May 2;20(5):e0322143. doi: 10.1371/journal.pone.0322143 (PMC12047794; doi:10.1371/journal.pone.0322143)
Supplement: S1 File — (PDF) [file pone.0322143.s001.pdf]

Original Data Code

S1 Fig 2. (A) Code

```
o=0.1:0.1:1;
A1=[2.*1.9.*0.6.*[40-o.*0.5.*0.6.*15+o.*1.8.*15]+0.5.*(1+o.*0.6).*[35.*0.6-
0.5.*15+1.9.*0.6.*15]]./[4.*o.*1.8.*1.9.*0.6-0.5.*0.5.*(1+o.*0.6).*(1+o.*0.6)]
B1=[0.6.*[(1+o.*0.6).*0.5.*0.5+2.*0.5.*1.9.*0.6]]./[4.*o.*1.8.*1.9.*0.6-
0.5.*0.5.*(1+o.*0.6).*(1+o.*0.6)]
C1=[35.*0.6-0.5.*15+1.9.*0.6.*15+0.5.*(1+o.*0.6).*A1]./[2.*1.9.*0.6]
D1=[0.5.*(1+o.*0.6).*B1+0.5.*0.6]./[2.*1.9.*0.6]
A2=[0.6.*[2.*1.9.*40+15.*o.*[2.*1.9.*1.8-0.5.*0.5-
0.6.*[2.*1.9.*0.5+0.5.*0.5]]]+0.5.*[0.6.*35+15.*(0.5+0.5)]]./[2.*o.*0.6.*[2.*1.9.*1.8
-0.5.*0.5]]
B2=[[1+o.*0.6].*[2.*1.9.*0.5+0.5.*0.5]]./[2.*o.*(2.*1.8.*1.9-0.5.*0.5)]
C2=[0.6.*[2.*1.9.*35+15.*[2.*1.9.*1.9-0.5.*0.5]+0.5.*35]+15.*[0.5.*0.5-
2.*1.9.*0.5]]./[2.*0.6.*[2.*1.9.*1.9-0.5.*0.5]]
D2=[2.*1.9.*0.5+0.5.*0.5+o.*[2.*1.9.*0.5+0.5].*0.6]./[2.*0.6.*(2.*1.9.*1.9-
0.5.*0.5)]
E2=[o.*0.5./(2.*1.9)].*[A2+B2.*C2]./[1-B2.*D2]+0.5.*[C2+[D2.*(A2+B2.*C2)./(1-
B2.*D2)]]./(2.*1.9)
A3=[2.*1.9.*0.6.*[40-o.*0.5.*0.6.*15+o.*1.8.*15]+0.5.*(1+o.*0.6).*[35.*0.6-
0.5.*15+1.9.*0.6.*15]]./[4.*o.*1.8.*1.9.*0.6-0.5.*0.5.*(1+o.*0.6).*(1+o.*0.6)]
B3=[0.6.*[(1+o.*0.6).*0.5.*0.5+2.*0.5.*1.9.*0.6]]./[4.*o.*1.8.*1.9.*0.6-
0.5.*0.5.*(1+o.*0.6).*(1+o.*0.6)]
C3=[35.*0.6-0.5.*15+1.9.*0.6.*15+0.5.*(1+o.*0.6).*A3]./[2.*1.9.*0.6]
D3=[0.5.*(1+o.*0.6).*B3+0.5.*0.6]./[2.*1.9.*0.6]
E3=15.*[(-o.*1.8.*B3+0.5.*D3+0.5)+o.*0.5.*B3-1.9.*D3+0.5]./[2.*0.6.*[1.9-
o.*0.5.*B3-0.5.*D3]]
F3=[35+o.*0.5.*A3+0.5.*C3]./[2.*[1.9-o.*0.5.*B3-0.5.*D3]]
P1N=A1+B1.*[0.6.*[35+o.*0.5.*A1+0.5.*C1]+15.*[0.5+0.5]]./[0.6.*[2.*1.9-
o.*0.5.*B1-0.5.*D1]]
P1R=(A2+B2.*C2)./(1-B2.*D2)
P1M=A3+B3.*[E3+F3]
% 映射横坐标到 1-0
theta = 1 - ((o - min(o)) / (max(o) - min(o)));
plot(theta, P1N, '--k', theta, P1R, 'k', theta, P1M, ':k');
xlabel('1-\theta');
ylabel('p_1');
legend('p_1^N', 'p_1^{RS}', 'p_1^{MS}');
xlim([0 1]);
```

S1 Fig 2. (B) Code

```
o=0.1:0.1:1;
A1=[2.*1.9.*0.6.*[40-o.*0.5.*0.6.*15+o.*1.8.*15]+0.5.*(1+o.*0.6).*[35.*0.6-
0.5.*15+1.9.*0.6.*15]]./[4.*o.*1.8.*1.9.*0.6-0.5.*0.5.*(1+o.*0.6).*(1+o.*0.6)]
B1=[0.6.*[(1+o.*0.6).*0.5.*0.5+2.*0.5.*1.9.*0.6]]./[4.*o.*1.8.*1.9.*0.6-
0.5.*0.5.*(1+o.*0.6).*(1+o.*0.6)]
C1=[35.*0.6-0.5.*15+1.9.*0.6.*15+0.5.*(1+o.*0.6).*A1]./[2.*1.9.*0.6]
```

```
D1=[0.5.*(1+o.*0.6).*B1+0.5.*0.6]./[2.*1.9.*0.6]
A2=[0.6.*[2.*1.9.*40+15.*o.*[2.*1.9.*1.8-0.5.*0.5-
0.6.*[2.*1.9.*0.5+0.5.*0.5]]]+0.5.*[0.6.*35+15.*(0.5+0.5)]]./[2.*o.*0.6.*[2.*1.9.*1.8
-0.5.*0.5]]
B2=[[1+o.*0.6].*[2.*1.9.*0.5+0.5.*0.5]]./[2.*o.*(2.*1.8.*1.9-0.5.*0.5)]
C2=[0.6.*[2.*1.9.*35+15.*[2.*1.9.*1.9-0.5.*0.5]+0.5.*35]+15.*[0.5.*0.5-
2.*1.9.*0.5]]./[2.*0.6.*[2.*1.9.*1.9-0.5.*0.5]]
D2=[2.*1.9.*0.5+0.5.*0.5+o.*[2.*1.9.*0.5+0.5].*0.6]./[2.*0.6.*(2.*1.9.*1.9-
0.5.*0.5)]
E2=[o.*0.5./(2.*1.9)].*[A2+B2.*C2]./[1-B2.*D2]+0.5.*[C2+[D2.*(A2+B2.*C2)./(1-
B2.*D2)]]./(2.*1.9)
A3=[2.*1.9.*0.6.*[40-o.*0.5.*0.6.*15+o.*1.8.*15]+0.5.*(1+o.*0.6).*[35.*0.6-
0.5.*15+1.9.*0.6.*15]]./[4.*o.*1.8.*1.9.*0.6-0.5.*0.5.*(1+o.*0.6).*(1+o.*0.6)]
B3=[0.6.*[(1+o.*0.6).*0.5.*0.5+2.*0.5.*1.9.*0.6]]./[4.*o.*1.8.*1.9.*0.6-
0.5.*0.5.*(1+o.*0.6).*(1+o.*0.6)]
C3=[35.*0.6-0.5.*15+1.9.*0.6.*15+0.5.*(1+o.*0.6).*A3]./[2.*1.9.*0.6]
D3=[0.5.*(1+o.*0.6).*B3+0.5.*0.6]./[2.*1.9.*0.6]
E3=15.*[(-o.*1.8.*B3+0.5.*D3+0.5)+o.*0.5.*B3-1.9.*D3+0.5]./[2.*0.6.*[1.9-
o.*0.5.*B3-0.5.*D3]]
F3=[35+o.*0.5.*A3+0.5.*C3]./[2.*[1.9-o.*0.5.*B3-0.5.*D3]]
P2N=C1+D1.*[0.6.*[35+o.*0.5.*A1+0.5.*C1]+15.*[0.5+0.5]]./[0.6.*[2.*1.9-
o.*0.5.*B1-0.5.*D1]]
P2R=C2+[D2.*(A2+B2.*C2)]./(1-B2.*D2)
P2M=C3+D3.*[E3+F3]
% 映射横坐标到 1-0
theta = 1 - ((o - min(o)) / (max(o) - min(o)));
plot(theta, P2N, '--k', theta, P2R, 'k', theta, P2M, ':k');
xlabel('1-\theta')
ylabel('p_2');
legend('p_2^N', 'p_2^{RS}', 'p_2^{MS}');
xlim([0 1]);
```

S1 Fig 2. (C) Code

```
o=0.1:0.1:1;
A1=[2.*1.9.*0.6.*[40-o.*0.5.*0.6.*15+o.*1.8.*15]+0.5.*(1+o.*0.6).*[35.*0.6-
0.5.*15+1.9.*0.6.*15]]./[4.*o.*1.8.*1.9.*0.6-0.5.*0.5.*(1+o.*0.6).*(1+o.*0.6)]
B1=[0.6.*[(1+o.*0.6).*0.5.*0.5+2.*0.5.*1.9.*0.6]]./[4.*o.*1.8.*1.9.*0.6-
0.5.*0.5.*(1+o.*0.6).*(1+o.*0.6)]
C1=[35.*0.6-0.5.*15+1.9.*0.6.*15+0.5.*(1+o.*0.6).*A1]./[2.*1.9.*0.6]
D1=[0.5.*(1+o.*0.6).*B1+0.5.*0.6]./[2.*1.9.*0.6]
A2=[0.6.*[2.*1.9.*40+15.*o.*[2.*1.9.*1.8-0.5.*0.5-
0.6.*[2.*1.9.*0.5+0.5.*0.5]]]+0.5.*[0.6.*35+15.*(0.5+0.5)]]./[2.*o.*0.6.*[2.*1.9.*1.8
-0.5.*0.5]]
B2=[[1+o.*0.6].*[2.*1.9.*0.5+0.5.*0.5]]./[2.*o.*(2.*1.8.*1.9-0.5.*0.5)]
C2=[0.6.*[2.*1.9.*35+15.*[2.*1.9.*1.9-0.5.*0.5]+0.5.*35]+15.*[0.5.*0.5-
2.*1.9.*0.5]]./[2.*0.6.*[2.*1.9.*1.9-0.5.*0.5]]
D2=[2.*1.9.*0.5+0.5.*0.5+o.*[2.*1.9.*0.5+0.5].*0.6]./[2.*0.6.*(2.*1.9.*1.9-
0.5.*0.5)]
```

```
E2=[o.*0.5./(2.*1.9)].*[A2+B2.*C2]./[1-B2.*D2]+0.5.*[C2+[D2.*(A2+B2.*C2)./(1-B2.*D2)]]./(2.*1.9)
A3=[2.*1.9.*0.6.*[40-o.*0.5.*0.6.*15+o.*1.8.*15]+0.5.*(1+o.*0.6).*[35.*0.6-0.5.*15+1.9.*0.6.*15]]./[4.*o.*1.8.*1.9.*0.6-0.5.*0.5.*(1+o.*0.6).*(1+o.*0.6)]
B3=[0.6.*[(1+o.*0.6).*0.5.*0.5+2.*0.5.*1.9.*0.6]]./[4.*o.*1.8.*1.9.*0.6-0.5.*0.5.*(1+o.*0.6).*(1+o.*0.6)]
C3=[35.*0.6-0.5.*15+1.9.*0.6.*15+0.5.*(1+o.*0.6).*A3]./[2.*1.9.*0.6]
D3=[0.5.*(1+o.*0.6).*B3+0.5.*0.6]./[2.*1.9.*0.6]
E3=15.*[(-o.*1.8.*B3+0.5.*D3+0.5)+o.*0.5.*B3-1.9.*D3+0.5]./[2.*0.6.*[1.9-o.*0.5.*B3-0.5.*D3]]
F3=[35+o.*0.5.*A3+0.5.*C3]./[2.*[1.9-o.*0.5.*B3-0.5.*D3]]
P3N=[0.6.*[35+o.*0.5.*A1+0.5.*C1]+15.*[0.5+0.5]]./[0.6.*[2.*1.9-o.*0.5.*B1-0.5.*D1]]
P3R=E2+[0.6.*35+15.*(0.5+0.5)]./(2.*1.9.*0.6)
P3M=E3+F3
% 映射横坐标到 1-0
theta = 1 - ((o - min(o)) / (max(o) - min(o)));
plot(theta, P3N, '--k', theta, P3R, 'k', theta, P3M, ':k');
xlabel('1-\theta')
ylabel('p_3');
legend('p_3^N', 'p_3^{RS}', 'p_3^{MS}');
xlim([0 1]);
```

S1 Fig 2. (D) Code

```
o=0.15:0.1:1;
% 计算所有相关变量
A1=[2.*1.9.*0.6.*[40-o.*0.5.*0.6.*15+o.*1.8.*15]+0.5.*(1+o.*0.6).*[35.*0.6-0.5.*15+1.9.*0.6.*15]]./[4.*o.*1.8.*1.9.*0.6-0.5.*0.5.*(1+o.*0.6).*(1+o.*0.6)]
B1=[0.6.*[(1+o.*0.6).*0.5.*0.5+2.*0.5.*1.9.*0.6]]./[4.*o.*1.8.*1.9.*0.6-0.5.*0.5.*(1+o.*0.6).*(1+o.*0.6)]
C1=[35.*0.6-0.5.*15+1.9.*0.6.*15+0.5.*(1+o.*0.6).*A1]./[2.*1.9.*0.6]
D1=[0.5.*(1+o.*0.6).*B1+0.5.*0.6]./[2.*1.9.*0.6]
A2=[0.6.*[2.*1.9.*40+15.*o.*[2.*1.9.*1.8-0.5.*0.5-0.6.*[2.*1.9.*0.5+0.5.*0.5]]]+0.5.*[0.6.*35+15.*0.6]]./[2.*o.*0.6.*[2.*1.9.*1.8-0.5.*0.5]]
B2=[[1+o.*0.6].*[2.*1.9.*0.5+0.5.*0.5]]./[2.*o.*(2.*1.8.*1.9-0.5.*0.5)]
C2=[0.6.*[2.*1.9.*35+15.*[2.*1.9.*1.9-0.5.*0.5]+0.5.*35]+15.*[0.5.*0.5-2.*1.9.*0.5]]./[2.*0.6.*[2.*1.9.*1.9-0.5.*0.5]]
D2=[2.*1.9.*0.5+0.5.*0.5+o.*[2.*1.9.*0.5+0.5].*0.6]./[2.*0.6.*(2.*1.9.*1.9-0.5.*0.5)]
E2=[o.*0.5./(2.*1.9)].*[A2+B2.*C2]./[1-B2.*D2]+0.5.*[C2+[D2.*(A2+B2.*C2)./(1-B2.*D2)]]./(2.*1.9)
A3=[2.*1.9.*0.6.*[40-o.*0.5.*0.6.*15+o.*1.8.*15]+0.5.*(1+o.*0.6).*[35.*0.6-0.5.*15+1.9.*0.6.*15]]./[4.*o.*1.8.*1.9.*0.6-0.5.*0.5.*(1+o.*0.6).*(1+o.*0.6)]
B3=[0.6.*[(1+o.*0.6).*0.5.*0.5+2.*0.5.*1.9.*0.6]]./[4.*o.*1.8.*1.9.*0.6-0.5.*0.5.*(1+o.*0.6).*(1+o.*0.6)]
C3=[35.*0.6-0.5.*15+1.9.*0.6.*15+0.5.*(1+o.*0.6).*A3]./[2.*1.9.*0.6]
```

```
D3=[0.5.*(1+o.*0.6).*B3+0.5.*0.6]./[2.*1.9.*0.6]
E3=15.*[(-o.*1.8.*B3+0.5.*D3+0.5)+o.*0.5.*B3-1.9.*D3+0.5]./[2.*0.6.*[1.9-o.*0.5.*B3-0.5.*D3]]
F3=[35+o.*0.5.*A3+0.5.*C3]./[2.*[1.9-o.*0.5.*B3-0.5.*D3]]
P1N=A1+B1.*[0.6.*[35+o.*0.5.*A1+0.5.*C1]+15.*[0.5+0.5]]./[0.6.*[2.*1.9-o.*0.5.*B1-0.5.*D1]]
P1R=(A2+B2.*C2)./(1-B2.*D2)
P1M=A3+B3.*[E3+F3]
P2N=C1+D1.*[0.6.*[35+o.*0.5.*A1+0.5.*C1]+15.*[0.5+0.5]]./[0.6.*[2.*1.9-o.*0.5.*B1-0.5.*D1]]
P2R=C2+[D2.*(A2+B2.*C2)]./(1-B2.*D2)
P2M=C3+D3.*[E3+F3]
P3N=[0.6.*[35+o.*0.5.*A1+0.5.*C1]+15.*[0.5+0.5]]./[0.6.*[2.*1.9-o.*0.5.*B1-0.5.*D1]]
P3R=E2+[0.6.*35+15.*0.6]./(2.*1.9.*0.6)
P3M=E3+F3
Q1N=40-0.25.*1.8.*P1N+0.5.*P2N+0.5.*P3N
Q2N=35-1.9.*P2N+0.25.*0.5.*P1N+0.5.*P3N
Q3N=35-1.9.*P3N+0.25.*0.5.*P1N+0.5.*P2N
Q1R=40-0.25.*1.8.*P1R+0.5.*P2R+0.5.*P3R
Q2R=35-1.9.*P2R+0.25.*0.5.*P1R+0.5.*P3R
Q3R=35-1.9.*P3R+0.25.*0.5.*P1R+0.5.*P2R
Q1M=40-0.25.*1.8.*P1M+0.5.*P2M+0.5.*P3M
Q2M=35-1.9.*P2M+0.25.*0.5.*P1M+0.5.*P3M
Q3M=35-1.9.*P3M+0.25.*0.5.*P1M+0.5.*P2M
V1R=(P1N-15).*Q1N+0.6.*(P2N-15).*Q2N
V2R=(P1R-15).*Q1R+0.6.*(P2R-15).*Q2R
V3R=(P1M-15).*Q1M+0.6.*(P2M-15).*Q2M
% 映射横坐标到 1-0
theta = 1 - ((o - min(o)) / (max(o) - min(o)));
plot(theta, V1R, '--k', theta, V2R, 'k', theta, V3R, ':k');
xlabel('1-\theta')
ylabel(' V_R');
legend('V_R^N', 'V_R^{RS}', 'V_R^{MS}');
xlim([0 1]);
```

S1 Fig 2. (E) Code

```
o=0.15:0.1:1;
% 计算所有相关变量
A1=[2.*1.9.*0.6.*[40-o.*0.5.*0.6.*15+o.*1.8.*15]+0.5.*(1+o.*0.6).*[35.*0.6-0.5.*15+1.9.*0.6.*15]]./[4.*o.*1.8.*1.9.*0.6-0.5.*0.5.*(1+o.*0.6).*(1+o.*0.6)]
B1=[0.6.*[(1+o.*0.6).*0.5.*0.5+2.*0.5.*1.9.*0.6]]./[4.*o.*1.8.*1.9.*0.6-0.5.*0.5.*(1+o.*0.6).*(1+o.*0.6)]
C1=[35.*0.6-0.5.*15+1.9.*0.6.*15+0.5.*(1+o.*0.6).*A1]./[2.*1.9.*0.6]
D1=[0.5.*(1+o.*0.6).*B1+0.5.*0.6]./[2.*1.9.*0.6]
A2=[0.6.*[2.*1.9.*40+15.*o.*[2.*1.9.*1.8-0.5.*0.5-0.6.*[2.*1.9.*0.5+0.5.*0.5]]]+0.5.*[0.6.*35+15.*0.6]]./[2.*o.*0.6.*[2.*1.9.*1.8-0.5.*0.5]]
```

```
B2=[[1+o.*0.6].*[2.*1.9.*0.5+0.5.*0.5]]./[2.*o.*(2.*1.8.*1.9-0.5.*0.5)]
C2=[0.6.*[2.*1.9.*35+15.*[2.*1.9.*1.9-0.5.*0.5]+0.5.*35]+15.*[0.5.*0.5-
2.*1.9.*0.5]]./[2.*0.6.*[2.*1.9.*1.9-0.5.*0.5]]
D2=[2.*1.9.*0.5+0.5.*0.5+o.*[2.*1.9.*0.5+0.5].*0.6]./[2.*0.6.*(2.*1.9.*1.9-
0.5.*0.5)]
E2=[o.*0.5./(2.*1.9)].*[A2+B2.*C2]./[1-B2.*D2]+0.5.*[C2+[D2.*(A2+B2.*C2)./(1-
B2.*D2)]]./(2.*1.9)
A3=[2.*1.9.*0.6.*[40-o.*0.5.*0.6.*15+o.*1.8.*15]+0.5.*(1+o.*0.6).*[35.*0.6-
0.5.*15+1.9.*0.6.*15]]./[4.*o.*1.8.*1.9.*0.6-0.5.*0.5.*(1+o.*0.6).*(1+o.*0.6)]
B3=[0.6.*[(1+o.*0.6).*0.5.*0.5+2.*0.5.*1.9.*0.6]]./[4.*o.*1.8.*1.9.*0.6-
0.5.*0.5.*(1+o.*0.6).*(1+o.*0.6)]
C3=[35.*0.6-0.5.*15+1.9.*0.6.*15+0.5.*(1+o.*0.6).*A3]./[2.*1.9.*0.6]
D3=[0.5.*(1+o.*0.6).*B3+0.5.*0.6]./[2.*1.9.*0.6]
E3=15.*[(-o.*1.8.*B3+0.5.*D3+0.5)+o.*0.5.*B3-1.9.*D3+0.5]./[2.*0.6.*[1.9-
o.*0.5.*B3-0.5.*D3]]
F3=[35+o.*0.5.*A3+0.5.*C3]./[2.*[1.9-o.*0.5.*B3-0.5.*D3]]
P1N=A1+B1.*[0.6.*[35+o.*0.5.*A1+0.5.*C1]+15.*[0.5+0.5]]./[0.6.*[2.*1.9-
o.*0.5.*B1-0.5.*D1]]
P1R=(A2+B2.*C2)./(1-B2.*D2)
P1M=A3+B3.*[E3+F3]
P2N=C1+D1.*[0.6.*[35+o.*0.5.*A1+0.5.*C1]+15.*[0.5+0.5]]./[0.6.*[2.*1.9-
o.*0.5.*B1-0.5.*D1]]
P2R=C2+[D2.*(A2+B2.*C2)]./(1-B2.*D2)
P2M=C3+D3.*[E3+F3]
P3N=[0.6.*[35+o.*0.5.*A1+0.5.*C1]+15.*[0.5+0.5]]./[0.6.*[2.*1.9-o.*0.5.*B1-
0.5.*D1]]
P3R=E2+[0.6.*35+15.*0.6]./(2.*1.9.*0.6)
P3M=E3+F3
Q1N=40-0.25.*1.8.*P1N+0.5.*P2N+0.5.*P3N
Q2N=35-1.9.*P2N+0.25.*0.5.*P1N+0.5.*P3N
Q3N=35-1.9.*P3N+0.25.*0.5.*P1N+0.5.*P2N
Q1R=40-0.25.*1.8.*P1R+0.5.*P2R+0.5.*P3R
Q2R=35-1.9.*P2R+0.25.*0.5.*P1R+0.5.*P3R
Q3R=35-1.9.*P3R+0.25.*0.5.*P1R+0.5.*P2R
Q1M=40-0.25.*1.8.*P1M+0.5.*P2M+0.5.*P3M
Q2M=35-1.9.*P2M+0.25.*0.5.*P1M+0.5.*P3M
Q3M=35-1.9.*P3M+0.25.*0.5.*P1M+0.5.*P2M
V1M=0.6.*P3N.*Q3N+15.*(Q1N+Q2N)
V2M=0.6.*P3R.*Q3R+15.*(Q1R+Q2R)
V3M=0.6.*P3M.*Q3M+15.*(Q1M+Q2M)
% 映射横坐标到 1-0
theta = 1 - ((o - min(o)) / (max(o) - min(o)));
plot(theta, V1M, '--k', theta, V2M, 'k', theta, V3M, ':k');
xlabel('1-\theta')
ylabel('V_M');
legend('V_M^N', 'V_M^{RS}', 'V_M^{MS}');
xlim([0 1]);
```

S1 Fig 2. (F) Code

```
o=0.17:0.1:1;
% 计算所有相关变量
A1=[2.*1.9.*0.6.*[40-o.*0.5.*0.6.*15+o.*1.8.*15]+0.5.*(1+o.*0.6).*[35.*0.6-
0.5.*15+1.9.*0.6.*15]]./[4.*o.*1.8.*1.9.*0.6-0.5.*0.5.*(1+o.*0.6).*(1+o.*0.6)]
B1=[0.6.*[(1+o.*0.6).*0.5.*0.5+2.*0.5.*1.9.*0.6]]./[4.*o.*1.8.*1.9.*0.6-
0.5.*0.5.*(1+o.*0.6).*(1+o.*0.6)]
C1=[35.*0.6-0.5.*15+1.9.*0.6.*15+0.5.*(1+o.*0.6).*A1]./[2.*1.9.*0.6]
D1=[0.5.*(1+o.*0.6).*B1+0.5.*0.6]./[2.*1.9.*0.6]
A2=[0.6.*[2.*1.9.*40+15.*o.*[2.*1.9.*1.8-0.5.*0.5-
0.6.*[2.*1.9.*0.5+0.5.*0.5]]]+0.5.*[0.6.*35+15.*0.6]]./[2.*o.*0.6.*[2.*1.9.*1.8-
0.5.*0.5]]
B2=[[1+o.*0.6].*[2.*1.9.*0.5+0.5.*0.5]]./[2.*o.*(2.*1.8.*1.9-0.5.*0.5)]
C2=[0.6.*[2.*1.9.*35+15.*[2.*1.9.*1.9-0.5.*0.5]+0.5.*35]+15.*[0.5.*0.5-
2.*1.9.*0.5]]./[2.*0.6.*[2.*1.9.*1.9-0.5.*0.5]]
D2=[2.*1.9.*0.5+0.5.*0.5+o.*[2.*1.9.*0.5+0.5].*0.6]./[2.*0.6.*(2.*1.9.*1.9-
0.5.*0.5)]
E2=[o.*0.5./(2.*1.9)].*[A2+B2.*C2]./[1-B2.*D2]+0.5.*[C2+[D2.*(A2+B2.*C2)./(1-
B2.*D2)]]./(2.*1.9)
A3=[2.*1.9.*0.6.*[40-o.*0.5.*0.6.*15+o.*1.8.*15]+0.5.*(1+o.*0.6).*[35.*0.6-
0.5.*15+1.9.*0.6.*15]]./[4.*o.*1.8.*1.9.*0.6-0.5.*0.5.*(1+o.*0.6).*(1+o.*0.6)]
B3=[0.6.*[(1+o.*0.6).*0.5.*0.5+2.*0.5.*1.9.*0.6]]./[4.*o.*1.8.*1.9.*0.6-
0.5.*0.5.*(1+o.*0.6).*(1+o.*0.6)]
C3=[35.*0.6-0.5.*15+1.9.*0.6.*15+0.5.*(1+o.*0.6).*A3]./[2.*1.9.*0.6]
D3=[0.5.*(1+o.*0.6).*B3+0.5.*0.6]./[2.*1.9.*0.6]
E3=15.*[(-o.*1.8.*B3+0.5.*D3+0.5)+o.*0.5.*B3-1.9.*D3+0.5]./[2.*0.6.*[1.9-
o.*0.5.*B3-0.5.*D3]]
F3=[35+o.*0.5.*A3+0.5.*C3]./[2.*[1.9-o.*0.5.*B3-0.5.*D3]]
P1N=A1+B1.*[0.6.*[35+o.*0.5.*A1+0.5.*C1]+15.*[0.5+0.5]]./[0.6.*[2.*1.9-
o.*0.5.*B1-0.5.*D1]]
P1R=(A2+B2.*C2)./(1-B2.*D2)
P1M=A3+B3.*[E3+F3]
P2N=C1+D1.*[0.6.*[35+o.*0.5.*A1+0.5.*C1]+15.*[0.5+0.5]]./[0.6.*[2.*1.9-
o.*0.5.*B1-0.5.*D1]]
P2R=C2+[D2.*(A2+B2.*C2)]./(1-B2.*D2)
P2M=C3+D3.*[E3+F3]
P3N=[0.6.*[35+o.*0.5.*A1+0.5.*C1]+15.*[0.5+0.5]]./[0.6.*[2.*1.9-o.*0.5.*B1-
0.5.*D1]]
P3R=E2+[0.6.*35+15.*0.6]./(2.*1.9.*0.6)
P3M=E3+F3
Q1N=40-0.25.*1.8.*P1N+0.5.*P2N+0.5.*P3N
Q2N=35-1.9.*P2N+0.25.*0.5.*P1N+0.5.*P3N
Q3N=35-1.9.*P3N+0.25.*0.5.*P1N+0.5.*P2N
Q1R=40-0.25.*1.8.*P1R+0.5.*P2R+0.5.*P3R
Q2R=35-1.9.*P2R+0.25.*0.5.*P1R+0.5.*P3R
Q3R=35-1.9.*P3R+0.25.*0.5.*P1R+0.5.*P2R
Q1M=40-0.25.*1.8.*P1M+0.5.*P2M+0.5.*P3M
Q2M=35-1.9.*P2M+0.25.*0.5.*P1M+0.5.*P3M
Q3M=35-1.9.*P3M+0.25.*0.5.*P1M+0.5.*P2M
V1R=(P1N-15).*Q1N+0.6.*(P2N-15).*Q2N
V2R=(P1R-15).*Q1R+0.6.*(P2R-15).*Q2R
```

```
V3R=(P1M-15).*Q1M+0.6.*(P2M-15).*Q2M
V1M=0.6.*P3N.*Q3N+15.*(Q1N+Q2N)
V2M=0.6.*P3R.*Q3R+15.*(Q1R+Q2R)
V3M=0.6.*P3M.*Q3M+15.*(Q1M+Q2M)
V1=V1R+V1M
V2=V2R+V2M
V3=V3R+V3M
% 归一化横坐标范围为 0 到 1
theta = (1 - o - min(1 - o)) / (max(1 - o) - min(1 - o));
% 绘图
plot(theta, V1, '--k', theta, V2, 'k', theta, V3, ':k');
xlabel('1 - \theta');
ylabel('V');
legend('V^N', 'V^{RS}', 'V^{MS}');
```

S2 Fig 3. (A) Code

```
y1=0:0.1:1;
A1=[2.*(1.4+y1).*0.6.*[40-
0.25.*y1.*0.6.*15+0.25.*(1.3+y1).*15]+y1.*(1+0.25.*0.6).*[35.*0.6-
y1.*15+(1.4+y1).*0.6.*15]]./[4.*0.25.*(1.3+y1).*(1.4+y1).*0.6-
y1.*y1.*(1+0.25.*0.6).*(1+0.25.*0.6)]
B1=[0.6.*[(1+0.25.*0.6).*y1.*0.5+2.*0.5.*(1.4+y1).*0.6]]./[4.*0.25.*(1.3+y1).*(1.4+
y1).*0.6-y1.*y1.*(1+0.25.*0.6).*(1+0.25.*0.6)]
C1=[35.*0.6-y1.*15+(1.4+y1).*0.6.*15+y1.*(1+0.25.*0.6).*A1]./[2.*(1.4+y1).*0.6]
D1=[y1.*(1+0.25.*0.6).*B1+0.5.*0.6]./[2.*(1.4+y1).*0.6]
A2=[0.6.*[2.*1.9.*40+0.25.*15.*[2.*1.9.*(1.3+y1)]-0.5.*0.5-
0.6.*[2.*1.9.*y1+0.5.*0.5]]+0.5.*[0.6.*35+15.*[0.5+0.5]]]./[2.*0.25.*0.6.*( 2.*1.9.*(
1.3+y1)-0.5.*0.5)]
B2=[[1+0.25.*0.6].*[2.*1.9.*y1+0.5.*0.5]]./[2.*0.25.*(2.*(1.3+y1).*1.9-0.5.*0.5)]
C2=[0.6.*[2.*1.9.*35+15.*[2.*1.9.*(1.3+y1)-0.5*0.5]+0.5.*35]+15.*[0.5.*0.5-
2.*1.9.*y1]]./[2.*0.6.*[2.*1.9.*1.9-0.5.*0.5]]
D2=[2.*1.9.*y1+0.5.*0.5+0.25.*[2.*1.9.*y1+0.5].*0.6]./[2.*0.6.*(2.*1.9.*(1.4+y1)-
0.5.*0.5)]
E2=[0.25.*0.5./(2.*1.9)].*[A2+B2.*C2]./[1-
B2.*D2]+0.5.*[C2+[D2.*(A2+B2.*C2)./(1-B2.*D2)]]./(2.*1.9)
A3=[2.*(1.4+y1).*0.6.*[40-
0.25.*y1.*0.6.*15+0.25.*(1.3+y1).*15]+y1.*(1+0.25.*0.6).*[35.*0.6-
y1.*15+(1.4+y1).*0.6.*15]]./[4.*0.25.*(1.3+y1).*(1.4+y1).*0.6-
y1.*y1.*(1+0.25.*0.6).*(1+0.25.*0.6)]
B3=[0.6.*[(1+0.25.*0.6).*y1.*0.5+2.*0.5.*(1.4+y1).*0.6]]./[4.*0.25.*(1.3+y1).*(1.4+
y1).*0.6-y1.*y1.*(1+0.25.*0.6).*(1+0.25.*0.6)]
C3=[35.*0.6-y1.*15+(1.4+y1).*0.6.*15+y1.*(1+0.25.*0.6).*A3]./[2.*(1.4+y1).*0.6]
D3=[y1.*(1+0.25.*0.6).*B3+0.5.*0.6]./[2.*(1.4+y1).*0.6]
E3=15.*[(-0.25.*(1.3+y1).*B3+y1.*D3+0.5)+0.25.*y1.*B3-
(1.4+y1).*D3+0.5]./[2.*0.6.*[1.9-0.25.*0.5.*B3-0.5.*D3]]
F3=[35+0.25.*0.5.*A3+0.5.*C3]./[2.*[1.9-0.25.*0.5.*B3-0.5.*D3]]
P1N=A1+B1.*[0.6.*[35+0.25.*0.5.*A1+0.5.*C1]+15.*[0.5+0.5]]./[0.6.*[2.*1.9-
0.25.*0.5.*B1-0.5.*D1]]
```

```
P1R=(A2+B2.*C2)./(1-B2.*D2)
P1M=A3+B3.*[E3+F3]
plot(y1,P1N,'--k',y1,P1R,'k',y1,P1M,':k');
xlabel('\gamma_1')
ylabel('p_1');
legend('p_1^N', 'p_1^{RS}', 'p_1^{MS}')
```

S2 Fig 3. (B) Code

```
y1=0:0.1:1;
A1=[2.*(1.4+y1).*0.6.*[40-
0.25.*y1.*0.6.*15+0.25.*(1.3+y1).*15]+y1.*(1+0.25.*0.6).*[35.*0.6-
y1.*15+(1.4+y1).*0.6.*15]]./[4.*0.25.*(1.3+y1).*(1.4+y1).*0.6-
y1.*y1.*(1+0.25.*0.6).*(1+0.25.*0.6)]
B1=[0.6.*[(1+0.25.*0.6).*y1.*0.5+2.*0.5.*(1.4+y1).*0.6]]./[4.*0.25.*(1.3+y1).*(1.4+
y1).*0.6-y1.*y1.*(1+0.25.*0.6).*(1+0.25.*0.6)]
C1=[35.*0.6-y1.*15+(1.4+y1).*0.6.*15+y1.*(1+0.25.*0.6).*A1]./[2.*(1.4+y1).*0.6]
D1=[y1.*(1+0.25.*0.6).*B1+0.5.*0.6]./[2.*(1.4+y1).*0.6]
A2=[0.6.*[2.*1.9.*40+0.25.*15.*[2.*1.9.*(1.3+y1)]-0.5.*0.5-
0.6.*[2.*1.9.*y1+0.5.*0.5]]+0.5.*[0.6.*35+15.*[0.5+0.5]]]./[2.*0.25.*0.6.*( 2.*1.9.*(
1.3+y1)-0.5.*0.5)]
B2=[[1+0.25.*0.6].*[2.*1.9.*y1+0.5.*0.5]]./[2.*0.25.*(2.*(1.3+y1).*1.9-0.5.*0.5)]
C2=[0.6.*[2.*1.9.*35+15.*[2.*1.9.*(1.3+y1)-0.5*0.5]+0.5.*35]+15.*[0.5.*0.5-
2.*1.9.*y1]]./[2.*0.6.*[2.*1.9.*1.9-0.5.*0.5]]
D2=[2.*1.9.*y1+0.5.*0.5+0.25.*[2.*1.9.*y1+0.5].*0.6]./[2.*0.6.*(2.*1.9.*(1.4+y1)-
0.5.*0.5)]
E2=[0.25.*0.5./(2.*1.9)].*[A2+B2.*C2]./[1-
B2.*D2]+0.5.*[C2+[D2.*(A2+B2.*C2)./(1-B2.*D2)]]./(2.*1.9)
A3=[2.*(1.4+y1).*0.6.*[40-
0.25.*y1.*0.6.*15+0.25.*(1.3+y1).*15]+y1.*(1+0.25.*0.6).*[35.*0.6-
y1.*15+(1.4+y1).*0.6.*15]]./[4.*0.25.*(1.3+y1).*(1.4+y1).*0.6-
y1.*y1.*(1+0.25.*0.6).*(1+0.25.*0.6)]
B3=[0.6.*[(1+0.25.*0.6).*y1.*0.5+2.*0.5.*(1.4+y1).*0.6]]./[4.*0.25.*(1.3+y1).*(1.4+
y1).*0.6-y1.*y1.*(1+0.25.*0.6).*(1+0.25.*0.6)]
C3=[35.*0.6-y1.*15+(1.4+y1).*0.6.*15+y1.*(1+0.25.*0.6).*A3]./[2.*(1.4+y1).*0.6]
D3=[y1.*(1+0.25.*0.6).*B3+0.5.*0.6]./[2.*(1.4+y1).*0.6]
E3=15.*[(-0.25.*(1.3+y1).*B3+y1.*D3+0.5)+0.25.*y1.*B3-
(1.4+y1).*D3+0.5]./[2.*0.6.*[1.9-0.25.*0.5.*B3-0.5.*D3]]
F3=[35+0.25.*0.5.*A3+0.5.*C3]./[2.*[1.9-0.25.*0.5.*B3-0.5.*D3]]
P2M=C3+D3.*[E3+F3]
P2R=C2+[D2.*(A2+B2.*C2)]./(1-B2.*D2)
P2N=C1+D1.*[0.6.*[35+0.25.*0.5.*A1+0.5.*C1]+15.*[0.5+0.5]]./[0.6.*[2.*1.9-
0.25.*0.5.*B1-0.5.*D1]]
plot(y1,P2N,'k',y1,P2R,'k',y1,P2M,':k');
xlabel('\gamma_1')
ylabel('p_2');
legend('p_2^N', 'p_2^{RS}', 'p_2^{MS}')
```

S2 Fig 3. (C) Code

```
y1=0:0.1:1;
A1=[2.*(1.4+y1).*0.6.*[40-
0.25.*y1.*0.6.*15+0.25.*(1.3+y1).*15]+y1.*(1+0.25.*0.6).*[35.*0.6-
y1.*15+(1.4+y1).*0.6.*15]]./[4.*0.25.*(1.3+y1).*(1.4+y1).*0.6-
y1.*y1.*(1+0.25.*0.6).*(1+0.25.*0.6)]
B1=[0.6.*[(1+0.25.*0.6).*y1.*0.5+2.*0.5.*(1.4+y1).*0.6]]./[4.*0.25.*(1.3+y1).*(1.4+
y1).*0.6-y1.*y1.*(1+0.25.*0.6).*(1+0.25.*0.6)]
C1=[35.*0.6-y1.*15+(1.4+y1).*0.6.*15+y1.*(1+0.25.*0.6).*A1]./[2.*(1.4+y1).*0.6]
D1=[y1.*(1+0.25.*0.6).*B1+0.5.*0.6]./[2.*(1.4+y1).*0.6]
A2=[0.6.*[2.*1.9.*40+0.25.*15.*[2.*1.9.*(1.3+y1)]-0.5.*0.5-
0.6.*[2.*1.9.*y1+0.5.*0.5]]+0.5.*[0.6.*35+15.*[0.5+0.5]]]./[2.*0.25.*0.6.*( 2.*1.9.*(
1.3+y1)-0.5.*0.5)]
B2=[[1+0.25.*0.6].*[2.*1.9.*y1+0.5.*0.5]]./[2.*0.25.*(2.*(1.3+y1).*1.9-0.5.*0.5)]
C2=[0.6.*[2.*1.9.*35+15.*[2.*1.9.*(1.3+y1)-0.5*0.5]+0.5.*35]+15.*[0.5.*0.5-
2.*1.9.*y1]]./[2.*0.6.*[2.*1.9.*1.9-0.5.*0.5]]
D2=[2.*1.9.*y1+0.5.*0.5+0.25.*[2.*1.9.*y1+0.5].*0.6]./[2.*0.6.*(2.*1.9.*(1.4+y1)-
0.5.*0.5)]
E2=[0.25.*0.5./(2.*1.9)].*[A2+B2.*C2]./[1-
B2.*D2]+0.5.*[C2+[D2.*(A2+B2.*C2)./(1-B2.*D2)]]./(2.*1.9)
A3=[2.*(1.4+y1).*0.6.*[40-
0.25.*y1.*0.6.*15+0.25.*(1.3+y1).*15]+y1.*(1+0.25.*0.6).*[35.*0.6-
y1.*15+(1.4+y1).*0.6.*15]]./[4.*0.25.*(1.3+y1).*(1.4+y1).*0.6-
y1.*y1.*(1+0.25.*0.6).*(1+0.25.*0.6)]
B3=[0.6.*[(1+0.25.*0.6).*y1.*0.5+2.*0.5.*(1.4+y1).*0.6]]./[4.*0.25.*(1.3+y1).*(1.4+
y1).*0.6-y1.*y1.*(1+0.25.*0.6).*(1+0.25.*0.6)]
C3=[35.*0.6-y1.*15+(1.4+y1).*0.6.*15+y1.*(1+0.25.*0.6).*A3]./[2.*(1.4+y1).*0.6]
D3=[y1.*(1+0.25.*0.6).*B3+0.5.*0.6]./[2.*(1.4+y1).*0.6]
E3=15.*[(-0.25.*(1.3+y1).*B3+y1.*D3+0.5)+0.25.*y1.*B3-
(1.4+y1).*D3+0.5]./[2.*0.6.*[1.9-0.25.*0.5.*B3-0.5.*D3]]
F3=[35+0.25.*0.5.*A3+0.5.*C3]./[2.*[1.9-0.25.*0.5.*B3-0.5.*D3]]
P3N=[0.6.*[35+0.25.*0.5.*A1+0.5.*C1]+15.*[0.5+0.5]]./[0.6.*[2.*1.9-
0.25.*0.5.*B1-0.5.*D1]]
P3R=E2+[0.6.*35+15.*[0.5+0.5]]./(2.*1.9.*0.6)
P3M=E3 +F3
plot(y1,P3N,'--k',y1,P3R,'k',y1,P3M,':k');
xlabel('\gamma_1')
ylabel('p_3');
legend('p_3^N', 'p_3^{RS}', 'p_3^{MS}')
```

S2 Fig 3. (D) Code

```
y1=0:0.1:1;
A1=[2.*(1.4+y1).*0.6.*[40-
0.25.*y1.*0.6.*15+0.25.*(1.3+y1).*15]+y1.*(1+0.25.*0.6).*[35.*0.6-
y1.*15+(1.4+y1).*0.6.*15]]./[4.*0.25.*(1.3+y1).*(1.4+y1).*0.6-
y1.*y1.*(1+0.25.*0.6).*(1+0.25.*0.6)]
B1=[0.6.*[(1+0.25.*0.6).*y1.*0.5+2.*0.5.*(1.4+y1).*0.6]]./[4.*0.25.*(1.3+y1).*(1.4+
```

```
y1).*0.6-y1.*y1.*(1+0.25.*0.6).*(1+0.25.*0.6)]
C1=[35.*0.6-y1.*15+(1.4+y1).*0.6.*15+y1.*(1+0.25.*0.6).*A1]./[2.*(1.4+y1).*0.6]
D1=[y1.*(1+0.25.*0.6).*B1+0.5.*0.6]./[2.*(1.4+y1).*0.6]
A2=[0.6.*[2.*1.9.*40+0.25.*15.*[2.*1.9.*(1.3+y1)]-0.5.*0.5-
0.6.*[2.*1.9.*y1+0.5.*0.5]]+0.5.*[0.6.*35+15.*(0.5+0.5)]]./[2.*0.25.*0.6.*( 2.*1.9.*(
1.3+y1)-0.5.*0.5)]
B2=[[1+0.25.*0.6].*[2.*1.9.*y1+0.5.*0.5]]./[2.*0.25.*(2.*(1.3+y1).*1.9-0.5.*0.5)]
C2=[0.6.*[2.*1.9.*35+15.*[2.*1.9.*(1.3+y1)-0.5*0.5]+0.5.*35]+15.*[0.5.*0.5-
2.*1.9.*y1]]./[2.*0.6.*[2.*1.9.*1.9-0.5.*0.5]]
D2=[2.*1.9.*y1+0.5.*0.5+0.25.*[2.*1.9.*y1+0.5].*0.6]./[2.*0.6.*(2.*1.9.*(1.4+y1)-
0.5.*0.5)]
E2=[0.25.*0.5./(2.*1.9)].*[A2+B2.*C2]./[1-
B2.*D2]+0.5.*[C2+[D2.*(A2+B2.*C2)./(1-B2.*D2)]]./(2.*1.9)
A3=[2.*(1.4+y1).*0.6.*[40-
0.25.*y1.*0.6.*15+0.25.*(1.3+y1).*15]+y1.*(1+0.25.*0.6).*[35.*0.6-
y1.*15+(1.4+y1).*0.6.*15]]./[4.*0.25.*(1.3+y1).*(1.4+y1).*0.6-
y1.*y1.*(1+0.25.*0.6).*(1+0.25.*0.6)]
B3=[0.6.*[(1+0.25.*0.6).*y1.*0.5+2.*0.5.*(1.4+y1).*0.6]]./[4.*0.25.*(1.3+y1).*(1.4+
y1).*0.6-y1.*y1.*(1+0.25.*0.6).*(1+0.25.*0.6)]
C3=[35.*0.6-y1.*15+(1.4+y1).*0.6.*15+y1.*(1+0.25.*0.6).*A3]./[2.*(1.4+y1).*0.6]
D3=[y1.*(1+0.25.*0.6).*B3+0.5.*0.6]./[2.*(1.4+y1).*0.6]
E3=15.*[(-0.25.*(1.3+y1).*B3+y1.*D3+0.5)+0.25.*y1.*B3-
(1.4+y1).*D3+0.5]./[2.*0.6.*[1.9-0.25.*0.5.*B3-0.5.*D3]]
F3=[35+0.25.*0.5.*A3+0.5.*C3]./[2.*[1.9-0.25.*0.5.*B3-0.5.*D3]]
P1N=A1+B1.*[0.6.*[35+0.25.*0.5.*A1+0.5.*C1]+15.*[0.5+0.5]]./[0.6.*[2.*1.9-
0.25.*0.5.*B1-0.5.*D1]]
P1R=(A2+B2.*C2)./(1-B2.*D2)
P1M=A3+B3.*[E3+F3]
P2M=C3+D3.*[E3+F3]
P2R=C2+[D2.*(A2+B2.*C2)]./(1-B2.*D2)
P2N=C1+D1.*[0.6.*[35+0.25.*0.5.*A1+0.5.*C1]+15.*[0.5+0.5]]./[0.6.*[2.*1.9-
0.25.*0.5.*B1-0.5.*D1]]
P3N=[0.6.*[35+0.25.*0.5.*A1+0.5.*C1]+15.*[0.5+0.5]]./[0.6.*[2.*1.9-
0.25.*0.5.*B1-0.5.*D1]]
P3R=E2+[0.6.*35+15.*(0.5+0.5)]./(2.*1.9.*0.6)
P3M=E3 +F3
Q1N=40-0.25.*(1.3+y1).*P1N+y1.*P2N+0.5.*P3N
Q2N=35-(1.4+y1).*P2N+0.25.*y1.*P1N+0.5.*P3N
Q3N=35-1.9.*P3N+0.25.*0.5.*P1N+0.5.*P2N
Q1R=40-0.25.*(1.3+y1).*P1R+y1.*P2R+0.5.*P3R
Q2R=35-(1.4+y1).*P2R+0.25.*y1.*P1R+0.5.*P3R
Q3R=35-1.9.*P3R+0.25.*0.5.*P1R+0.5.*P2R
Q1M=40-0.25.*(1.3+y1).*P1M+y1.*P2M+0.5.*P3M
Q2M=35-(1.4+y1).*P2M+0.25.*y1.*P1M+0.5.*P3M
Q3M=35-1.9.*P3M+0.25.*0.5.*P1M+0.5.*P2M
V1R=(P1N-15).*Q1N+0.6.*(P2N-15).*Q2N
V2R=(P1R-15).*Q1R+0.6.*(P2R-15).*Q2R
V3R=(P1M-15).*Q1M+0.6.*(P2M-15).*Q2M
plot(y1,V1R,'--k',y1,V2R,'k',y1,V3R,':k');
xlabel('\gamma_1')
```

```
ylabel(' V_R');
legend('V_R^N', 'V_R^{RS}', 'V_R^{MS}')
```

S2 Fig 3. (E) Code

```
y1=0:0.1:1;
A1=[2.*(1.4+y1).*0.6.*[40-
0.25.*y1.*0.6.*15+0.25.*(1.3+y1).*15]+y1.*(1+0.25.*0.6).*[35.*0.6-
y1.*15+(1.4+y1).*0.6.*15]]./[4.*0.25.*(1.3+y1).*(1.4+y1).*0.6-
y1.*y1.*(1+0.25.*0.6).*(1+0.25.*0.6)]
B1=[0.6.*[(1+0.25.*0.6).*y1.*0.5+2.*0.5.*(1.4+y1).*0.6]]./[4.*0.25.*(1.3+y1).*(1.4+
y1).*0.6-y1.*y1.*(1+0.25.*0.6).*(1+0.25.*0.6)]
C1=[35.*0.6-y1.*15+(1.4+y1).*0.6.*15+y1.*(1+0.25.*0.6).*A1]./[2.*(1.4+y1).*0.6]
D1=[y1.*(1+0.25.*0.6).*B1+0.5.*0.6]./[2.*(1.4+y1).*0.6]
A2=[0.6.*[2.*1.9.*40+0.25.*15.*[2.*1.9.*(1.3+y1)]-0.5.*0.5-
0.6.*[2.*1.9.*y1+0.5.*0.5]]+0.5.*[0.6.*35+15.*(0.5+0.5)]]./[2.*0.25.*0.6.*( 2.*1.9.*(
1.3+y1)-0.5.*0.5)]
B2=[[1+0.25.*0.6].*[2.*1.9.*y1+0.5.*0.5]]./[2.*0.25.*(2.*(1.3+y1).*1.9-0.5.*0.5)]
C2=[0.6.*[2.*1.9.*35+15.*[2.*1.9.*(1.3+y1)-0.5*0.5]+0.5.*35]+15.*[0.5.*0.5-
2.*1.9.*y1]]./[2.*0.6.*[2.*1.9.*1.9-0.5.*0.5]]
D2=[2.*1.9.*y1+0.5.*0.5+0.25.*[2.*1.9.*y1+0.5].*0.6]./[2.*0.6.*(2.*1.9.*(1.4+y1)-
0.5.*0.5)]
E2=[0.25.*0.5./(2.*1.9)].*[A2+B2.*C2]./[1-
B2.*D2]+0.5.*[C2+[D2.*(A2+B2.*C2)./(1-B2.*D2)]]./(2.*1.9)
A3=[2.*(1.4+y1).*0.6.*[40-
0.25.*y1.*0.6.*15+0.25.*(1.3+y1).*15]+y1.*(1+0.25.*0.6).*[35.*0.6-
y1.*15+(1.4+y1).*0.6.*15]]./[4.*0.25.*(1.3+y1).*(1.4+y1).*0.6-
y1.*y1.*(1+0.25.*0.6).*(1+0.25.*0.6)]
B3=[0.6.*[(1+0.25.*0.6).*y1.*0.5+2.*0.5.*(1.4+y1).*0.6]]./[4.*0.25.*(1.3+y1).*(1.4+
y1).*0.6-y1.*y1.*(1+0.25.*0.6).*(1+0.25.*0.6)]
C3=[35.*0.6-y1.*15+(1.4+y1).*0.6.*15+y1.*(1+0.25.*0.6).*A3]./[2.*(1.4+y1).*0.6]
D3=[y1.*(1+0.25.*0.6).*B3+0.5.*0.6]./[2.*(1.4+y1).*0.6]
E3=15.*[(-0.25.*(1.3+y1).*B3+y1.*D3+0.5)+0.25.*y1.*B3-
(1.4+y1).*D3+0.5]./[2.*0.6.*[1.9-0.25.*0.5.*B3-0.5.*D3]]
F3=[35+0.25.*0.5.*A3+0.5.*C3]./[2.*[1.9-0.25.*0.5.*B3-0.5.*D3]]
P1N=A1+B1.*[0.6.*[35+0.25.*0.5.*A1+0.5.*C1]+15.*[0.5+0.5]]./[0.6.*[2.*1.9-
0.25.*0.5.*B1-0.5.*D1]]
P1R=(A2+B2.*C2)./(1-B2.*D2)
P1M=A3+B3.*[E3+F3]
P2M=C3+D3.*[E3+F3]
P2R=C2+[D2.*(A2+B2.*C2)]./(1-B2.*D2)
P2N=C1+D1.*[0.6.*[35+0.25.*0.5.*A1+0.5.*C1]+15.*[0.5+0.5]]./[0.6.*[2.*1.9-
0.25.*0.5.*B1-0.5.*D1]]
P3N=[0.6.*[35+0.25.*0.5.*A1+0.5.*C1]+15.*[0.5+0.5]]./[0.6.*[2.*1.9-
0.25.*0.5.*B1-0.5.*D1]]
P3R=E2+[0.6.*35+15.*(0.5+0.5)]./(2.*1.9.*0.6)
P3M=E3 +F3
Q1N=40-0.25.*(1.3+y1).*P1N+y1.*P2N+0.5.*P3N
Q2N=35-(1.4+y1).*P2N+0.25.*y1.*P1N+0.5.*P3N
```

```
Q3N=35-1.9.*P3N+0.25.*0.5.*P1N+0.5.*P2N
Q1R=40-0.25.*(1.3+y1).*P1R+y1.*P2R+0.5.*P3R
Q2R=35-(1.4+y1).*P2R+0.25.*y1.*P1R+0.5.*P3R
Q3R=35-1.9.*P3R+0.25.*0.5.*P1R+0.5.*P2R
Q1M=40-0.25.*(1.3+y1).*P1M+y1.*P2M+0.5.*P3M
Q2M=35-(1.4+y1).*P2M+0.25.*y1.*P1M+0.5.*P3M
Q3M=35-1.9.*P3M+0.25.*0.5.*P1M+0.5.*P2M
V1M=0.6.*P3N.*Q3N+15.*(Q1N+Q2N)
V2M=0.6.*P3R.*Q3R+15.*(Q1R+Q2R)
V3M=0.6.*P3M.*Q3M+15.*(Q1M+Q2M)
plot(y1,V1M,'k',y1,V2M,'k',y1,V3M,':k');
xlabel('\gamma_1')
ylabel(' V_M');
legend('V_M^N', 'V_M^{RS}', 'V_M^{MS}')
```

S2 Fig 3. (F) Code

```
y1=0:0.1:1;
A1=[2.*(1.4+y1).*0.6.*[40-
0.25.*y1.*0.6.*15+0.25.*(1.3+y1).*15]+y1.*(1+0.25.*0.6).*[35.*0.6-
y1.*15+(1.4+y1).*0.6.*15]]./[4.*0.25.*(1.3+y1).*(1.4+y1).*0.6-
y1.*y1.*(1+0.25.*0.6).*(1+0.25.*0.6)]
B1=[0.6.*[(1+0.25.*0.6).*y1.*0.5+2.*0.5.*(1.4+y1).*0.6]]./[4.*0.25.*(1.3+y1).*(1.4+
y1).*0.6-y1.*y1.*(1+0.25.*0.6).*(1+0.25.*0.6)]
C1=[35.*0.6-y1.*15+(1.4+y1).*0.6.*15+y1.*(1+0.25.*0.6).*A1]./[2.*(1.4+y1).*0.6]
D1=[y1.*(1+0.25.*0.6).*B1+0.5.*0.6]./[2.*(1.4+y1).*0.6]
A2=[0.6.*[2.*1.9.*40+0.25.*15.*[2.*1.9.*(1.3+y1)]-0.5.*0.5-
0.6.*[2.*1.9.*y1+0.5.*0.5]]+0.5.*[0.6.*35+15.*(0.5+0.5)]]./[2.*0.25.*0.6.*( 2.*1.9.*(
1.3+y1)-0.5.*0.5)]
B2=[[1+0.25.*0.6].*[2.*1.9.*y1+0.5.*0.5]]./[2.*0.25.*(2.*(1.3+y1).*1.9-0.5.*0.5)]
C2=[0.6.*[2.*1.9.*35+15.*[2.*1.9.*(1.3+y1)-0.5*0.5]+0.5.*35]+15.*[0.5.*0.5-
2.*1.9.*y1]]./[2.*0.6.*[2.*1.9.*1.9-0.5.*0.5]]
D2=[2.*1.9.*y1+0.5.*0.5+0.25.*[2.*1.9.*y1+0.5].*0.6]./[2.*0.6.*(2.*1.9.*(1.4+y1)-
0.5.*0.5)]
E2=[0.25.*0.5./(2.*1.9)].*[A2+B2.*C2]./[1-
B2.*D2]+0.5.*[C2+[D2.*(A2+B2.*C2)./(1-B2.*D2)]]./(2.*1.9)
A3=[2.*(1.4+y1).*0.6.*[40-
0.25.*y1.*0.6.*15+0.25.*(1.3+y1).*15]+y1.*(1+0.25.*0.6).*[35.*0.6-
y1.*15+(1.4+y1).*0.6.*15]]./[4.*0.25.*(1.3+y1).*(1.4+y1).*0.6-
y1.*y1.*(1+0.25.*0.6).*(1+0.25.*0.6)]
B3=[0.6.*[(1+0.25.*0.6).*y1.*0.5+2.*0.5.*(1.4+y1).*0.6]]./[4.*0.25.*(1.3+y1).*(1.4+
y1).*0.6-y1.*y1.*(1+0.25.*0.6).*(1+0.25.*0.6)]
C3=[35.*0.6-y1.*15+(1.4+y1).*0.6.*15+y1.*(1+0.25.*0.6).*A3]./[2.*(1.4+y1).*0.6]
D3=[y1.*(1+0.25.*0.6).*B3+0.5.*0.6]./[2.*(1.4+y1).*0.6]
E3=15.*[(-0.25.*(1.3+y1).*B3+y1.*D3+0.5)+0.25.*y1.*B3-
(1.4+y1).*D3+0.5]./[2.*0.6.*[1.9-0.25.*0.5.*B3-0.5.*D3]]
F3=[35+0.25.*0.5.*A3+0.5.*C3]./[2.*[1.9-0.25.*0.5.*B3-0.5.*D3]]
P1N=A1+B1.*[0.6.*[35+0.25.*0.5.*A1+0.5.*C1]+15.*[0.5+0.5]]./[0.6.*[2.*1.9-
0.25.*0.5.*B1-0.5.*D1]]
```

```
P1R=(A2+B2.*C2)./(1-B2.*D2)
P1M=A3+B3.*[E3+F3]
P2M=C3+D3.*[E3+F3]
P2R=C2+[D2.*(A2+B2.*C2)]./(1-B2.*D2)
P2N=C1+D1.*[0.6.*[35+0.25.*0.5.*A1+0.5.*C1]+15.*[0.5+0.5]]./[0.6.*[2.*1.9-
0.25.*0.5.*B1-0.5.*D1]]
P3N=[0.6.*[35+0.25.*0.5.*A1+0.5.*C1]+15.*[0.5+0.5]]./[0.6.*[2.*1.9-
0.25.*0.5.*B1-0.5.*D1]]
P3R=E2+[0.6.*35+15.*(0.5+0.5)]./(2.*1.9.*0.6)
P3M=E3 +F3
Q1N=40-0.25.*(1.3+y1).*P1N+y1.*P2N+0.5.*P3N
Q2N=35-(1.4+y1).*P2N+0.25.*y1.*P1N+0.5.*P3N
Q3N=35-1.9.*P3N+0.25.*0.5.*P1N+0.5.*P2N
Q1R=40-0.25.*(1.3+y1).*P1R+y1.*P2R+0.5.*P3R
Q2R=35-(1.4+y1).*P2R+0.25.*y1.*P1R+0.5.*P3R
Q3R=35-1.9.*P3R+0.25.*0.5.*P1R+0.5.*P2R
Q1M=40-0.25.*(1.3+y1).*P1M+y1.*P2M+0.5.*P3M
Q2M=35-(1.4+y1).*P2M+0.25.*y1.*P1M+0.5.*P3M
Q3M=35-1.9.*P3M+0.25.*0.5.*P1M+0.5.*P2M
V1R=(P1N-15).*Q1N+0.6.*(P2N-15).*Q2N
V2R=(P1R-15).*Q1R+0.6.*(P2R-15).*Q2R
V3R=(P1M-15).*Q1M+0.6.*(P2M-15).*Q2M
V1M=0.6.*P3N.*Q3N+15.*(Q1N+Q2N)
V2M=0.6.*P3R.*Q3R+15.*(Q1R+Q2R)
V3M=0.6.*P3M.*Q3M+15.*(Q1M+Q2M)
V1=V1R+V1M
V2=V2R+V2M
V3=V3R+V3M
plot(y1,V1,'--k',y1,V2,'k',y1,V3,':k');
xlabel('\gamma_1')
ylabel('V');
legend('V^N', 'V^{RS}', 'V^{MS}')
```

S3 Fig 4. (A) Code

```
y2=0:0.1:1;
A1=[2.*1.9.*0.6.*[40-
0.25.*0.5.*0.6.*15+0.25.*(1.3+y2).*15]+0.5.*(1+0.25.*0.6).*[35.*0.6-
0.5.*15+1.9.*0.6.*15]]./[4.*0.25.*(1.3+y2).*1.9.*0.6-
0.5.*0.5.*(1+0.25.*0.6).*(1+0.25.*0.6)]
B1=[0.6.*[(1+0.25.*0.6).*0.5.*0.5+2.*y2.*1.9.*0.6]]./[4.*0.25.*(1.3+y2).*1.9.*0.6-
0.5.*0.5.*(1+0.25.*0.6).*(1+0.25.*0.6)]
C1=[35.*0.6-0.5.*15+1.9.*0.6.*15+0.5.*(1+0.25.*0.6).*A1]./[2.*1.9.*0.6]
D1=[0.5.*(1+0.25.*0.6).*B1+0.5.*0.6]./[2.*1.9.*0.6]
A2=[0.6.*[2.*(1.4+y2).*40+15.*0.25.*[2.*(1.4+y2).*(1.3+y2)-y2.*y2-
0.6.*[2.*(1.4+y2).*0.5+y2.*0.5]]]+y2.*[0.6.*35+15.*(y2+0.5)]]./[2.*0.25.*0.6.*[2.*(
1.4+y2).*(1.3+y2)-y2.*y2]]
B2=[[1+0.25.*0.6].*[2.*(1.4+y2).*0.5+y2.*0.5]]./[2.*0.25.*(2.*(1.3+y2).*(1.4+y2)-
y2.*y2)]
```

```
C2=[0.6.*[2.*(1.4+y2).*35+15.*[2.*(1.4+y2).*1.9-0.5.*0.5]+0.5.*35]+15.*[0.5.*0.5-
2.*(1.4+y2).*0.5]]./[2.*0.6.*[2.*(1.4+y2).*1.9-0.5.*0.5]]
D2=[2.*(1.4+y2).*0.5+y2.*0.5+0.25.*[2.*(1.4+y2).*0.5+y2].*0.6]./[2.*0.6.*(2.*(1.4
+y2).*1.9-0.5.*0.5)]
E2=[0.25.*y2./(2.*(1.4+y2))].*[A2+B2.*C2]./[1-
B2.*D2]+0.5.*[C2+[D2.*(A2+B2.*C2)./(1-B2.*D2)]]./(2.*(1.4+y2))
A3=[2.*1.9.*0.6.*[40-
0.25.*0.5.*0.6.*15+0.25.*(1.3+y2).*15]+0.5.*(1+0.25.*0.6).*[35.*0.6-
0.5.*15+1.9.*0.6.*15]]./[4.*0.25.*(1.3+y2).*1.9.*0.6-
0.5.*0.5.*(1+0.25.*0.6).*(1+0.25.*0.6)]
B3=[0.6.*[(1+0.25.*0.6).*0.5.*0.5+2.*y2.*1.9.*0.6]]./[4.*0.25.*(1.3+y2).*1.9.*0.6-
0.5.*0.5.*(1+0.25.*0.6).*(1+0.25.*0.6)]
C3=[35.*0.6-0.5.*15+1.9.*0.6.*15+0.5.*(1+0.25.*0.6).*A3]./[2.*1.9.*0.6]
D3=[0.5.*(1+0.25.*0.6).*B3+0.5.*0.6]./[2.*1.9.*0.6]
E3=15.*[-0.25.*(1.3+y2).*B3+0.5.*D3+y2+0.25.*0.5.*B3-
1.9.*D3+0.5]./[2.*0.6.*[(1.4+y2)-0.25.*y2.*B3-0.5.*D3]]
F3=[35+0.25.*y2.*A3+0.5.*C3]./[2.*[1.4+y2-0.25.*y2.*B3-0.5.*D3]]
P1N=A1+B1.*[0.6.*[35+0.25.*y2.*A1+0.5.*C1]+15.*[y2+0.5]]./[0.6.*[2.*(1.4+y2)-
0.25.*y2.*B1-0.5.*D1]]
P1R=(A2+B2.*C2)./(1-B2.*D2)
P1M=A3+B3.*[E3+F3]
plot(y2,P1N,'--k',y2,P1R,'k',y2,P1M,':k');
xlabel('\gamma_2')
ylabel('p_1');
legend('p_1^N', 'p_1^{RS}', 'p_1^{MS}')
```

S3 Fig 4. (B) Code

```
y2=0:0.1:1;
A1=[2.*1.9.*0.6.*[40-
0.25.*0.5.*0.6.*15+0.25.*(1.3+y2).*15]+0.5.*(1+0.25.*0.6).*[35.*0.6-
0.5.*15+1.9.*0.6.*15]]./[4.*0.25.*(1.3+y2).*1.9.*0.6-
0.5.*0.5.*(1+0.25.*0.6).*(1+0.25.*0.6)]
B1=[0.6.*[(1+0.25.*0.6).*0.5.*0.5+2.*y2.*1.9.*0.6]]./[4.*0.25.*(1.3+y2).*1.9.*0.6-
0.5.*0.5.*(1+0.25.*0.6).*(1+0.25.*0.6)]
C1=[35.*0.6-0.5.*15+1.9.*0.6.*15+0.5.*(1+0.25.*0.6).*A1]./[2.*1.9.*0.6]
D1=[0.5.*(1+0.25.*0.6).*B1+0.5.*0.6]./[2.*1.9.*0.6]
A2=[0.6.*[2.*(1.4+y2).*40+15.*0.25.*[2.*(1.4+y2).*(1.3+y2)-y2.*y2-
0.6.*[2.*(1.4+y2).*0.5+y2.*0.5]]]+y2.*[0.6.*35+15.*(y2+0.5)]]./[2.*0.25.*0.6.*[2.*(
1.4+y2).*(1.3+y2)-y2.*y2]]
B2=[[1+0.25.*0.6].*[2.*(1.4+y2).*0.5+y2.*0.5]]./[2.*0.25.*(2.*(1.3+y2).*(1.4+y2)-
y2.*y2)]
C2=[0.6.*[2.*(1.4+y2).*35+15.*[2.*(1.4+y2).*1.9-0.5.*0.5]+0.5.*35]+15.*[0.5.*0.5-
2.*(1.4+y2).*0.5]]./[2.*0.6.*[2.*(1.4+y2).*1.9-0.5.*0.5]]
D2=[2.*(1.4+y2).*0.5+y2.*0.5+0.25.*[2.*(1.4+y2).*0.5+y2].*0.6]./[2.*0.6.*(2.*(1.4
+y2).*1.9-0.5.*0.5)]
E2=[0.25.*y2./(2.*(1.4+y2))].*[A2+B2.*C2]./[1-
B2.*D2]+0.5.*[C2+[D2.*(A2+B2.*C2)./(1-B2.*D2)]]./(2.*(1.4+y2))
A3=[2.*1.9.*0.6.*[40-
```

```
0.25.*0.5.*0.6.*15+0.25.*(1.3+y2).*15]+0.5.*(1+0.25.*0.6).*[35.*0.6-
0.5.*15+1.9.*0.6.*15]]./[4.*0.25.*(1.3+y2).*1.9.*0.6-
0.5.*0.5.*(1+0.25.*0.6).*(1+0.25.*0.6)]
B3=[0.6.*[(1+0.25.*0.6).*0.5.*0.5+2.*y2.*1.9.*0.6]]./[4.*0.25.*(1.3+y2).*1.9.*0.6-
0.5.*0.5.*(1+0.25.*0.6).*(1+0.25.*0.6)]
C3=[35.*0.6-0.5.*15+1.9.*0.6.*15+0.5.*(1+0.25.*0.6).*A3]./[2.*1.9.*0.6]
D3=[0.5.*(1+0.25.*0.6).*B3+0.5.*0.6]./[2.*1.9.*0.6]
E3=15.*[-0.25.*(1.3+y2).*B3+0.5.*D3+y2+0.25.*0.5.*B3-
1.9.*D3+0.5]./[2.*0.6.*[(1.4+y2)-0.25.*y2.*B3-0.5.*D3]]
F3=[35+0.25.*y2.*A3+0.5.*C3]./[2.*[1.4+y2-0.25.*y2.*B3-0.5.*D3]]
P2M=C3+D3.*[E3+F3]
P2R=C2+[D2.*(A2+B2.*C2)]./(1-B2.*D2)
P2N=C1+D1.*[0.6.*[35+0.25.*y2.*A1+0.5.*C1]+15.*[y2+0.5]]./[0.6.*[2.*(1.4+y2)-
0.25.*y2.*B1-0.5.*D1]]
plot(y2,P2N,'--k',y2,P2R,'k',y2,P2M,':k');
xlabel('\gamma_2')
ylabel('p_2');
legend('p_2^N', 'p_2^{RS}', 'p_2^{MS}')
```

S3 Fig 4. (C) Code

```
y2=0:0.1:1;
A1=[2.*1.9.*0.6.*[40-
0.25.*0.5.*0.6.*15+0.25.*(1.3+y2).*15]+0.5.*(1+0.25.*0.6).*[35.*0.6-
0.5.*15+1.9.*0.6.*15]]./[4.*0.25.*(1.3+y2).*1.9.*0.6-
0.5.*0.5.*(1+0.25.*0.6).*(1+0.25.*0.6)]
B1=[0.6.*[(1+0.25.*0.6).*0.5.*0.5+2.*y2.*1.9.*0.6]]./[4.*0.25.*(1.3+y2).*1.9.*0.6-
0.5.*0.5.*(1+0.25.*0.6).*(1+0.25.*0.6)]
C1=[35.*0.6-0.5.*15+1.9.*0.6.*15+0.5.*(1+0.25.*0.6).*A1]./[2.*1.9.*0.6]
D1=[0.5.*(1+0.25.*0.6).*B1+0.5.*0.6]./[2.*1.9.*0.6]
A2=[0.6.*[2.*(1.4+y2).*40+15.*0.25.*[2.*(1.4+y2).*(1.3+y2)-y2.*y2-
0.6.*[2.*(1.4+y2).*0.5+y2.*0.5]]]+y2.*[0.6.*35+15.*(y2+0.5)]]./[2.*0.25.*0.6.*[2.*(
1.4+y2).*(1.3+y2)-y2.*y2]]
B2=[[1+0.25.*0.6].*[2.*(1.4+y2).*0.5+y2.*0.5]]./[2.*0.25.*(2.*(1.3+y2).*(1.4+y2)-
y2.*y2)]
C2=[0.6.*[2.*(1.4+y2).*35+15.*[2.*(1.4+y2).*1.9-0.5.*0.5]+0.5.*35]+15.*[0.5.*0.5-
2.*(1.4+y2).*0.5]]./[2.*0.6.*[2.*(1.4+y2).*1.9-0.5.*0.5]]
D2=[2.*(1.4+y2).*0.5+y2.*0.5+0.25.*[2.*(1.4+y2).*0.5+y2].*0.6]./[2.*0.6.*(2.*(1.4
+y2).*1.9-0.5.*0.5)]
E2=[0.25.*y2./(2.*(1.4+y2))].*[A2+B2.*C2]./[1-
B2.*D2]+0.5.*[C2+[D2.*(A2+B2.*C2)./(1-B2.*D2)]]./(2.*(1.4+y2))
A3=[2.*1.9.*0.6.*[40-
0.25.*0.5.*0.6.*15+0.25.*(1.3+y2).*15]+0.5.*(1+0.25.*0.6).*[35.*0.6-
0.5.*15+1.9.*0.6.*15]]./[4.*0.25.*(1.3+y2).*1.9.*0.6-
0.5.*0.5.*(1+0.25.*0.6).*(1+0.25.*0.6)]
B3=[0.6.*[(1+0.25.*0.6).*0.5.*0.5+2.*y2.*1.9.*0.6]]./[4.*0.25.*(1.3+y2).*1.9.*0.6-
0.5.*0.5.*(1+0.25.*0.6).*(1+0.25.*0.6)]
C3=[35.*0.6-0.5.*15+1.9.*0.6.*15+0.5.*(1+0.25.*0.6).*A3]./[2.*1.9.*0.6]
D3=[0.5.*(1+0.25.*0.6).*B3+0.5.*0.6]./[2.*1.9.*0.6]
```

```
E3=15.*[-0.25.*(1.3+y2).*B3+0.5.*D3+y2+0.25.*0.5.*B3-
1.9.*D3+0.5]./[2.*0.6.*[(1.4+y2)-0.25.*y2.*B3-0.5.*D3]]
F3=[35+0.25.*y2.*A3+0.5.*C3]./[2.*[1.4+y2-0.25.*y2.*B3-0.5.*D3]]
P3N=[0.6.*[35+0.25.*y2.*A1+0.5.*C1]+15.*[y2+0.5]]./[0.6.*[2.*(1.4+y2)-
0.25.*y2.*B1-0.5.*D1]]
P3R=E2+[0.6.*35+15.*(y2+0.5)]./(2.*(1.4+y2).*0.6)
P3M=[E3+F3]
plot(y2,P3N,'--k',y2,P3R,'k',y2,P3M,':k');
xlabel('\gamma_2')
ylabel('p_3');
legend('p_3^N','p_3^{RS}','p_3^{MS}')
```

S3 Fig 4. (D) Code

```
y2=0:0.1:1;
A1=[2.*1.9.*0.6.*[40-
0.25.*0.5.*0.6.*15+0.25.*(1.3+y2).*15]+0.5.*(1+0.25.*0.6).*[35.*0.6-
0.5.*15+1.9.*0.6.*15]]./[4.*0.25.*(1.3+y2).*1.9.*0.6-
0.5.*0.5.*(1+0.25.*0.6).*(1+0.25.*0.6)]
B1=[0.6.*[(1+0.25.*0.6).*0.5.*0.5+2.*y2.*1.9.*0.6]]./[4.*0.25.*(1.3+y2).*1.9.*0.6-
0.5.*0.5.*(1+0.25.*0.6).*(1+0.25.*0.6)]
C1=[35.*0.6-0.5.*15+1.9.*0.6.*15+0.5.*(1+0.25.*0.6).*A1]./[2.*1.9.*0.6]
D1=[0.5.*(1+0.25.*0.6).*B1+0.5.*0.6]./[2.*1.9.*0.6]
A2=[0.6.*[2.*(1.4+y2).*40+15.*0.25.*[2.*(1.4+y2).*(1.3+y2)-y2.*y2-
0.6.*[2.*(1.4+y2).*0.5+y2.*0.5]]]+y2.*[0.6.*35+15.*(y2+0.5)]]./[2.*0.25.*0.6.*[2.*(
1.4+y2).*(1.3+y2)-y2.*y2]]
B2=[[1+0.25.*0.6].*[2.*(1.4+y2).*0.5+y2.*0.5]]./[2.*0.25.*(2.*(1.3+y2).*(1.4+y2)-
y2.*y2)]
C2=[0.6.*[2.*(1.4+y2).*35+15.*[2.*(1.4+y2).*1.9-0.5.*0.5]+0.5.*35]+15.*[0.5.*0.5-
2.*(1.4+y2).*0.5]]./[2.*0.6.*[2.*(1.4+y2).*1.9-0.5.*0.5]]
D2=[2.*(1.4+y2).*0.5+y2.*0.5+0.25.*[2.*(1.4+y2).*0.5+y2].*0.6]./[2.*0.6.*(2.*(1.4
+y2).*1.9-0.5.*0.5)]
E2=[0.25.*y2./(2.*(1.4+y2))].*[A2+B2.*C2]./[1-
B2.*D2]+0.5.*[C2+[D2.*(A2+B2.*C2)./(1-B2.*D2)]]./(2.*(1.4+y2))
A3=[2.*1.9.*0.6.*[40-
0.25.*0.5.*0.6.*15+0.25.*(1.3+y2).*15]+0.5.*(1+0.25.*0.6).*[35.*0.6-
0.5.*15+1.9.*0.6.*15]]./[4.*0.25.*(1.3+y2).*1.9.*0.6-
0.5.*0.5.*(1+0.25.*0.6).*(1+0.25.*0.6)]
B3=[0.6.*[(1+0.25.*0.6).*0.5.*0.5+2.*y2.*1.9.*0.6]]./[4.*0.25.*(1.3+y2).*1.9.*0.6-
0.5.*0.5.*(1+0.25.*0.6).*(1+0.25.*0.6)]
C3=[35.*0.6-0.5.*15+1.9.*0.6.*15+0.5.*(1+0.25.*0.6).*A3]./[2.*1.9.*0.6]
D3=[0.5.*(1+0.25.*0.6).*B3+0.5.*0.6]./[2.*1.9.*0.6]
E3=15.*[-0.25.*(1.3+y2).*B3+0.5.*D3+y2+0.25.*0.5.*B3-
1.9.*D3+0.5]./[2.*0.6.*[(1.4+y2)-0.25.*y2.*B3-0.5.*D3]]
F3=[35+0.25.*y2.*A3+0.5.*C3]./[2.*[1.4+y2-0.25.*y2.*B3-0.5.*D3]]
P1N=A1+B1.*[0.6.*[35+0.25.*y2.*A1+0.5.*C1]+15.*[y2+0.5]]./[0.6.*[2.*(1.4+y2)-
0.25.*y2.*B1-0.5.*D1]]
P1R=(A2+B2.*C2)./(1-B2.*D2)
P1M=A3+B3.*[E3+F3]
```

```
P2M=C3+D3.*[E3+F3]
P2R=C2+[D2.*(A2+B2.*C2)]./(1-B2.*D2)
P2N=C1+D1.*[0.6.*[35+0.25.*y2.*A1+0.5.*C1]+15.*[y2+0.5]]./[0.6.*[2.*(1.4+y2)-
0.25.*y2.*B1-0.5.*D1]]
P3N=[0.6.*[35+0.25.*y2.*A1+0.5.*C1]+15.*[y2+0.5]]./[0.6.*[2.*(1.4+y2)-
0.25.*y2.*B1-0.5.*D1]]
P3R=E2+[0.6.*35+15.*(y2+0.5)]./(2.*(1.4+y2).*0.6)
P3M=[E3+F3]
Q1N=40-0.25.*(1.3+y2).*P1N+0.5.*P2N+y2.*P3N
Q2N=35-1.9.*P2N+0.25.*0.5.*P1N+0.5.*P3N
Q3N=35-(1.4+y2).*P3N+0.25.*y2.*P1N+0.5.*P2N
Q1R=40-0.25.*(1.3+y2).*P1R+0.5.*P2R+y2.*P3R
Q2R=35-1.9.*P2R+0.25.*0.5.*P1R+0.5.*P3R
Q3R=35-(1.4+y2).*P3R+0.25.*y2.*P1R+0.5.*P2R
Q1M=40-0.25.*(1.3+y2).*P1M+0.5.*P2M+y2.*P3M
Q2M=35-1.9.*P2M+0.25.*0.5.*P1M+0.5.*P3M
Q3M=35-(1.4+y2).*P3M+0.25.*y2.*P1M+0.5.*P2M
V1R=(P1N-15).*Q1N+0.6.*(P2N-15).*Q2N
V2R=(P1R-15).*Q1R+0.6.*(P2R-15).*Q2R
V3R=(P1M-15).*Q1M+0.6.*(P2M-15).*Q2M
plot(y2,V1R,'--k',y2,V2R,'k',y2,V3R,':k');
xlabel('\gamma_2')
ylabel(' V_R');
legend('V_R^N', 'V_R^{RS}', 'V_R^{MS}')
```

S3 Fig 4. (E) Code

```
y2=0:0.1:1;
A1=[2.*1.9.*0.6.*[40-
0.25.*0.5.*0.6.*15+0.25.*(1.3+y2).*15]+0.5.*(1+0.25.*0.6).*[35.*0.6-
0.5.*15+1.9.*0.6.*15]]./[4.*0.25.*(1.3+y2).*1.9.*0.6-
0.5.*0.5.*(1+0.25.*0.6).*(1+0.25.*0.6)]
B1=[0.6.*[(1+0.25.*0.6).*0.5.*0.5+2.*y2.*1.9.*0.6]]./[4.*0.25.*(1.3+y2).*1.9.*0.6-
0.5.*0.5.*(1+0.25.*0.6).*(1+0.25.*0.6)]
C1=[35.*0.6-0.5.*15+1.9.*0.6.*15+0.5.*(1+0.25.*0.6).*A1]./[2.*1.9.*0.6]
D1=[0.5.*(1+0.25.*0.6).*B1+0.5.*0.6]./[2.*1.9.*0.6]
A2=[0.6.*[2.*(1.4+y2).*40+15.*0.25.*[2.*(1.4+y2).*(1.3+y2)-y2.*y2-
0.6.*[2.*(1.4+y2).*0.5+y2.*0.5]]]+y2.*[0.6.*35+15.*(y2+0.5)]]./[2.*0.25.*0.6.*[2.*(
1.4+y2).*(1.3+y2)-y2.*y2]]
B2=[[1+0.25.*0.6].*[2.*(1.4+y2).*0.5+y2.*0.5]]./[2.*0.25.*(2.*(1.3+y2).*(1.4+y2)-
y2.*y2)]
C2=[0.6.*[2.*(1.4+y2).*35+15.*[2.*(1.4+y2).*1.9-0.5.*0.5]+0.5.*35]+15.*[0.5.*0.5-
2.*(1.4+y2).*0.5]]./[2.*0.6.*[2.*(1.4+y2).*1.9-0.5.*0.5]]
D2=[2.*(1.4+y2).*0.5+y2.*0.5+0.25.*[2.*(1.4+y2).*0.5+y2].*0.6]./[2.*0.6.*(2.*(1.4
+y2).*1.9-0.5.*0.5)]
E2=[0.25.*y2./(2.*(1.4+y2))].*[A2+B2.*C2]./[1-
B2.*D2]+0.5.*[C2+[D2.*(A2+B2.*C2)./(1-B2.*D2)]]./(2.*(1.4+y2))
A3=[2.*1.9.*0.6.*[40-
0.25.*0.5.*0.6.*15+0.25.*(1.3+y2).*15]+0.5.*(1+0.25.*0.6).*[35.*0.6-
```

```
0.5.*15+1.9.*0.6.*15]]./[4.*0.25.*(1.3+y2).*1.9.*0.6-
0.5.*0.5.*(1+0.25.*0.6).*(1+0.25.*0.6)]
B3=[0.6.*[(1+0.25.*0.6).*0.5.*0.5+2.*y2.*1.9.*0.6]]./[4.*0.25.*(1.3+y2).*1.9.*0.6-
0.5.*0.5.*(1+0.25.*0.6).*(1+0.25.*0.6)]
C3=[35.*0.6-0.5.*15+1.9.*0.6.*15+0.5.*(1+0.25.*0.6).*A3]./[2.*1.9.*0.6]
D3=[0.5.*(1+0.25.*0.6).*B3+0.5.*0.6]./[2.*1.9.*0.6]
E3=15.*[-0.25.*(1.3+y2).*B3+0.5.*D3+y2+0.25.*0.5.*B3-
1.9.*D3+0.5]./[2.*0.6.*[(1.4+y2)-0.25.*y2.*B3-0.5.*D3]]
F3=[35+0.25.*y2.*A3+0.5.*C3]./[2.*[1.4+y2-0.25.*y2.*B3-0.5.*D3]]
P1N=A1+B1.*[0.6.*[35+0.25.*y2.*A1+0.5.*C1]+15.*[y2+0.5]]./[0.6.*[2.*(1.4+y2)-
0.25.*y2.*B1-0.5.*D1]]
P1R=(A2+B2.*C2)./(1-B2.*D2)
P1M=A3+B3.*[E3+F3]
P2M=C3+D3.*[E3+F3]
P2R=C2+[D2.*(A2+B2.*C2)]./(1-B2.*D2)
P2N=C1+D1.*[0.6.*[35+0.25.*y2.*A1+0.5.*C1]+15.*[y2+0.5]]./[0.6.*[2.*(1.4+y2)-
0.25.*y2.*B1-0.5.*D1]]
P3N=[0.6.*[35+0.25.*y2.*A1+0.5.*C1]+15.*[y2+0.5]]./[0.6.*[2.*(1.4+y2)-
0.25.*y2.*B1-0.5.*D1]]
P3R=E2+[0.6.*35+15.*(y2+0.5)]./(2.*(1.4+y2).*0.6)
P3M=[E3+F3]
Q1N=40-0.25.*(1.3+y2).*P1N+0.5.*P2N+y2.*P3N
Q2N=35-1.9.*P2N+0.25.*0.5.*P1N+0.5.*P3N
Q3N=35-(1.4+y2).*P3N+0.25.*y2.*P1N+0.5.*P2N
Q1R=40-0.25.*(1.3+y2).*P1R+0.5.*P2R+y2.*P3R
Q2R=35-1.9.*P2R+0.25.*0.5.*P1R+0.5.*P3R
Q3R=35-(1.4+y2).*P3R+0.25.*y2.*P1R+0.5.*P2R
Q1M=40-0.25.*(1.3+y2).*P1M+0.5.*P2M+y2.*P3M
Q2M=35-1.9.*P2M+0.25.*0.5.*P1M+0.5.*P3M
Q3M=35-(1.4+y2).*P3M+0.25.*y2.*P1M+0.5.*P2M
V1M=0.6.*P3N.*Q3N+15.*(Q1N+Q2N)
V2M=0.6.*P3R.*Q3R+15.*(Q1R+Q2R)
V3M=0.6.*P3M.*Q3M+15.*(Q1M+Q2M)
plot(y2,V1M,'k',y2,V2M,'k',y2,V3M,':k');
xlabel('\gamma_2')
ylabel(' V_M ');
legend('V_M^N', 'V_M^{RS}', 'V_M^{MS}')
```

S3 Fig 4. (F) Code

```
y2=0:0.1:1;
A1=[2.*1.9.*0.6.*[40-
0.25.*0.5.*0.6.*15+0.25.*(1.3+y2).*15]+0.5.*(1+0.25.*0.6).*[35.*0.6-
0.5.*15+1.9.*0.6.*15]]./[4.*0.25.*(1.3+y2).*1.9.*0.6-
0.5.*0.5.*(1+0.25.*0.6).*(1+0.25.*0.6)]
B1=[0.6.*[(1+0.25.*0.6).*0.5.*0.5+2.*y2.*1.9.*0.6]]./[4.*0.25.*(1.3+y2).*1.9.*0.6-
0.5.*0.5.*(1+0.25.*0.6).*(1+0.25.*0.6)]
C1=[35.*0.6-0.5.*15+1.9.*0.6.*15+0.5.*(1+0.25.*0.6).*A1]./[2.*1.9.*0.6]
D1=[0.5.*(1+0.25.*0.6).*B1+0.5.*0.6]./[2.*1.9.*0.6]
```

```
A2=[0.6.*[2.*(1.4+y2).*40+15.*0.25.*[2.*(1.4+y2).*(1.3+y2)-y2.*y2-
0.6.*[2.*(1.4+y2).*0.5+y2.*0.5]]]+y2.*[0.6.*35+15.*(y2+0.5)]]./[2.*0.25.*0.6.*[2.*(
1.4+y2).*(1.3+y2)-y2.*y2]]
B2=[[1+0.25.*0.6].*[2.*(1.4+y2).*0.5+y2.*0.5]]./[2.*0.25.*(2.*(1.3+y2).*(1.4+y2)-
y2.*y2)]
C2=[0.6.*[2.*(1.4+y2).*35+15.*[2.*(1.4+y2).*1.9-0.5.*0.5]+0.5.*35]+15.*[0.5.*0.5-
2.*(1.4+y2).*0.5]]./[2.*0.6.*[2.*(1.4+y2).*1.9-0.5.*0.5]]
D2=[2.*(1.4+y2).*0.5+y2.*0.5+0.25.*[2.*(1.4+y2).*0.5+y2].*0.6]./[2.*0.6.*(2.*(1.4
+y2).*1.9-0.5.*0.5)]
E2=[0.25.*y2./(2.*(1.4+y2))].*[A2+B2.*C2]./[1-
B2.*D2]+0.5.*[C2+[D2.*(A2+B2.*C2)./(1-B2.*D2)]]./(2.*(1.4+y2))
A3=[2.*1.9.*0.6.*[40-
0.25.*0.5.*0.6.*15+0.25.*(1.3+y2).*15]+0.5.*(1+0.25.*0.6).*[35.*0.6-
0.5.*15+1.9.*0.6.*15]]./[4.*0.25.*(1.3+y2).*1.9.*0.6-
0.5.*0.5.*(1+0.25.*0.6).*(1+0.25.*0.6)]
B3=[0.6.*[(1+0.25.*0.6).*0.5.*0.5+2.*y2.*1.9.*0.6]]./[4.*0.25.*(1.3+y2).*1.9.*0.6-
0.5.*0.5.*(1+0.25.*0.6).*(1+0.25.*0.6)]
C3=[35.*0.6-0.5.*15+1.9.*0.6.*15+0.5.*(1+0.25.*0.6).*A3]./[2.*1.9.*0.6]
D3=[0.5.*(1+0.25.*0.6).*B3+0.5.*0.6]./[2.*1.9.*0.6]
E3=15.*[-0.25.*(1.3+y2).*B3+0.5.*D3+y2+0.25.*0.5.*B3-
1.9.*D3+0.5]./[2.*0.6.*[(1.4+y2)-0.25.*y2.*B3-0.5.*D3]]
F3=[35+0.25.*y2.*A3+0.5.*C3]./[2.*[1.4+y2-0.25.*y2.*B3-0.5.*D3]]
P1N=A1+B1.*[0.6.*[35+0.25.*y2.*A1+0.5.*C1]+15.*[y2+0.5]]./[0.6.*[2.*(1.4+y2)-
0.25.*y2.*B1-0.5.*D1]]
P1R=(A2+B2.*C2)./(1-B2.*D2)
P1M=A3+B3.*[E3+F3]
P2M=C3+D3.*[E3+F3]
P2R=C2+[D2.*(A2+B2.*C2)]./(1-B2.*D2)
P2N=C1+D1.*[0.6.*[35+0.25.*y2.*A1+0.5.*C1]+15.*[y2+0.5]]./[0.6.*[2.*(1.4+y2)-
0.25.*y2.*B1-0.5.*D1]]
P3N=[0.6.*[35+0.25.*y2.*A1+0.5.*C1]+15.*[y2+0.5]]./[0.6.*[2.*(1.4+y2)-
0.25.*y2.*B1-0.5.*D1]]
P3R=E2+[0.6.*35+15.*(y2+0.5)]./(2.*(1.4+y2).*0.6)
P3M=[E3+F3]
Q1N=40-0.25.*(1.3+y2).*P1N+0.5.*P2N+y2.*P3N
Q2N=35-1.9.*P2N+0.25.*0.5.*P1N+0.5.*P3N
Q3N=35-(1.4+y2).*P3N+0.25.*y2.*P1N+0.5.*P2N
Q1R=40-0.25.*(1.3+y2).*P1R+0.5.*P2R+y2.*P3R
Q2R=35-1.9.*P2R+0.25.*0.5.*P1R+0.5.*P3R
Q3R=35-(1.4+y2).*P3R+0.25.*y2.*P1R+0.5.*P2R
Q1M=40-0.25.*(1.3+y2).*P1M+0.5.*P2M+y2.*P3M
Q2M=35-1.9.*P2M+0.25.*0.5.*P1M+0.5.*P3M
Q3M=35-(1.4+y2).*P3M+0.25.*y2.*P1M+0.5.*P2M
V1R=(P1N-15).*Q1N+0.6.*(P2N-15).*Q2N
V2R=(P1R-15).*Q1R+0.6.*(P2R-15).*Q2R
V3R=(P1M-15).*Q1M+0.6.*(P2M-15).*Q2M
V1M=0.6.*P3N.*Q3N+15.*(Q1N+Q2N)
V2M=0.6.*P3R.*Q3R+15.*(Q1R+Q2R)
V3M=0.6.*P3M.*Q3M+15.*(Q1M+Q2M)
V1=V1R+V1M
```

```
V2=V2R+V2M
V3=V3R+V3M
plot(y2,V1,'--k',y2,V2,'k',y2,V3,':k');
xlabel('\gamma_2')
ylabel('V');
legend('V^N', 'V^{RS}', 'V^{MS}')
```

S4 Fig 5. (A) Code

```
y3=0:0.1:1;
A1=[2.*(1.4+y3).*0.6.*[40-
0.25.*0.5.*0.6.*15+0.25.*1.8.*15]+0.5.*(1+0.25.*0.6).*[35.*0.6-
0.5.*15+(1.4+y3).*0.6.*15]]./[4.*0.25.*1.8.*(1.4+y3).*0.6-
0.5.*0.5.*(1+0.25.*0.6).*(1+0.25.*0.6)]
B1=[0.6.*[(1+0.25.*0.6).*0.5.*y3+2.*0.5.*(1.4+y3).*0.6]]./[4.*0.25.*1.8.*(1.4+y3).*
0.6-0.5.*0.5.*(1+0.25.*0.6).*(1+0.25.*0.6)]
C1=[35.*0.6-0.5.*15+(1.4+y3).*0.6.*15+0.5.*(1+0.25.*0.6).*A1]./[2.*(1.4+y3).*0.6]
D1=[0.5.*(1+0.25.*0.6).*B1+y3.*0.6]./[2.*(1.4+y3).*0.6]
A2=[0.6.*[2.*(1.4+y3).*40+15.*0.25.*[2.*(1.4+y3).*1.8-0.5.*0.5-
0.6.*[2.*(1.4+y3).*0.5+0.5.*y3]]]+0.5.*[0.6.*35+15.*(0.5+y3)]]./[2.*0.25.*0.6.*[2.*(
1.4+y3).*1.8-0.5.*0.5]]
B2=[[1+0.25.*0.6].*[2.*(1.4+y3).*0.5+y3.*0.5]]./[2.*0.25.*(2.*1.8.*(1.4+y3)-
0.5.*0.5)]
C2=[0.6.*[2.*(1.4+y3).*35+15.*[2.*(1.4+y3).*(1.4+y3)-
y3.*y3]+( y3).*35]+15.*[y3.*y3-2.*(1.4+y3).*0.5]]./[2.*0.6.*[2.*(1.4+y3).*(1.4+y3)-
y3.*y3]]
D2=[2.*(1.4+y3).*0.5+0.5.*y3+0.25.*[2.*(1.4+y3).*0.5+0.5].*0.6]./[2.*0.6.*(2.*(1.4
+y3).*(1.4+y3)-y3.*y3)]
E2=[0.25.*0.5./(2.*(1.4+y3))].*[A2+B2.*C2]./[1-
B2.*D2]+y3.*[C2+[D2.*(A2+B2.*C2)./(1-B2.*D2)]]./(2.*(1.4+y3))
A3=[2.*(1.4+y3).*0.6.*[40-
0.25.*0.5.*0.6.*15+0.25.*1.8.*15]+0.5.*(1+0.25.*0.6).*[35.*0.6-
0.5.*15+(1.4+y3).*0.6.*15]]./[4.*0.25.*1.8.*(1.4+y3).*0.6-
0.5.*0.5.*(1+0.25.*0.6).*(1+0.25.*0.6)]
B3=[0.6.*[(1+0.25.*0.6).*0.5.*y3+2.*0.5.*(1.4+y3).*0.6]]./[4.*0.25.*1.8.*(1.4+y3).*
0.6-0.5.*0.5.*(1+0.25.*0.6).*(1+0.25.*0.6)]
C3=[35.*0.6-0.5.*15+(1.4+y3).*0.6.*15+0.5.*(1+0.25.*0.6).*A3]./[2.*(1.4+y3).*0.6]
D3=[0.5.*(1+0.25.*0.6).*B3+y3.*0.6]./[2.*(1.4+y3).*0.6]
E3=15.*[(-0.25.*1.8.*B3+0.5.*D3+0.5)+0.25.*0.5.*B3-
(1.4+y3).*D3+y3]./[2.*0.6.*[(1.4+y3)-0.25.*0.5.*B3-y3.*D3]]
F3=[35+0.25.*0.5.*A3+y3.*C3]./[2.*[(1.4+y3)-0.25.*0.5.*B3-y3.*D3]]
P1N=A1+B1.*[0.6.*[35+0.25.*0.5.*A1+y3.*C1]+15.*[0.5+y3]]./[0.6.*[2.*(1.4+y3)-
0.25.*0.5.*B1-y3.*D1]]
P1R=(A2+B2.*C2)./(1-B2.*D2)
P1M=A3+B3.*[E3+F3]
plot(y3,P1N,'--k',y3,P1R,'k',y3,P1M,':k');
xlabel('\gamma_3')
ylabel('p_1');
legend('p_1^N', 'p_1^{RS}', 'p_1^{MS}')
```

S4 Fig 5. (B) Code

```
y3=0:0.1:1;
A1=[2.*(1.4+y3).*0.6.*[40-
0.25.*0.5.*0.6.*15+0.25.*1.8.*15]+0.5.*(1+0.25.*0.6).*[35.*0.6-
0.5.*15+(1.4+y3).*0.6.*15]]./[4.*0.25.*1.8.*(1.4+y3).*0.6-
0.5.*0.5.*(1+0.25.*0.6).*(1+0.25.*0.6)]
B1=[0.6.*[(1+0.25.*0.6).*0.5.*y3+2.*0.5.*(1.4+y3).*0.6]]./[4.*0.25.*1.8.*(1.4+y3).*
0.6-0.5.*0.5.*(1+0.25.*0.6).*(1+0.25.*0.6)]
C1=[35.*0.6-0.5.*15+(1.4+y3).*0.6.*15+0.5.*(1+0.25.*0.6).*A1]./[2.*(1.4+y3).*0.6]
D1=[0.5.*(1+0.25.*0.6).*B1+y3.*0.6]./[2.*(1.4+y3).*0.6]
A2=[0.6.*[2.*(1.4+y3).*40+15.*0.25.*[2.*(1.4+y3).*1.8-0.5.*0.5-
0.6.*[2.*(1.4+y3).*0.5+0.5.*y3]]]+0.5.*[0.6.*35+15.*(0.5+y3)]]./[2.*0.25.*0.6.*[2.*(
1.4+y3).*1.8-0.5.*0.5]]
B2=[[1+0.25.*0.6].*[2.*(1.4+y3).*0.5+y3.*0.5]]./[2.*0.25.*(2.*1.8.*(1.4+y3)-
0.5.*0.5)]
C2=[0.6.*[2.*(1.4+y3).*35+15.*[2.*(1.4+y3).*(1.4+y3)-
y3.*y3]+( y3).*35]+15.*[y3.*y3-2.*(1.4+y3).*0.5]]./[2.*0.6.*[2.*(1.4+y3).*(1.4+y3)-
y3.*y3]]
D2=[2.*(1.4+y3).*0.5+0.5.*y3+0.25.*[2.*(1.4+y3).*0.5+0.5].*0.6]./[2.*0.6.*(2.*(1.4
+y3).*(1.4+y3)-y3.*y3)]
E2=[0.25.*0.5./(2.*(1.4+y3))].*[A2+B2.*C2]./[1-
B2.*D2]+y3.*[C2+[D2.*(A2+B2.*C2)./(1-B2.*D2)]]./(2.*(1.4+y3))
A3=[2.*(1.4+y3).*0.6.*[40-
0.25.*0.5.*0.6.*15+0.25.*1.8.*15]+0.5.*(1+0.25.*0.6).*[35.*0.6-
0.5.*15+(1.4+y3).*0.6.*15]]./[4.*0.25.*1.8.*(1.4+y3).*0.6-
0.5.*0.5.*(1+0.25.*0.6).*(1+0.25.*0.6)]
B3=[0.6.*[(1+0.25.*0.6).*0.5.*y3+2.*0.5.*(1.4+y3).*0.6]]./[4.*0.25.*1.8.*(1.4+y3).*
0.6-0.5.*0.5.*(1+0.25.*0.6).*(1+0.25.*0.6)]
C3=[35.*0.6-0.5.*15+(1.4+y3).*0.6.*15+0.5.*(1+0.25.*0.6).*A3]./[2.*(1.4+y3).*0.6]
D3=[0.5.*(1+0.25.*0.6).*B3+y3.*0.6]./[2.*(1.4+y3).*0.6]
E3=15.*[(-0.25.*1.8.*B3+0.5.*D3+0.5)+0.25.*0.5.*B3-
(1.4+y3).*D3+y3]./[2.*0.6.*[(1.4+y3)-0.25.*0.5.*B3-y3.*D3]]
F3=[35+0.25.*0.5.*A3+y3.*C3]./[2.*[(1.4+y3)-0.25.*0.5.*B3-y3.*D3]]
P2M=C3+D3.*[E3+F3]
P2R=C2+[D2.*(A2+B2.*C2)]./(1-B2.*D2)
P2N=C1+D1.*[0.6.*[35+0.25.*0.5.*A1+y3.*C1]+15.*[0.5+y3]]./[0.6.*[2.*(1.4+y3)-
0.25.*0.5.*B1-y3.*D1]]
plot(y3,P2N,'--k',y3,P2R,'k',y3,P2M,':k');
xlabel('\gamma_3')
ylabel('p_2');
legend('p_2^N', 'p_2^{RS}', 'p_2^{MS}')
```

S4 Fig 5. (C) Code

```
y3=0:0.1:1;
A1=[2.*(1.4+y3).*0.6.*[40-
0.25.*0.5.*0.6.*15+0.25.*1.8.*15]+0.5.*(1+0.25.*0.6).*[35.*0.6-
```

```
0.5.*15+(1.4+y3).*0.6.*15]]./[4.*0.25.*1.8.*(1.4+y3).*0.6-
0.5.*0.5.*(1+0.25.*0.6).*(1+0.25.*0.6)]
B1=[0.6.*[(1+0.25.*0.6).*0.5.*y3+2.*0.5.*(1.4+y3).*0.6]]./[4.*0.25.*1.8.*(1.4+y3).*
0.6-0.5.*0.5.*(1+0.25.*0.6).*(1+0.25.*0.6)]
C1=[35.*0.6-0.5.*15+(1.4+y3).*0.6.*15+0.5.*(1+0.25.*0.6).*A1]./[2.*(1.4+y3).*0.6]
D1=[0.5.*(1+0.25.*0.6).*B1+y3.*0.6]./[2.*(1.4+y3).*0.6]
A2=[0.6.*[2.*(1.4+y3).*40+15.*0.25.*[2.*(1.4+y3).*1.8-0.5.*0.5-
0.6.*[2.*(1.4+y3).*0.5+0.5.*y3]]]+0.5.*[0.6.*35+15.*(0.5+y3)]]./[2.*0.25.*0.6.*[2.*(
1.4+y3).*1.8-0.5.*0.5]]
B2=[[1+0.25.*0.6].*[2.*(1.4+y3).*0.5+y3.*0.5]]./[2.*0.25.*(2.*1.8.*(1.4+y3)-
0.5.*0.5)]
C2=[0.6.*[2.*(1.4+y3).*35+15.*[2.*(1.4+y3).*(1.4+y3)-
y3.*y3]+( y3).*35]+15.*[y3.*y3-2.*(1.4+y3).*0.5]]./[2.*0.6.*[2.*(1.4+y3).*(1.4+y3)-
y3.*y3]]
D2=[2.*(1.4+y3).*0.5+0.5.*y3+0.25.*[2.*(1.4+y3).*0.5+0.5].*0.6]./[2.*0.6.*(2.*(1.4
+y3).*(1.4+y3)-y3.*y3)]
E2=[0.25.*0.5./(2.*(1.4+y3))].*[A2+B2.*C2]./[1-
B2.*D2]+y3.*[C2+[D2.*(A2+B2.*C2)./(1-B2.*D2)]]./(2.*(1.4+y3))
A3=[2.*(1.4+y3).*0.6.*[40-
0.25.*0.5.*0.6.*15+0.25.*1.8.*15]+0.5.*(1+0.25.*0.6).*[35.*0.6-
0.5.*15+(1.4+y3).*0.6.*15]]./[4.*0.25.*1.8.*(1.4+y3).*0.6-
0.5.*0.5.*(1+0.25.*0.6).*(1+0.25.*0.6)]
B3=[0.6.*[(1+0.25.*0.6).*0.5.*y3+2.*0.5.*(1.4+y3).*0.6]]./[4.*0.25.*1.8.*(1.4+y3).*
0.6-0.5.*0.5.*(1+0.25.*0.6).*(1+0.25.*0.6)]
C3=[35.*0.6-0.5.*15+(1.4+y3).*0.6.*15+0.5.*(1+0.25.*0.6).*A3]./[2.*(1.4+y3).*0.6]
D3=[0.5.*(1+0.25.*0.6).*B3+y3.*0.6]./[2.*(1.4+y3).*0.6]
E3=15.*[(-0.25.*1.8.*B3+0.5.*D3+0.5)+0.25.*0.5.*B3-
(1.4+y3).*D3+y3]./[2.*0.6.*[(1.4+y3)-0.25.*0.5.*B3-y3.*D3]]
F3=[35+0.25.*0.5.*A3+y3.*C3]./[2.*[(1.4+y3)-0.25.*0.5.*B3-y3.*D3]]
P3N=[0.6.*[35+0.25.*0.5.*A1+y3.*C1]+15.*[0.5+y3]]./[0.6.*[2.*(1.4+y3)-
0.25.*0.5.*B1-y3.*D1]]
P3R=E2+[0.6.*35+15.*(0.5+y3)]./(2.*(1.4+y3).*0.6)
P3M=E3 +F3
plot(y3,P3N,'--k',y3,P3R,'k',y3,P3M,':k');
xlabel('\gamma_3')
ylabel('p_3');
legend('p_3^N', 'p_3^{RS}', 'p_3^{MS}')
```

S4 Fig 5. (D) Code

```
y3=0:0.1:1;
A1=[2.*(1.4+y3).*0.6.*[40-
0.25.*0.5.*0.6.*15+0.25.*1.8.*15]+0.5.*(1+0.25.*0.6).*[35.*0.6-
0.5.*15+(1.4+y3).*0.6.*15]]./[4.*0.25.*1.8.*(1.4+y3).*0.6-
0.5.*0.5.*(1+0.25.*0.6).*(1+0.25.*0.6)]
B1=[0.6.*[(1+0.25.*0.6).*0.5.*y3+2.*0.5.*(1.4+y3).*0.6]]./[4.*0.25.*1.8.*(1.4+y3).*
0.6-0.5.*0.5.*(1+0.25.*0.6).*(1+0.25.*0.6)]
C1=[35.*0.6-0.5.*15+(1.4+y3).*0.6.*15+0.5.*(1+0.25.*0.6).*A1]./[2.*(1.4+y3).*0.6]
D1=[0.5.*(1+0.25.*0.6).*B1+y3.*0.6]./[2.*(1.4+y3).*0.6]
```

```
A2=[0.6.*[2.*(1.4+y3).*40+15.*0.25.*[2.*(1.4+y3).*1.8-0.5.*0.5-
0.6.*[2.*(1.4+y3).*0.5+0.5.*y3]]]+0.5.*[0.6.*35+15.*(0.5+y3)]]./[2.*0.25.*0.6.*[2.*(
1.4+y3).*1.8-0.5.*0.5]]
B2=[[1+0.25.*0.6].*[2.*(1.4+y3).*0.5+y3.*0.5]]./[2.*0.25.*(2.*1.8.*(1.4+y3)-
0.5.*0.5)]
C2=[0.6.*[2.*(1.4+y3).*35+15.*[2.*(1.4+y3).*(1.4+y3)-
y3.*y3]+( y3).*35]+15.*[y3.*y3-2.*(1.4+y3).*0.5]]./[2.*0.6.*[2.*(1.4+y3).*(1.4+y3)-
y3.*y3]]
D2=[2.*(1.4+y3).*0.5+0.5.*y3+0.25.*[2.*(1.4+y3).*0.5+0.5].*0.6]./[2.*0.6.*(2.*(1.4
+y3).*(1.4+y3)-y3.*y3)]
E2=[0.25.*0.5./(2.*(1.4+y3))].*[A2+B2.*C2]./[1-
B2.*D2]+y3.*[C2+[D2.*(A2+B2.*C2)./(1-B2.*D2)]]./(2.*(1.4+y3))
A3=[2.*(1.4+y3).*0.6.*[40-
0.25.*0.5.*0.6.*15+0.25.*1.8.*15]+0.5.*(1+0.25.*0.6).*[35.*0.6-
0.5.*15+(1.4+y3).*0.6.*15]]./[4.*0.25.*1.8.*(1.4+y3).*0.6-
0.5.*0.5.*(1+0.25.*0.6).*(1+0.25.*0.6)]
B3=[0.6.*[(1+0.25.*0.6).*0.5.*y3+2.*0.5.*(1.4+y3).*0.6]]./[4.*0.25.*1.8.*(1.4+y3).*
0.6-0.5.*0.5.*(1+0.25.*0.6).*(1+0.25.*0.6)]
C3=[35.*0.6-0.5.*15+(1.4+y3).*0.6.*15+0.5.*(1+0.25.*0.6).*A3]./[2.*(1.4+y3).*0.6]
D3=[0.5.*(1+0.25.*0.6).*B3+y3.*0.6]./[2.*(1.4+y3).*0.6]
E3=15.*[(-0.25.*1.8.*B3+0.5.*D3+0.5)+0.25.*0.5.*B3-
(1.4+y3).*D3+y3]./[2.*0.6.*[(1.4+y3)-0.25.*0.5.*B3-y3.*D3]]
F3=[35+0.25.*0.5.*A3+y3.*C3]./[2.*[(1.4+y3)-0.25.*0.5.*B3-y3.*D3]]
P1N=A1+B1.*[0.6.*[35+0.25.*0.5.*A1+y3.*C1]+15.*[0.5+y3]]./[0.6.*[2.*(1.4+y3)-
0.25.*0.5.*B1-y3.*D1]]
P1R=(A2+B2.*C2)./(1-B2.*D2)
P1M=A3+B3.*[E3+F3]
P2M=C3+D3.*[E3+F3]
P2R=C2+[D2.*(A2+B2.*C2)]./(1-B2.*D2)
P2N=C1+D1.*[0.6.*[35+0.25.*0.5.*A1+y3.*C1]+15.*[0.5+y3]]./[0.6.*[2.*(1.4+y3)-
0.25.*0.5.*B1-y3.*D1]]
P3N=[0.6.*[35+0.25.*0.5.*A1+y3.*C1]+15.*[0.5+y3]]./[0.6.*[2.*(1.4+y3)-
0.25.*0.5.*B1-y3.*D1]]
P3R=E2+[0.6.*35+15.*(0.5+y3)]./(2.*(1.4+y3).*0.6)
P3M=E3 +F3
Q1N=40-0.25.*1.8.*P1N+0.5.*P2N+0.5.*P3N
Q2N=35-(1.4+y3).*P2N+0.25.*0.5.*P1N+y3.*P3N
Q3N=35-(1.4+y3).*P3N+0.25.*0.5.*P1N+y3.*P2N
Q1R=40-0.25.*1.8.*P1R+0.5.*P2R+0.5.*P3R
Q2R=35-(1.4+y3).*P2R+0.25.*0.5.*P1R+y3.*P3R
Q3R=35-(1.4+y3).*P3R+0.25.*0.5.*P1R+y3.*P2R
Q1M=40-0.25.*1.8.*P1M+0.5.*P2M+0.5.*P3M
Q2M=35-(1.4+y3).*P2M+0.25.*0.5.*P1M+y3.*P3M
Q3M=35-(1.4+y3).*P3M+0.25.*0.5.*P1M+y3.*P2M
V1R=(P1N-15).*Q1N+0.6.*(P2N-15).*Q2N
V2R=(P1R-15).*Q1R+0.6.*(P2R-15).*Q2R
V3R=(P1M-15).*Q1M+0.6.*(P2M-15).*Q2M
plot(y3,V1R,'--k',y3,V2R,'k',y3,V3R,':k');
xlabel('\gamma_3')
ylabel(' V_R');
```

```
legend('V_R^N', 'V_R^{RS}', 'V_R^{MS}')
```

S4 Fig 5. (E) Code

```
y3=0:0.1:1;
A1=[2.*(1.4+y3).*0.6.*[40-
0.25.*0.5.*0.6.*15+0.25.*1.8.*15]+0.5.*(1+0.25.*0.6).*[35.*0.6-
0.5.*15+(1.4+y3).*0.6.*15]]./[4.*0.25.*1.8.*(1.4+y3).*0.6-
0.5.*0.5.*(1+0.25.*0.6).*(1+0.25.*0.6)]
B1=[0.6.*[(1+0.25.*0.6).*0.5.*y3+2.*0.5.*(1.4+y3).*0.6]]./[4.*0.25.*1.8.*(1.4+y3).*
0.6-0.5.*0.5.*(1+0.25.*0.6).*(1+0.25.*0.6)]
C1=[35.*0.6-0.5.*15+(1.4+y3).*0.6.*15+0.5.*(1+0.25.*0.6).*A1]./[2.*(1.4+y3).*0.6]
D1=[0.5.*(1+0.25.*0.6).*B1+y3.*0.6]./[2.*(1.4+y3).*0.6]
A2=[0.6.*[2.*(1.4+y3).*40+15.*0.25.*[2.*(1.4+y3).*1.8-0.5.*0.5-
0.6.*[2.*(1.4+y3).*0.5+0.5.*y3]]]+0.5.*[0.6.*35+15.*(0.5+y3)]]./[2.*0.25.*0.6.*[2.*(
1.4+y3).*1.8-0.5.*0.5]]
B2=[[1+0.25.*0.6].*[2.*(1.4+y3).*0.5+y3.*0.5]]./[2.*0.25.*(2.*1.8.*(1.4+y3)-
0.5.*0.5)]
C2=[0.6.*[2.*(1.4+y3).*35+15.*[2.*(1.4+y3).*(1.4+y3)-
y3.*y3]+( y3).*35]+15.*[y3.*y3-2.*(1.4+y3).*0.5]]./[2.*0.6.*[2.*(1.4+y3).*(1.4+y3)-
y3.*y3]]
D2=[2.*(1.4+y3).*0.5+0.5.*y3+0.25.*[2.*(1.4+y3).*0.5+0.5].*0.6]./[2.*0.6.*(2.*(1.4
+y3).*(1.4+y3)-y3.*y3)]
E2=[0.25.*0.5./(2.*(1.4+y3))].*[A2+B2.*C2]./[1-
B2.*D2]+y3.*[C2+[D2.*(A2+B2.*C2)./(1-B2.*D2)]]./(2.*(1.4+y3))
A3=[2.*(1.4+y3).*0.6.*[40-
0.25.*0.5.*0.6.*15+0.25.*1.8.*15]+0.5.*(1+0.25.*0.6).*[35.*0.6-
0.5.*15+(1.4+y3).*0.6.*15]]./[4.*0.25.*1.8.*(1.4+y3).*0.6-
0.5.*0.5.*(1+0.25.*0.6).*(1+0.25.*0.6)]
B3=[0.6.*[(1+0.25.*0.6).*0.5.*y3+2.*0.5.*(1.4+y3).*0.6]]./[4.*0.25.*1.8.*(1.4+y3).*
0.6-0.5.*0.5.*(1+0.25.*0.6).*(1+0.25.*0.6)]
C3=[35.*0.6-0.5.*15+(1.4+y3).*0.6.*15+0.5.*(1+0.25.*0.6).*A3]./[2.*(1.4+y3).*0.6]
D3=[0.5.*(1+0.25.*0.6).*B3+y3.*0.6]./[2.*(1.4+y3).*0.6]
E3=15.*[(-0.25.*1.8.*B3+0.5.*D3+0.5)+0.25.*0.5.*B3-
(1.4+y3).*D3+y3]./[2.*0.6.*[(1.4+y3)-0.25.*0.5.*B3-y3.*D3]]
F3=[35+0.25.*0.5.*A3+y3.*C3]./[2.*[(1.4+y3)-0.25.*0.5.*B3-y3.*D3]]
P1N=A1+B1.*[0.6.*[35+0.25.*0.5.*A1+y3.*C1]+15.*[0.5+y3]]./[0.6.*[2.*(1.4+y3)-
0.25.*0.5.*B1-y3.*D1]]
P1R=(A2+B2.*C2)./(1-B2.*D2)
P1M=A3+B3.*[E3+F3]
P2M=C3+D3.*[E3+F3]
P2R=C2+[D2.*(A2+B2.*C2)]./(1-B2.*D2)
P2N=C1+D1.*[0.6.*[35+0.25.*0.5.*A1+y3.*C1]+15.*[0.5+y3]]./[0.6.*[2.*(1.4+y3)-
0.25.*0.5.*B1-y3.*D1]]
P3N=[0.6.*[35+0.25.*0.5.*A1+y3.*C1]+15.*[0.5+y3]]./[0.6.*[2.*(1.4+y3)-
0.25.*0.5.*B1-y3.*D1]]
P3R=E2+[0.6.*35+15.*(0.5+y3)]./(2.*(1.4+y3).*0.6)
P3M=E3 +F3
Q1N=40-0.25.*1.8.*P1N+0.5.*P2N+0.5.*P3N
```

```
Q2N=35-(1.4+y3).*P2N+0.25.*0.5.*P1N+y3.*P3N
Q3N=35-(1.4+y3).*P3N+0.25.*0.5.*P1N+y3.*P2N
Q1R=40-0.25.*1.8.*P1R+0.5.*P2R+0.5.*P3R
Q2R=35-(1.4+y3).*P2R+0.25.*0.5.*P1R+y3.*P3R
Q3R=35-(1.4+y3).*P3R+0.25.*0.5.*P1R+y3.*P2R
Q1M=40-0.25.*1.8.*P1M+0.5.*P2M+0.5.*P3M
Q2M=35-(1.4+y3).*P2M+0.25.*0.5.*P1M+y3.*P3M
Q3M=35-(1.4+y3).*P3M+0.25.*0.5.*P1M+y3.*P2M
V1M=0.6.*P3N.*Q3N+15.*(Q1N+Q2N)
V2M=0.6.*P3R.*Q3R+15.*(Q1R+Q2R)
V3M=0.6.*P3M.*Q3M+15.*(Q1M+Q2M)
plot(y3,V1M,'k',y3,V2M,'k',y3,V3M,':k');
xlabel('\gamma_3')
ylabel(' V_M');
legend('V_M^N', 'V_M^{RS}', 'V_M^{MS}')
```

S4 Fig 5. (F) Code

```
y3=0:0.1:1;
A1=[2.*(1.4+y3).*0.6.*[40-
0.25.*0.5.*0.6.*15+0.25.*1.8.*15]+0.5.*(1+0.25.*0.6).*[35.*0.6-
0.5.*15+(1.4+y3).*0.6.*15]]./[4.*0.25.*1.8.*(1.4+y3).*0.6-
0.5.*0.5.*(1+0.25.*0.6).*(1+0.25.*0.6)]
B1=[0.6.*[(1+0.25.*0.6).*0.5.*y3+2.*0.5.*(1.4+y3).*0.6]]./[4.*0.25.*1.8.*(1.4+y3).*
0.6-0.5.*0.5.*(1+0.25.*0.6).*(1+0.25.*0.6)]
C1=[35.*0.6-0.5.*15+(1.4+y3).*0.6.*15+0.5.*(1+0.25.*0.6).*A1]./[2.*(1.4+y3).*0.6]
D1=[0.5.*(1+0.25.*0.6).*B1+y3.*0.6]./[2.*(1.4+y3).*0.6]
A2=[0.6.*[2.*(1.4+y3).*40+15.*0.25.*[2.*(1.4+y3).*1.8-0.5.*0.5-
0.6.*[2.*(1.4+y3).*0.5+0.5.*y3]]]+0.5.*[0.6.*35+15.*(0.5+y3)]]./[2.*0.25.*0.6.*[2.*(
1.4+y3).*1.8-0.5.*0.5]]
B2=[[1+0.25.*0.6].*[2.*(1.4+y3).*0.5+y3.*0.5]]./[2.*0.25.*(2.*1.8.*(1.4+y3)-
0.5.*0.5)]
C2=[0.6.*[2.*(1.4+y3).*35+15.*[2.*(1.4+y3).*(1.4+y3)-
y3.*y3]+( y3).*35]+15.*[y3.*y3-2.*(1.4+y3).*0.5]]./[2.*0.6.*[2.*(1.4+y3).*(1.4+y3)-
y3.*y3]]
D2=[2.*(1.4+y3).*0.5+0.5.*y3+0.25.*[2.*(1.4+y3).*0.5+0.5].*0.6]./[2.*0.6.*(2.*(1.4
+y3).*(1.4+y3)-y3.*y3)]
E2=[0.25.*0.5./(2.*(1.4+y3))].*[A2+B2.*C2]./[1-
B2.*D2]+y3.*[C2+[D2.*(A2+B2.*C2)./(1-B2.*D2)]]./(2.*(1.4+y3))
A3=[2.*(1.4+y3).*0.6.*[40-
0.25.*0.5.*0.6.*15+0.25.*1.8.*15]+0.5.*(1+0.25.*0.6).*[35.*0.6-
0.5.*15+(1.4+y3).*0.6.*15]]./[4.*0.25.*1.8.*(1.4+y3).*0.6-
0.5.*0.5.*(1+0.25.*0.6).*(1+0.25.*0.6)]
B3=[0.6.*[(1+0.25.*0.6).*0.5.*y3+2.*0.5.*(1.4+y3).*0.6]]./[4.*0.25.*1.8.*(1.4+y3).*
0.6-0.5.*0.5.*(1+0.25.*0.6).*(1+0.25.*0.6)]
C3=[35.*0.6-0.5.*15+(1.4+y3).*0.6.*15+0.5.*(1+0.25.*0.6).*A3]./[2.*(1.4+y3).*0.6]
D3=[0.5.*(1+0.25.*0.6).*B3+y3.*0.6]./[2.*(1.4+y3).*0.6]
E3=15.*[(-0.25.*1.8.*B3+0.5.*D3+0.5)+0.25.*0.5.*B3-
(1.4+y3).*D3+y3]./[2.*0.6.*[(1.4+y3)-0.25.*0.5.*B3-y3.*D3]]
```

```
F3=[35+0.25.*0.5.*A3+y3.*C3]./[2.*[(1.4+y3)-0.25.*0.5.*B3-y3.*D3]]
P1N=A1+B1.*[0.6.*[35+0.25.*0.5.*A1+y3.*C1]+15.*[0.5+y3]]./[0.6.*[2.*(1.4+y3)-
0.25.*0.5.*B1-y3.*D1]]
P1R=(A2+B2.*C2)./(1-B2.*D2)
P1M=A3+B3.*[E3+F3]
P2M=C3+D3.*[E3+F3]
P2R=C2+[D2.*(A2+B2.*C2)]./(1-B2.*D2)
P2N=C1+D1.*[0.6.*[35+0.25.*0.5.*A1+y3.*C1]+15.*[0.5+y3]]./[0.6.*[2.*(1.4+y3)-
0.25.*0.5.*B1-y3.*D1]]
P3N=[0.6.*[35+0.25.*0.5.*A1+y3.*C1]+15.*[0.5+y3]]./[0.6.*[2.*(1.4+y3)-
0.25.*0.5.*B1-y3.*D1]]
P3R=E2+[0.6.*35+15.*(0.5+y3)]./(2.*(1.4+y3).*0.6)
P3M=E3 +F3
Q1N=40-0.25.*1.8.*P1N+0.5.*P2N+0.5.*P3N
Q2N=35-(1.4+y3).*P2N+0.25.*0.5.*P1N+y3.*P3N
Q3N=35-(1.4+y3).*P3N+0.25.*0.5.*P1N+y3.*P2N
Q1R=40-0.25.*1.8.*P1R+0.5.*P2R+0.5.*P3R
Q2R=35-(1.4+y3).*P2R+0.25.*0.5.*P1R+y3.*P3R
Q3R=35-(1.4+y3).*P3R+0.25.*0.5.*P1R+y3.*P2R
Q1M=40-0.25.*1.8.*P1M+0.5.*P2M+0.5.*P3M
Q2M=35-(1.4+y3).*P2M+0.25.*0.5.*P1M+y3.*P3M
Q3M=35-(1.4+y3).*P3M+0.25.*0.5.*P1M+y3.*P2M
V1R=(P1N-15).*Q1N+0.6.*(P2N-15).*Q2N
V2R=(P1R-15).*Q1R+0.6.*(P2R-15).*Q2R
V3R=(P1M-15).*Q1M+0.6.*(P2M-15).*Q2M
V1M=0.6.*P3N.*Q3N+15.*(Q1N+Q2N)
V2M=0.6.*P3R.*Q3R+15.*(Q1R+Q2R)
V3M=0.6.*P3M.*Q3M+15.*(Q1M+Q2M)
V1=V1R+V1M
V2=V2R+V2M
V3=V3R+V3M
plot(y3,V1,'--k',y3,V2,'k',y3,V3,':k');
xlabel('\gamma_3')
ylabel('V');
legend('V^N', 'V^{RS}', 'V^{MS}')
```

S5 Fig 6. (A) Code

```
[y1 y3]=meshgrid(0:0.1:1,0:0.1:1);
A1=[2.*(0.9+y1+y3).*0.6.*[40-
0.3.*y1.*0.6.*15+0.3.*(1.3+y1).*15]+y1.*(1+0.3.*0.6).*[35.*0.6-
y1.*15+(0.9+y1+y3).*0.6.*15]]./[4.*0.3.*(1.3+y1).*(0.9+y1+y3).*0.6-
y1.*y1.*(1+0.3.*0.6).*(1+0.3.*0.6)]
B1=[0.6.*[(1+0.3.*0.6).*y1.*y3+2.*0.5.*(0.9+y1+y3).*0.6]]./[4.*0.3.*(1.3+y1).*(0.9
+y1+y3).*0.6-y1.*y1.*(1+0.3.*0.6).*(1+0.3.*0.6)]
C1=[35.*0.6-
y1.*15+(0.9+y1+y3).*0.6.*15+y1.*(1+0.3.*0.6).*A1]./[2.*(0.9+y1+y3).*0.6]
D1=[y1.*(1+0.3.*0.6).*B1+y3.*0.6]./[2.*(0.9+y1+y3).*0.6]
A2=[0.6.*[2.*(1.4+y3).*40+15.*0.3.*[2.*(1.4+y3).*(1.3+y1)-0.5.*0.5-
```

```
0.6.*[2.*(1.4+y3).*y1+0.5.*y3]]]+0.5.*[0.6.*35+15.*(0.5+y3)]]./[2.*0.3.*0.6.*[2.*(1.4+y3).*(1.3+y1)-0.5.*0.5]]
B2=[[1+0.3.*0.6].*[2.*(1.4+y3).*y1+y3.*0.5]]./[2.*0.3.*(2.*(1.3+y1).*(1.4+y3)-0.5.*0.5)]
C2=[0.6.*[2.*(1.4+y3).*35+15.*[2.*(1.4+y3).*(0.9+y1+y3)-y3.*y3]+( y3).*35]+15.*[y3.*y3-2.*(1.4+y3).*y1]]./[2.*0.6.*[2.*(1.4+y3).*(0.9+y1+y3)-y3.*y3]]
D2=[2.*(1.4+y3).*y1+0.5.*y3+0.3.*[2.*(1.4+y3).*y1+0.5].*0.6]./[2.*0.6.*(2.*(1.4+y3).*(0.9+y1+y3)-y3.*y3)]
E2=[0.3.*0.5./(2.*(1.4+y3))].*[A2+B2.*C2]./[1-B2.*D2]+y3.*[C2+[D2.*(A2+B2.*C2)./(1-B2.*D2)]]./(2.*(1.4+y3))
A3=[2.*(0.9+y1+y3).*0.6.*[40-0.3.*y1.*0.6.*15+0.3.*(1.3+y1).*15]+y1.*(1+0.3.*0.6).*[35.*0.6-y1.*15+(0.9+y1+y3).*0.6.*15]]./[4.*0.3.*(1.3+y1).*(0.9+y1+y3).*0.6-y1.*y1.*(1+0.3.*0.6).*(1+0.3.*0.6)]
B3=[0.6.*[(1+0.3.*0.6).*y1.*y3+2.*0.5.*(0.9+y1+y3).*0.6]]./[4.*0.3.*(1.3+y1).*(0.9+y1+y3).*0.6-y1.*y1.*(1+0.3.*0.6).*(1+0.3.*0.6)]
C3=[35.*0.6-y1.*15+(0.9+y1+y3).*0.6.*15+y1.*(1+0.3.*0.6).*A3]./[2.*(0.9+y1+y3).*0.6]
D3=[y1.*(1+0.3.*0.6).*B3+y3.*0.6]./[2.*(0.9+y1+y3).*0.6]
E3=15.*[(-0.3.*(1.3+y1).*B3+y1.*D3+0.5)+0.3.*y1.*B3-(0.9+y1+y3).*D3+y3]./[2.*0.6.*[(1.4+y3)-0.3.*0.5.*B3-y3.*D3]]
F3=[35+0.3.*0.5.*A3+y3.*C3]./[2.*[(1.4+y3)-0.3.*0.5.*B3-y3.*D3]]
P1N=A1+B1.*[0.6.*[35+0.3.*0.5.*A1+y3.*C1]+15.*[0.5+y3]]./[0.6.*[2.*(1.4+y3)-0.3.*0.5.*B1-y3.*D1]]
P1R=(A2+B2.*C2)./(1-B2.*D2)
P1M=A3+B3.*[E3+F3]
surf(y1,y3,P1N);
hold on
surf(y1,y3,P1R);
surf(y1,y3,P1M);
xlabel('\gamma_1');
ylabel('\gamma_3');
zlabel('p_1')
legend('p_1^N', 'p_1^{RS}', 'p_1^{MS}')
hold off
```

S5 Fig 6. (B) Code

```
[y1 y3]=meshgrid(0:0.1:1,0:0.1:1);
A1=[2.*(0.9+y1+y3).*0.6.*[40-0.3.*y1.*0.6.*15+0.3.*(1.3+y1).*15]+y1.*(1+0.3.*0.6).*[35.*0.6-y1.*15+(0.9+y1+y3).*0.6.*15]]./[4.*0.3.*(1.3+y1).*(0.9+y1+y3).*0.6-y1.*y1.*(1+0.3.*0.6).*(1+0.3.*0.6)]
B1=[0.6.*[(1+0.3.*0.6).*y1.*y3+2.*0.5.*(0.9+y1+y3).*0.6]]./[4.*0.3.*(1.3+y1).*(0.9+y1+y3).*0.6-y1.*y1.*(1+0.3.*0.6).*(1+0.3.*0.6)]
C1=[35.*0.6-y1.*15+(0.9+y1+y3).*0.6.*15+y1.*(1+0.3.*0.6).*A1]./[2.*(0.9+y1+y3).*0.6]
```

```
D1=[y1.*(1+0.3.*0.6).*B1+y3.*0.6]./[2.*(0.9+y1+y3).*0.6]
A2=[0.6.*[2.*(1.4+y3).*40+15.*0.3.*[2.*(1.4+y3).*(1.3+y1)-0.5.*0.5-
0.6.*[2.*(1.4+y3).*y1+0.5.*y3]]]+0.5.*[0.6.*35+15.*(0.5+y3)]]./[2.*0.3.*0.6.*[2.*(1.
4+y3).*(1.3+y1)-0.5.*0.5]]
B2=[[1+0.3.*0.6].*[2.*(1.4+y3).*y1+y3.*0.5]]./[2.*0.3.*(2.*(1.3+y1).*(1.4+y3)-
0.5.*0.5)]
C2=[0.6.*[2.*(1.4+y3).*35+15.*[2.*(1.4+y3).*(0.9+y1+y3)-
y3.*y3]+( y3).*35]+15.*[y3.*y3-
2.*(1.4+y3).*y1]]./[2.*0.6.*[2.*(1.4+y3).*(0.9+y1+y3)-y3.*y3]]
D2=[2.*(1.4+y3).*y1+0.5.*y3+0.3.*[2.*(1.4+y3).*y1+0.5].*0.6]./[2.*0.6.*(2.*(1.4+y
3).*(0.9+y1+y3)-y3.*y3)]
E2=[0.3.*0.5./(2.*(1.4+y3))].*[A2+B2.*C2]./[1-
B2.*D2]+y3.*[C2+[D2.*(A2+B2.*C2)./(1-B2.*D2)]]./(2.*(1.4+y3))
A3=[2.*(0.9+y1+y3).*0.6.*[40-
0.3.*y1.*0.6.*15+0.3.*(1.3+y1).*15]+y1.*(1+0.3.*0.6).*[35.*0.6-
y1.*15+(0.9+y1+y3).*0.6.*15]]./[4.*0.3.*(1.3+y1).*(0.9+y1+y3).*0.6-
y1.*y1.*(1+0.3.*0.6).*(1+0.3.*0.6)]
B3=[0.6.*[(1+0.3.*0.6).*y1.*y3+2.*0.5.*(0.9+y1+y3).*0.6]]./[4.*0.3.*(1.3+y1).*(0.9
+y1+y3).*0.6-y1.*y1.*(1+0.3.*0.6).*(1+0.3.*0.6)]
C3=[35.*0.6-
y1.*15+(0.9+y1+y3).*0.6.*15+y1.*(1+0.3.*0.6).*A1]./[2.*(0.9+y1+y3).*0.6]
D3=[y1.*(1+0.3.*0.6).*B1+y3.*0.6]./[2.*(0.9+y1+y3).*0.6]
E3=15.*[(-0.3.*(1.3+y1).*B3+y1.*D3+0.5)+0.3.*y1.*B3-
(0.9+y1+y3).*D3+y3]./[2.*0.6.*[(1.4+y3)-0.3.*0.5.*B3-y3.*D3]]
F3=[35+0.3.*0.5.*A3+y3.*C3]./[2.*[(1.4+y3)-0.3.*0.5.*B3-y3.*D3]]
P2M=C3+D3.*[E3+F3]
P2R=C2+[D2.*(A2+B2.*C2)]./(1-B2.*D2)
P2N=C1+D1.*[0.6.*[35+0.3.*0.5.*A1+y3.*C1]+15.*[0.5+y3]]./[0.6.*[2.*(1.4+y3)-
0.3.*0.5.*B1-y3.*D1]]
surf(y1,y3,P2N);
hold on
surf(y1,y3,P2R);
surf(y1,y3,P2M);
xlabel('\gamma_1');
ylabel('\gamma_3');
zlabel('p_2')
legend('p_2^N', 'p_2^{RS}', 'p_2^{MS}')
hold off
```

S5 Fig 6. (C) Code

```
[y1 y3]=meshgrid(0:0.1:1,0:0.1:1);
A1=[2.*(0.9+y1+y3).*0.6.*[40-
0.3.*y1.*0.6.*15+0.3.*(1.3+y1).*15]+y1.*(1+0.3.*0.6).*[35.*0.6-
y1.*15+(0.9+y1+y3).*0.6.*15]]./[4.*0.3.*(1.3+y1).*(0.9+y1+y3).*0.6-
y1.*y1.*(1+0.3.*0.6).*(1+0.3.*0.6)]
B1=[0.6.*[(1+0.3.*0.6).*y1.*y3+2.*0.5.*(0.9+y1+y3).*0.6]]./[4.*0.3.*(1.3+y1).*(0.9
+y1+y3).*0.6-y1.*y1.*(1+0.3.*0.6).*(1+0.3.*0.6)]
```

```
C1=[35.*0.6-
y1.*15+(0.9+y1+y3).*0.6.*15+y1.*(1+0.3.*0.6).*A1]./[2.*(0.9+y1+y3).*0.6]
D1=[y1.*(1+0.3.*0.6).*B1+y3.*0.6]./[2.*(0.9+y1+y3).*0.6]
A2=[0.6.*[2.*(1.4+y3).*40+15.*0.3.*[2.*(1.4+y3).*(1.3+y1)-0.5.*0.5-
0.6.*[2.*(1.4+y3).*y1+0.5.*y3]]]+0.5.*[0.6.*35+15.*(0.5+y3)]]./[2.*0.3.*0.6.*[2.*(1.
4+y3).*(1.3+y1)-0.5.*0.5]]
B2=[[1+0.3.*0.6].*[2.*(1.4+y3).*y1+y3.*0.5]]./[2.*0.3.*(2.*(1.3+y1).*(1.4+y3)-
0.5.*0.5)]
C2=[0.6.*[2.*(1.4+y3).*35+15.*[2.*(1.4+y3).*(0.9+y1+y3)-
y3.*y3]+( y3).*35]+15.*[y3.*y3-
2.*(1.4+y3).*y1]]./[2.*0.6.*[2.*(1.4+y3).*(0.9+y1+y3)-y3.*y3]]
D2=[2.*(1.4+y3).*y1+0.5.*y3+0.3.*[2.*(1.4+y3).*y1+0.5].*0.6]./[2.*0.6.*(2.*(1.4+y
3).*(0.9+y1+y3)-y3.*y3)]
E2=[0.3.*0.5./(2.*(1.4+y3))].*[A2+B2.*C2]./[1-
B2.*D2]+y3.*[C2+[D2.*(A2+B2.*C2)./(1-B2.*D2)]]./(2.*(1.4+y3))
A3=[2.*(0.9+y1+y3).*0.6.*[40-
0.3.*y1.*0.6.*15+0.3.*(1.3+y1).*15]+y1.*(1+0.3.*0.6).*[35.*0.6-
y1.*15+(0.9+y1+y3).*0.6.*15]]./[4.*0.3.*(1.3+y1).*(0.9+y1+y3).*0.6-
y1.*y1.*(1+0.3.*0.6).*(1+0.3.*0.6)]
B3=[0.6.*[(1+0.3.*0.6).*y1.*y3+2.*0.5.*(0.9+y1+y3).*0.6]]./[4.*0.3.*(1.3+y1).*(0.9
+y1+y3).*0.6-y1.*y1.*(1+0.3.*0.6).*(1+0.3.*0.6)]
C3=[35.*0.6-
y1.*15+(0.9+y1+y3).*0.6.*15+y1.*(1+0.3.*0.6).*A1]./[2.*(0.9+y1+y3).*0.6]
D3=[y1.*(1+0.3.*0.6).*B1+y3.*0.6]./[2.*(0.9+y1+y3).*0.6]
E3=15.*[(-0.3.*(1.3+y1).*B3+y1.*D3+0.5)+0.3.*y1.*B3-
(0.9+y1+y3).*D3+y3]./[2.*0.6.*[(1.4+y3)-0.3.*0.5.*B3-y3.*D3]]
F3=[35+0.3.*0.5.*A3+y3.*C3]./[2.*[(1.4+y3)-0.3.*0.5.*B3-y3.*D3]]
P3N=[0.6.*[35+0.3.*0.5.*A1+y3.*C1]+15.*[0.5+y3]]./[0.6.*[2.*(1.4+y3)-
0.3.*0.5.*B1-y3.*D1]]
P3R=E2+[0.6.*35+15.*(0.5+y3)]./(2.*(1.4+y3).*0.6)
P3M=E3 +F3
surf(y1,y3,P3N);
hold on
surf(y1,y3,P3R);
surf(y1,y3,P3M);
xlabel('\gamma_1');
ylabel('\gamma_3');
zlabel('p_3')
legend('p_3^N', 'p_3^{RS}', 'p_3^{MS}')
hold off
```

S5 Fig 6. (D) Code

```
[y1 y3]=meshgrid(0:0.1:1,0:0.1:1);
A1=[2.*(0.9+y1+y3).*0.6.*[40-
0.3.*y1.*0.6.*15+0.3.*(1.3+y1).*15]+y1.*(1+0.3.*0.6).*[35.*0.6-
y1.*15+(0.9+y1+y3).*0.6.*15]]./[4.*0.3.*(1.3+y1).*(0.9+y1+y3).*0.6-
y1.*y1.*(1+0.3.*0.6).*(1+0.3.*0.6)]
```

B1=[0.6.*[(1+0.3.*0.6).*y1.*y3+2.*0.5.*(0.9+y1+y3).*0.6]]./[4.*0.3.*(1.3+y1).*(0.9+y1+y3).*0.6-y1.*y1.*(1+0.3.*0.6).*(1+0.3.*0.6)]
C1=[35.*0.6-y1.*15+(0.9+y1+y3).*0.6.*15+y1.*(1+0.3.*0.6).*A1]./[2.*(0.9+y1+y3).*0.6]
D1=[y1.*(1+0.3.*0.6).*B1+y3.*0.6]./[2.*(0.9+y1+y3).*0.6]
A2=[0.6.*[2.*(1.4+y3).*40+15.*0.3.*[2.*(1.4+y3).*(1.3+y1)-0.5.*0.5-0.6.*[2.*(1.4+y3).*y1+0.5.*y3]]]+0.5.*[0.6.*35+15.*(0.5+y3)]]./[2.*0.3.*0.6.*[2.*(1.4+y3).*(1.3+y1)-0.5.*0.5]]
B2=[[1+0.3.*0.6].*[2.*(1.4+y3).*y1+y3.*0.5]]./[2.*0.3.*(2.*(1.3+y1).*(1.4+y3)-0.5.*0.5)]
C2=[0.6.*[2.*(1.4+y3).*35+15.*[2.*(1.4+y3).*(0.9+y1+y3)-y3.*y3]+(y3).*35]+15.*[y3.*y3-2.*(1.4+y3).*y1]]./[2.*0.6.*[2.*(1.4+y3).*(0.9+y1+y3)-y3.*y3]]
D2=[2.*(1.4+y3).*y1+0.5.*y3+0.3.*[2.*(1.4+y3).*y1+0.5].*0.6]./[2.*0.6.*(2.*(1.4+y3).*(0.9+y1+y3)-y3.*y3)]
E2=[0.3.*0.5./(2.*(1.4+y3))].*[A2+B2.*C2]./[1-B2.*D2]+y3.*[C2+[D2.*(A2+B2.*C2)./(1-B2.*D2)]]./(2.*(1.4+y3))
A3=[2.*(0.9+y1+y3).*0.6.*[40-0.3.*y1.*0.6.*15+0.3.*(1.3+y1).*15]+y1.*(1+0.3.*0.6).*[35.*0.6-y1.*15+(0.9+y1+y3).*0.6.*15]]./[4.*0.3.*(1.3+y1).*(0.9+y1+y3).*0.6-y1.*y1.*(1+0.3.*0.6).*(1+0.3.*0.6)]
B3=[0.6.*[(1+0.3.*0.6).*y1.*y3+2.*0.5.*(0.9+y1+y3).*0.6]]./[4.*0.3.*(1.3+y1).*(0.9+y1+y3).*0.6-y1.*y1.*(1+0.3.*0.6).*(1+0.3.*0.6)]
C3=[35.*0.6-y1.*15+(0.9+y1+y3).*0.6.*15+y1.*(1+0.3.*0.6).*A1]./[2.*(0.9+y1+y3).*0.6]
D3=[y1.*(1+0.3.*0.6).*B1+y3.*0.6]./[2.*(0.9+y1+y3).*0.6]
E3=15.*[(-0.3.*(1.3+y1).*B3+y1.*D3+0.5)+0.3.*y1.*B3-(0.9+y1+y3).*D3+y3]./[2.*0.6.*[(1.4+y3)-0.3.*0.5.*B3-y3.*D3]]
F3=[35+0.3.*0.5.*A3+y3.*C3]./[2.*[(1.4+y3)-0.3.*0.5.*B3-y3.*D3]]
P1N=A1+B1.*[0.6.*[35+0.3.*0.5.*A1+y3.*C1]+15.*[0.5+y3]]./[0.6.*[2.*(1.4+y3)-0.3.*0.5.*B1-y3.*D1]]
P1R=(A2+B2.*C2)./(1-B2.*D2)
P1M=A3+B3.*[E3+F3]
P2M=C3+D3.*[E3+F3]
P2R=C2+[D2.*(A2+B2.*C2)]./(1-B2.*D2)
P2N=C1+D1.*[0.6.*[35+0.3.*0.5.*A1+y3.*C1]+15.*[0.5+y3]]./[0.6.*[2.*(1.4+y3)-0.3.*0.5.*B1-y3.*D1]]
P3N=[0.6.*[35+0.3.*0.5.*A1+y3.*C1]+15.*[0.5+y3]]./[0.6.*[2.*(1.4+y3)-0.3.*0.5.*B1-y3.*D1]]
P3R=E2+[0.6.*35+15.*(0.5+y3)]./(2.*(1.4+y3).*0.6)
P3M=E3 +F3
Q1N=40-0.3.*(1.3+y1).*P1N+y1.*P2N+0.5.*P3N
Q2N=35-(0.9+y1+y3).*P2N+0.3.*y1.*P1N+y3.*P3N
Q3N=35-(1.4+y3).*P3N+0.3.*0.5.*P1N+y3.*P2N
Q1R=40-0.3.*(1.3+y1).*P1R+y1.*P2R+0.5.*P3R
Q2R=35-(0.9+y1+y3).*P2R+0.3.*y1.*P1R+y3.*P3R
Q3R=35-(1.4+y3).*P3R+0.3.*0.5.*P1R+y3.*P2R
Q1M=40-0.3.*(1.3+y1).*P1M+y1.*P2M+0.5.*P3M
Q2M=35-(0.9+y1+y3).*P2M+0.3.*y1.*P1M+y3.*P3M
Q3M=35-(1.4+y3).*P3M+0.3.*0.5.*P1M+y3.*P2M

```
V1R=(P1N-15).*Q1N+0.6.*(P2N-15).*Q2N
V2R=(P1R-15).*Q1R+0.6.*(P2R-15).*Q2R
V3R=(P1M-15).*Q1M+0.6.*(P2M-15).*Q2M
surf(y1,y3,V1R);
hold on
surf(y1,y3,V2R);
surf(y1,y3,V3R);
xlabel('\gamma_1');
ylabel('\gamma_3');
zlabel(' V_R')
legend('V_R^N', 'V_R^{RS}', 'V_R^{MS}')
hold off
```

S5 Fig 6. (E) Code

```
[y1 y3]=meshgrid(0:0.1:1,0:0.1:1);
A1=[2.*(0.9+y1+y3).*0.6.*[40-
0.3.*y1.*0.6.*15+0.3.*(1.3+y1).*15]+y1.*(1+0.3.*0.6).*[35.*0.6-
y1.*15+(0.9+y1+y3).*0.6.*15]]./[4.*0.3.*(1.3+y1).*(0.9+y1+y3).*0.6-
y1.*y1.*(1+0.3.*0.6).*(1+0.3.*0.6)]
B1=[0.6.*[(1+0.3.*0.6).*y1.*y3+2.*0.5.*(0.9+y1+y3).*0.6]]./[4.*0.3.*(1.3+y1).*(0.9
+y1+y3).*0.6-y1.*y1.*(1+0.3.*0.6).*(1+0.3.*0.6)]
C1=[35.*0.6-
y1.*15+(0.9+y1+y3).*0.6.*15+y1.*(1+0.3.*0.6).*A1]./[2.*(0.9+y1+y3).*0.6]
D1=[y1.*(1+0.3.*0.6).*B1+y3.*0.6]./[2.*(0.9+y1+y3).*0.6]
A2=[0.6.*[2.*(1.4+y3).*40+15.*0.3.*[2.*(1.4+y3).*(1.3+y1)-0.5.*0.5-
0.6.*[2.*(1.4+y3).*y1+0.5.*y3]]]+0.5.*[0.6.*35+15.*(0.5+y3)]]./[2.*0.3.*0.6.*[2.*(1.
4+y3).*(1.3+y1)-0.5.*0.5]]
B2=[[1+0.3.*0.6].*[2.*(1.4+y3).*y1+y3.*0.5]]./[2.*0.3.*(2.*(1.3+y1).*(1.4+y3)-
0.5.*0.5)]
C2=[0.6.*[2.*(1.4+y3).*35+15.*[2.*(1.4+y3).*(0.9+y1+y3)-
y3.*y3]+( y3).*35]+15.*[y3.*y3-
2.*(1.4+y3).*y1]]./[2.*0.6.*[2.*(1.4+y3).*(0.9+y1+y3)-y3.*y3]]
D2=[2.*(1.4+y3).*y1+0.5.*y3+0.3.*[2.*(1.4+y3).*y1+0.5].*0.6]./[2.*0.6.*(2.*(1.4+y
3).*(0.9+y1+y3)-y3.*y3)]
E2=[0.3.*0.5./(2.*(1.4+y3))].*[A2+B2.*C2]./[1-
B2.*D2]+y3.*[C2+[D2.*(A2+B2.*C2)./(1-B2.*D2)]]./(2.*(1.4+y3))
A3=[2.*(0.9+y1+y3).*0.6.*[40-
0.3.*y1.*0.6.*15+0.3.*(1.3+y1).*15]+y1.*(1+0.3.*0.6).*[35.*0.6-
y1.*15+(0.9+y1+y3).*0.6.*15]]./[4.*0.3.*(1.3+y1).*(0.9+y1+y3).*0.6-
y1.*y1.*(1+0.3.*0.6).*(1+0.3.*0.6)]
B3=[0.6.*[(1+0.3.*0.6).*y1.*y3+2.*0.5.*(0.9+y1+y3).*0.6]]./[4.*0.3.*(1.3+y1).*(0.9
+y1+y3).*0.6-y1.*y1.*(1+0.3.*0.6).*(1+0.3.*0.6)]
C3=[35.*0.6-
y1.*15+(0.9+y1+y3).*0.6.*15+y1.*(1+0.3.*0.6).*A1]./[2.*(0.9+y1+y3).*0.6]
D3=[y1.*(1+0.3.*0.6).*B1+y3.*0.6]./[2.*(0.9+y1+y3).*0.6]
E3=15.*[(-0.3.*(1.3+y1).*B3+y1.*D3+0.5)+0.3.*y1.*B3-
(0.9+y1+y3).*D3+y3]./[2.*0.6.*[(1.4+y3)-0.3.*0.5.*B3-y3.*D3]]
```

```
F3=[35+0.3.*0.5.*A3+y3.*C3]./[2.*[(1.4+y3)-0.3.*0.5.*B3-y3.*D3]]
P1N=A1+B1.*[0.6.*[35+0.3.*0.5.*A1+y3.*C1]+15.*[0.5+y3]]./[0.6.*[2.*(1.4+y3)-
0.3.*0.5.*B1-y3.*D1]]
P1R=(A2+B2.*C2)./(1-B2.*D2)
P1M=A3+B3.*[E3+F3]
P2M=C3+D3.*[E3+F3]
P2R=C2+[D2.*(A2+B2.*C2)]./(1-B2.*D2)
P2N=C1+D1.*[0.6.*[35+0.3.*0.5.*A1+y3.*C1]+15.*[0.5+y3]]./[0.6.*[2.*(1.4+y3)-
0.3.*0.5.*B1-y3.*D1]]
P3N=[0.6.*[35+0.3.*0.5.*A1+y3.*C1]+15.*[0.5+y3]]./[0.6.*[2.*(1.4+y3)-
0.3.*0.5.*B1-y3.*D1]]
P3R=E2+[0.6.*35+15.*(0.5+y3)]./(2.*(1.4+y3).*0.6)
P3M=E3 +F3
Q1N=40-0.3.*(1.3+y1).*P1N+y1.*P2N+0.5.*P3N
Q2N=35-(0.9+y1+y3).*P2N+0.3.*y1.*P1N+y3.*P3N
Q3N=35-(1.4+y3).*P3N+0.3.*0.5.*P1N+y3.*P2N
Q1R=40-0.3.*(1.3+y1).*P1R+y1.*P2R+0.5.*P3R
Q2R=35-(0.9+y1+y3).*P2R+0.3.*y1.*P1R+y3.*P3R
Q3R=35-(1.4+y3).*P3R+0.3.*0.5.*P1R+y3.*P2R
Q1M=40-0.3.*(1.3+y1).*P1M+y1.*P2M+0.5.*P3M
Q2M=35-(0.9+y1+y3).*P2M+0.3.*y1.*P1M+y3.*P3M
Q3M=35-(1.4+y3).*P3M+0.3.*0.5.*P1M+y3.*P2M
V1M=0.6.*P3N.*Q3N+15.*(Q1N+Q2N)
V2M=0.6.*P3R.*Q3R+15.*(Q1R+Q2R)
V3M=0.6.*P3M.*Q3M+15.*(Q1M+Q2M)
surf(y1,y3,V1M);
hold on
surf(y1,y3,V2M);
surf(y1,y3,V3M);
xlabel('\gamma_1');
ylabel('\gamma_3');
zlabel(' V_M')
legend('V_M^N', 'V_M^{RS}', 'V_M^{MS}')
hold off
```

S5 Fig 6. (F) Code

```
[y1 y3]=meshgrid(0:0.1:1,0:0.1:1);
A1=[2.*(0.9+y1+y3).*0.6.*[40-
0.3.*y1.*0.6.*15+0.3.*(1.3+y1).*15]+y1.*(1+0.3.*0.6).*[35.*0.6-
y1.*15+(0.9+y1+y3).*0.6.*15]]./[4.*0.3.*(1.3+y1).*(0.9+y1+y3).*0.6-
y1.*y1.*(1+0.3.*0.6).*(1+0.3.*0.6)]
B1=[0.6.*[(1+0.3.*0.6).*y1.*y3+2.*0.5.*(0.9+y1+y3).*0.6]]./[4.*0.3.*(1.3+y1).*(0.9
+y1+y3).*0.6-y1.*y1.*(1+0.3.*0.6).*(1+0.3.*0.6)]
C1=[35.*0.6-
y1.*15+(0.9+y1+y3).*0.6.*15+y1.*(1+0.3.*0.6).*A1]./[2.*(0.9+y1+y3).*0.6]
D1=[y1.*(1+0.3.*0.6).*B1+y3.*0.6]./[2.*(0.9+y1+y3).*0.6]
A2=[0.6.*[2.*(1.4+y3).*40+15.*0.3.*[2.*(1.4+y3).*(1.3+y1)-0.5.*0.5-
```

0.6.*[2.*(1.4+y3).*y1+0.5.*y3]]]+0.5.*[0.6.*35+15.*(0.5+y3)]]./[2.*0.3.*0.6.*[2.*(1.4+y3).*(1.3+y1)-0.5.*0.5]]
B2=[[1+0.3.*0.6].*[2.*(1.4+y3).*y1+y3.*0.5]]./[2.*0.3.*(2.*(1.3+y1).*(1.4+y3)-0.5.*0.5)]
C2=[0.6.*[2.*(1.4+y3).*35+15.*[2.*(1.4+y3).*(0.9+y1+y3)-y3.*y3]+(y3).*35]+15.*[y3.*y3-2.*(1.4+y3).*y1]]./[2.*0.6.*[2.*(1.4+y3).*(0.9+y1+y3)-y3.*y3]]
D2=[2.*(1.4+y3).*y1+0.5.*y3+0.3.*[2.*(1.4+y3).*y1+0.5].*0.6]./[2.*0.6.*(2.*(1.4+y3).*(0.9+y1+y3)-y3.*y3)]
E2=[0.3.*0.5./(2.*(1.4+y3))].*[A2+B2.*C2]./[1-B2.*D2]+y3.*[C2+[D2.*(A2+B2.*C2)./(1-B2.*D2)]]./(2.*(1.4+y3))
A3=[2.*(0.9+y1+y3).*0.6.*[40-0.3.*y1.*0.6.*15+0.3.*(1.3+y1).*15]+y1.*(1+0.3.*0.6).*[35.*0.6-y1.*15+(0.9+y1+y3).*0.6.*15]]./[4.*0.3.*(1.3+y1).*(0.9+y1+y3).*0.6-y1.*y1.*(1+0.3.*0.6).*(1+0.3.*0.6)]
B3=[0.6.*[(1+0.3.*0.6).*y1.*y3+2.*0.5.*(0.9+y1+y3).*0.6]]./[4.*0.3.*(1.3+y1).*(0.9+y1+y3).*0.6-y1.*y1.*(1+0.3.*0.6).*(1+0.3.*0.6)]
C3=[35.*0.6-y1.*15+(0.9+y1+y3).*0.6.*15+y1.*(1+0.3.*0.6).*A1]./[2.*(0.9+y1+y3).*0.6]
D3=[y1.*(1+0.3.*0.6).*B1+y3.*0.6]./[2.*(0.9+y1+y3).*0.6]
E3=15.*[(-0.3.*(1.3+y1).*B3+y1.*D3+0.5)+0.3.*y1.*B3-(0.9+y1+y3).*D3+y3]./[2.*0.6.*[(1.4+y3)-0.3.*0.5.*B3-y3.*D3]]
F3=[35+0.3.*0.5.*A3+y3.*C3]./[2.*[(1.4+y3)-0.3.*0.5.*B3-y3.*D3]]
P1N=A1+B1.*[0.6.*[35+0.3.*0.5.*A1+y3.*C1]+15.*[0.5+y3]]./[0.6.*[2.*(1.4+y3)-0.3.*0.5.*B1-y3.*D1]]
P1R=(A2+B2.*C2)./(1-B2.*D2)
P1M=A3+B3.*[E3+F3]
P2M=C3+D3.*[E3+F3]
P2R=C2+[D2.*(A2+B2.*C2)]./(1-B2.*D2)
P2N=C1+D1.*[0.6.*[35+0.3.*0.5.*A1+y3.*C1]+15.*[0.5+y3]]./[0.6.*[2.*(1.4+y3)-0.3.*0.5.*B1-y3.*D1]]
P3N=[0.6.*[35+0.3.*0.5.*A1+y3.*C1]+15.*[0.5+y3]]./[0.6.*[2.*(1.4+y3)-0.3.*0.5.*B1-y3.*D1]]
P3R=E2+[0.6.*35+15.*(0.5+y3)]./(2.*(1.4+y3).*0.6)
P3M=E3 +F3
Q1N=40-0.3.*(1.3+y1).*P1N+y1.*P2N+0.5.*P3N
Q2N=35-(0.9+y1+y3).*P2N+0.3.*y1.*P1N+y3.*P3N
Q3N=35-(1.4+y3).*P3N+0.3.*0.5.*P1N+y3.*P2N
Q1R=40-0.3.*(1.3+y1).*P1R+y1.*P2R+0.5.*P3R
Q2R=35-(0.9+y1+y3).*P2R+0.3.*y1.*P1R+y3.*P3R
Q3R=35-(1.4+y3).*P3R+0.3.*0.5.*P1R+y3.*P2R
Q1M=40-0.3.*(1.3+y1).*P1M+y1.*P2M+0.5.*P3M
Q2M=35-(0.9+y1+y3).*P2M+0.3.*y1.*P1M+y3.*P3M
Q3M=35-(1.4+y3).*P3M+0.3.*0.5.*P1M+y3.*P2M
V1R=(P1N-15).*Q1N+0.6.*(P2N-15).*Q2N
V2R=(P1R-15).*Q1R+0.6.*(P2R-15).*Q2R
V3R=(P1M-15).*Q1M+0.6.*(P2M-15).*Q2M
V1M=0.6.*P3N.*Q3N+15.*(Q1N+Q2N)
V2M=0.6.*P3R.*Q3R+15.*(Q1R+Q2R)
V3M=0.6.*P3M.*Q3M+15.*(Q1M+Q2M)

```
V1=V1R+V1M
V2=V2R+V2M
V3=V3R+V3M
surf(y1,y3,V1);
hold on
surf(y1,y3,V2);
surf(y1,y3,V3);
xlabel('\gamma_1');
ylabel('\gamma_3');
zlabel('V')
legend('V^N', 'V^{RS}', 'V^{MS}')
hold off
```

S6 Fig 7. (A) Code

```
[y2 y3]=meshgrid(0:0.1:1,0:0.1:1);
A1=[2.*(1.4+y3).*0.6.*[40-
0.3.*0.5.*0.6.*15+0.3.*(1.3+y2).*15]+0.5.*(1+0.3.*0.6).*[35.*0.6-
0.5.*15+(1.4+y3).*0.6.*15]]./[4.*0.3.*(1.3+y2).*(1.4+y3).*0.6-
0.5.*0.5.*(1+0.3.*0.6).*(1+0.3.*0.6)]
B1=[0.6.*[(1+0.3.*0.6).*0.5.*y3+2.*y2.*(1.4+y3).*0.6]]./[4.*0.3.*(1.3+y2).*(1.4+y3
).*0.6-0.5.*0.5.*(1+0.3.*0.6).*(1+0.3.*0.6)]
C1=[35.*0.6-0.5.*15+(1.4+y3).*0.6.*15+0.5.*(1+0.3.*0.6).*A1]./[2.*(1.4+y3).*0.6]
D1=[0.5.*(1+0.3.*0.6).*B1+y3.*0.6]./[2.*(1.4+y3).*0.6]
A2=[0.6.*[2.*(0.9+y3+y2).*40+15.*0.3.*[2.*(0.9+y3+y2).*(1.3+y2)-y2.*y2-
0.6.*[2.*(0.9+y3+y2).*0.5+y2.*y3]]]+y2.*[0.6.*35+15.*( y3+y2)]]./[2.*0.3.*0.6.*[2.
*(0.9+y3+y2).*(1.3+y2)-y2.*y2]]
B2=[[1+0.3.*0.6].*[2.*(0.9+y3+y2).*0.5+y2.*y3]]./[2.*0.3.*(2.*(1.3+y2).*(0.9+y3+y
2)-y2.*y2)]
C2=[0.6.*[2.*(0.9+y3+y2).*35+15.*[2.*(0.9+y3+y2).*(1.4+y3)-
y3.*y3]+y3.*35]+15.*[y3.*y3-
2.*(0.9+y3+y2).*0.5]]./[2.*0.6.*[2.*(0.9+y3+y2).*(1.4+y3)-y3.*y3]]
D2=[2.*(0.9+y3+y2).*0.5+y2.*y3+0.3.*[2.*(0.9+y3+y2).*0.5+y2].*0.6]./[2.*0.6.*(2.
*(0.9+y3+y2).*(1.4+y3)-y3.*y3)]
E2=[0.3.*y2./(2.*(0.9+y3+y2))].*[A2+B2.*C2]./[1-
B2.*D2]+y3.*[C2+[D2.*(A2+B2.*C2)./(1-B2.*D2)]]./(2.*(0.9+y3+y2))
A3=[2.*(1.4+y3).*0.6.*[40-
0.3.*0.5.*0.6.*15+0.3.*(1.3+y2).*15]+0.5.*(1+0.3.*0.6).*[35.*0.6-
0.5.*15+(1.4+y3).*0.6.*15]]./[4.*0.3.*(1.3+y2).*(1.4+y3).*0.6-
0.5.*0.5.*(1+0.3.*0.6).*(1+0.3.*0.6)]
B3=[0.6.*[(1+0.3.*0.6).*0.5.*y3+2.*y2.*(1.4+y3).*0.6]]./[4.*0.3.*(1.3+y2).*(1.4+y3
).*0.6-0.5.*0.5.*(1+0.3.*0.6).*(1+0.3.*0.6)]
C3=[35.*0.6-0.5.*15+(1.4+y3).*0.6.*15+0.5.*(1+0.3.*0.6).*A3]./[2.*(1.4+y3).*0.6]
D3=[0.5.*(1+0.3.*0.6).*B3+y3.*0.6]./[2.*(1.4+y3).*0.6]
E3=15.*[-0.3.*(1.3+y2).*B3+0.5.*D3+y2+0.3.*0.5.*B3-
(1.4+y3).*D3+y3]./[2.*0.6.*[(0.9+y3+y2)-0.3.*y2.*B3-y3.*D3]]
F3=[35+0.3.*y2.*A3+y3.*C3]./[2.*[0.9+y3+y2-0.3.*y2.*B3-y3.*D3]]
```

```
P1N=A1+B1.*[0.6.*[35+0.3.*y2.*A1+y3.*C1]+15.*[y2+y3]]./[0.6.*[2.*(0.9+y3+y2)
-0.3.*y2.*B1-y3.*D1]]
P1R=(A2+B2.*C2)./(1-B2.*D2)
P1M=A3+B3.*[E3+F3]
surf(y2,y3,P1N);
hold on
surf(y2,y3,P1R);
surf(y2,y3,P1M);
xlabel('\gamma_2');
ylabel('\gamma_3');
zlabel('p_1')
legend('p_1^N', 'p_1^{RS}', 'p_1^{MS}')
hold off
```

S6 Fig 7. (B) Code

```
[y2 y3]=meshgrid(0:0.1:1,0:0.1:1);
A1=[2.*(1.4+y3).*0.6.*[40-
0.3.*0.5.*0.6.*15+0.3.*(1.3+y2).*15]+0.5.*(1+0.3.*0.6).*[35.*0.6-
0.5.*15+(1.4+y3).*0.6.*15]]./[4.*0.3.*(1.3+y2).*(1.4+y3).*0.6-
0.5.*0.5.*(1+0.3.*0.6).*(1+0.3.*0.6)]
B1=[0.6.*[(1+0.3.*0.6).*0.5.*y3+2.*y2.*(1.4+y3).*0.6]]./[4.*0.3.*(1.3+y2).*(1.4+y3
).*0.6-0.5.*0.5.*(1+0.3.*0.6).*(1+0.3.*0.6)]
C1=[35.*0.6-0.5.*15+(1.4+y3).*0.6.*15+0.5.*(1+0.3.*0.6).*A1]./[2.*(1.4+y3).*0.6]
D1=[0.5.*(1+0.3.*0.6).*B1+y3.*0.6]./[2.*(1.4+y3).*0.6]
A2=[0.6.*[2.*(0.9+y3+y2).*40+15.*0.3.*[2.*(0.9+y3+y2).*(1.3+y2)-y2.*y2-
0.6.*[2.*(0.9+y3+y2).*0.5+y2.*y3]]]+y2.*[0.6.*35+15.*( y3+y2)]]./[2.*0.3.*0.6.*[2.
*(0.9+y3+y2).*(1.3+y2)-y2.*y2]]
B2=[[1+0.3.*0.6].*[2.*(0.9+y3+y2).*0.5+y2.*y3]]./[2.*0.3.*(2.*(1.3+y2).*(0.9+y3+y
2)-y2.*y2)]
C2=[0.6.*[2.*(0.9+y3+y2).*35+15.*[2.*(0.9+y3+y2).*(1.4+y3)-
y3.*y3]+y3.*35]+15.*[y3.*y3-
2.*(0.9+y3+y2).*0.5]]./[2.*0.6.*[2.*(0.9+y3+y2).*(1.4+y3)-y3.*y3]]
D2=[2.*(0.9+y3+y2).*0.5+y2.*y3+0.3.*[2.*(0.9+y3+y2).*0.5+y2].*0.6]./[2.*0.6.*(2.
*(0.9+y3+y2).*(1.4+y3)-y3.*y3)]
E2=[0.3.*y2./(2.*(0.9+y3+y2))].*[A2+B2.*C2]./[1-
B2.*D2]+y3.*[C2+[D2.*(A2+B2.*C2)./(1-B2.*D2)]]./(2.*(0.9+y3+y2))
A3=[2.*(1.4+y3).*0.6.*[40-
0.3.*0.5.*0.6.*15+0.3.*(1.3+y2).*15]+0.5.*(1+0.3.*0.6).*[35.*0.6-
0.5.*15+(1.4+y3).*0.6.*15]]./[4.*0.3.*(1.3+y2).*(1.4+y3).*0.6-
0.5.*0.5.*(1+0.3.*0.6).*(1+0.3.*0.6)]
B3=[0.6.*[(1+0.3.*0.6).*0.5.*y3+2.*y2.*(1.4+y3).*0.6]]./[4.*0.3.*(1.3+y2).*(1.4+y3
).*0.6-0.5.*0.5.*(1+0.3.*0.6).*(1+0.3.*0.6)]
C3=[35.*0.6-0.5.*15+(1.4+y3).*0.6.*15+0.5.*(1+0.3.*0.6).*A3]./[2.*(1.4+y3).*0.6]
D3=[0.5.*(1+0.3.*0.6).*B3+y3.*0.6]./[2.*(1.4+y3).*0.6]
E3=15.*[-0.3.*(1.3+y2).*B3+0.5.*D3+y2+0.3.*0.5.*B3-
(1.4+y3).*D3+y3]./[2.*0.6.*[(0.9+y3+y2)-0.3.*y2.*B3-y3.*D3]]
F3=[35+0.3.*y2.*A3+y3.*C3]./[2.*[0.9+y3+y2-0.3.*y2.*B3-y3.*D3]]
```

```
P2M=C3+D3.*[E3+F3]
P2R=C2+[D2.*(A2+B2.*C2)]./(1-B2.*D2)
P2N=C1+D1.*[0.6.*[35+0.3.*y2.*A1+y3.*C1]+15.*[y2+y3]]./[0.6.*[2.*(0.9+y3+y2)
-0.3.*y2.*B1-y3.*D1]]
surf(y2,y3,P2N);
hold on
surf(y2,y3,P2R);
surf(y2,y3,P2M);
xlabel('\gamma_2');
ylabel('\gamma_3');
zlabel('p_2')
legend('p_2^N', 'p_2^{RS}', 'p_2^{MS}')
hold off
```

S6 Fig 7. (C) Code

```
[y2 y3]=meshgrid(0:0.1:1,0:0.1:1);
A1=[2.*(1.4+y3).*0.6.*[40-
0.3.*0.5.*0.6.*15+0.3.*(1.3+y2).*15]+0.5.*(1+0.3.*0.6).*[35.*0.6-
0.5.*15+(1.4+y3).*0.6.*15]]./[4.*0.3.*(1.3+y2).*(1.4+y3).*0.6-
0.5.*0.5.*(1+0.3.*0.6).*(1+0.3.*0.6)]
B1=[0.6.*[(1+0.3.*0.6).*0.5.*y3+2.*y2.*(1.4+y3).*0.6]]./[4.*0.3.*(1.3+y2).*(1.4+y3
).*0.6-0.5.*0.5.*(1+0.3.*0.6).*(1+0.3.*0.6)]
C1=[35.*0.6-0.5.*15+(1.4+y3).*0.6.*15+0.5.*(1+0.3.*0.6).*A1]./[2.*(1.4+y3).*0.6]
D1=[0.5.*(1+0.3.*0.6).*B1+y3.*0.6]./[2.*(1.4+y3).*0.6]
A2=[0.6.*[2.*(0.9+y3+y2).*40+15.*0.3.*[2.*(0.9+y3+y2).*(1.3+y2)-y2.*y2-
0.6.*[2.*(0.9+y3+y2).*0.5+y2.*y3]]]+y2.*[0.6.*35+15.*( y3+y2)]]./[2.*0.3.*0.6.*[2.
*(0.9+y3+y2).*(1.3+y2)-y2.*y2]]
B2=[[1+0.3.*0.6].*[2.*(0.9+y3+y2).*0.5+y2.*y3]]./[2.*0.3.*(2.*(1.3+y2).*(0.9+y3+y
2)-y2.*y2)]
C2=[0.6.*[2.*(0.9+y3+y2).*35+15.*[2.*(0.9+y3+y2).*(1.4+y3)-
y3.*y3]+y3.*35]+15.*[y3.*y3-
2.*(0.9+y3+y2).*0.5]]./[2.*0.6.*[2.*(0.9+y3+y2).*(1.4+y3)-y3.*y3]]
D2=[2.*(0.9+y3+y2).*0.5+y2.*y3+0.3.*[2.*(0.9+y3+y2).*0.5+y2].*0.6]./[2.*0.6.*(2.
*(0.9+y3+y2).*(1.4+y3)-y3.*y3)]
E2=[0.3.*y2./(2.*(0.9+y3+y2))].*[A2+B2.*C2]./[1-
B2.*D2]+y3.*[C2+[D2.*(A2+B2.*C2)./(1-B2.*D2)]]./(2.*(0.9+y3+y2))
A3=[2.*(1.4+y3).*0.6.*[40-
0.3.*0.5.*0.6.*15+0.3.*(1.3+y2).*15]+0.5.*(1+0.3.*0.6).*[35.*0.6-
0.5.*15+(1.4+y3).*0.6.*15]]./[4.*0.3.*(1.3+y2).*(1.4+y3).*0.6-
0.5.*0.5.*(1+0.3.*0.6).*(1+0.3.*0.6)]
B3=[0.6.*[(1+0.3.*0.6).*0.5.*y3+2.*y2.*(1.4+y3).*0.6]]./[4.*0.3.*(1.3+y2).*(1.4+y3
).*0.6-0.5.*0.5.*(1+0.3.*0.6).*(1+0.3.*0.6)]
C3=[35.*0.6-0.5.*15+(1.4+y3).*0.6.*15+0.5.*(1+0.3.*0.6).*A3]./[2.*(1.4+y3).*0.6]
D3=[0.5.*(1+0.3.*0.6).*B3+y3.*0.6]./[2.*(1.4+y3).*0.6]
E3=15.*[-0.3.*(1.3+y2).*B3+0.5.*D3+y2+0.3.*0.5.*B3-
(1.4+y3).*D3+y3]./[2.*0.6.*[(0.9+y3+y2)-0.3.*y2.*B3-y3.*D3]]
F3=[35+0.3.*y2.*A3+y3.*C3]./[2.*[0.9+y3+y2-0.3.*y2.*B3-y3.*D3]]
```

```
P3N=[0.6.*[35+0.3.*y2.*A1+y3.*C1]+15.*[y2+y3]]./[0.6.*[2.*(0.9+y3+y2)-
0.3.*y2.*B1-y3.*D1]]
P3R=E2+[0.6.*35+15.*( y3+y2)]./(2.*(0.9+y3+y2).*0.6)
P3M=[E3+F3]
surf(y2,y3,P3N);
hold on
surf(y2,y3,P3R);
surf(y2,y3,P3M);
xlabel('\gamma_2');
ylabel('\gamma_3');
zlabel('p_3')
legend('p_3^N', 'p_3^{RS}', 'p_3^{MS}')
hold off
```

S6 Fig 7. (D) Code

```
[y2 y3]=meshgrid(0:0.1:1,0:0.1:1);
A1=[2.*(1.4+y3).*0.6.*[40-
0.3.*0.5.*0.6.*15+0.3.*(1.3+y2).*15]+0.5.*(1+0.3.*0.6).*[35.*0.6-
0.5.*15+(1.4+y3).*0.6.*15]]./[4.*0.3.*(1.3+y2).*(1.4+y3).*0.6-
0.5.*0.5.*(1+0.3.*0.6).*(1+0.3.*0.6)]
B1=[0.6.*[(1+0.3.*0.6).*0.5.*y3+2.*y2.*(1.4+y3).*0.6]]./[4.*0.3.*(1.3+y2).*(1.4+y3
).*0.6-0.5.*0.5.*(1+0.3.*0.6).*(1+0.3.*0.6)]
C1=[35.*0.6-0.5.*15+(1.4+y3).*0.6.*15+0.5.*(1+0.3.*0.6).*A1]./[2.*(1.4+y3).*0.6]
D1=[0.5.*(1+0.3.*0.6).*B1+y3.*0.6]./[2.*(1.4+y3).*0.6]
A2=[0.6.*[2.*(0.9+y3+y2).*40+15.*0.3.*[2.*(0.9+y3+y2).*(1.3+y2)-y2.*y2-
0.6.*[2.*(0.9+y3+y2).*0.5+y2.*y3]]]+y2.*[0.6.*35+15.*( y3+y2)]]./[2.*0.3.*0.6.*[2.
*(0.9+y3+y2).*(1.3+y2)-y2.*y2]]
B2=[[1+0.3.*0.6].*[2.*(0.9+y3+y2).*0.5+y2.*y3]]./[2.*0.3.*(2.*(1.3+y2).*(0.9+y3+y
2)-y2.*y2)]
C2=[0.6.*[2.*(0.9+y3+y2).*35+15.*[2.*(0.9+y3+y2).*(1.4+y3)-
y3.*y3]+y3.*35]+15.*[y3.*y3-
2.*(0.9+y3+y2).*0.5]]./[2.*0.6.*[2.*(0.9+y3+y2).*(1.4+y3)-y3.*y3]]
D2=[2.*(0.9+y3+y2).*0.5+y2.*y3+0.3.*[2.*(0.9+y3+y2).*0.5+y2].*0.6]./[2.*0.6.*(2.
*(0.9+y3+y2).*(1.4+y3)-y3.*y3)]
E2=[0.3.*y2./(2.*(0.9+y3+y2))].*[A2+B2.*C2]./[1-
B2.*D2]+y3.*[C2+[D2.*(A2+B2.*C2)./(1-B2.*D2)]]./(2.*(0.9+y3+y2))
A3=[2.*(1.4+y3).*0.6.*[40-
0.3.*0.5.*0.6.*15+0.3.*(1.3+y2).*15]+0.5.*(1+0.3.*0.6).*[35.*0.6-
0.5.*15+(1.4+y3).*0.6.*15]]./[4.*0.3.*(1.3+y2).*(1.4+y3).*0.6-
0.5.*0.5.*(1+0.3.*0.6).*(1+0.3.*0.6)]
B3=[0.6.*[(1+0.3.*0.6).*0.5.*y3+2.*y2.*(1.4+y3).*0.6]]./[4.*0.3.*(1.3+y2).*(1.4+y3
).*0.6-0.5.*0.5.*(1+0.3.*0.6).*(1+0.3.*0.6)]
C3=[35.*0.6-0.5.*15+(1.4+y3).*0.6.*15+0.5.*(1+0.3.*0.6).*A3]./[2.*(1.4+y3).*0.6]
D3=[0.5.*(1+0.3.*0.6).*B3+y3.*0.6]./[2.*(1.4+y3).*0.6]
E3=15.*[-0.3.*(1.3+y2).*B3+0.5.*D3+y2+0.3.*0.5.*B3-
(1.4+y3).*D3+y3]./[2.*0.6.*[(0.9+y3+y2)-0.3.*y2.*B3-y3.*D3]]
F3=[35+0.3.*y2.*A3+y3.*C3]./[2.*[0.9+y3+y2-0.3.*y2.*B3-y3.*D3]]
```

```
P1N=A1+B1.*[0.6.*[35+0.3.*y2.*A1+y3.*C1]+15.*[y2+y3]]./[0.6.*[2.*(0.9+y3+y2)
-0.3.*y2.*B1-y3.*D1]]
P1R=(A2+B2.*C2)./(1-B2.*D2)
P1M=A3+B3.*[E3+F3]
P2M=C3+D3.*[E3+F3]
P2R=C2+[D2.*(A2+B2.*C2)]./(1-B2.*D2)
P2N=C1+D1.*[0.6.*[35+0.3.*y2.*A1+y3.*C1]+15.*[y2+y3]]./[0.6.*[2.*(0.9+y3+y2)
-0.3.*y2.*B1-y3.*D1]]
P3N=[0.6.*[35+0.3.*y2.*A1+y3.*C1]+15.*[y2+y3]]./[0.6.*[2.*(0.9+y3+y2)-
0.3.*y2.*B1-y3.*D1]]
P3R=E2+[0.6.*35+15.*( y3+y2)]./(2.*(0.9+y3+y2).*0.6)
P3M=[E3+F3]
Q1N=40-0.3.*(1.3+y2).*P1N+0.5.*P2N+y2.*P3N
Q2N=35-(1.4+y3).*P2N+0.3.*0.5.*P1N+y3.*P3N
Q3N=35-(0.9+y3+y2).*P3N+0.3.*y2.*P1N+y3.*P2N
Q1R=40-0.3.*(1.3+y2).*P1R+0.5.*P2R+y2.*P3R
Q2R=35-(1.4+y3).*P2R+0.3.*0.5.*P1R+y3.*P3R
Q3R=35-(0.9+y3+y2).*P3R+0.3.*y2.*P1R+y3.*P2R
Q1M=40-0.3.*(1.3+y2).*P1M+0.5.*P2M+y2.*P3M
Q2M=35-(1.4+y3).*P2M+0.3.*0.5.*P1M+y3.*P3M
Q3M=35-(0.9+y3+y2).*P3M+0.3.*y2.*P1M+y3.*P2M
V1R=(P1N-15).*Q1N+0.6.*(P2N-15).*Q2N
V2R=(P1R-15).*Q1R+0.6.*(P2R-15).*Q2R
V3R=(P1M-15).*Q1M+0.6.*(P2M-15).*Q2M
surf(y2,y3,V1R);
hold on
surf(y2,y3,V2R);
surf(y2,y3,V3R);
xlabel('\gamma_2');
ylabel('\gamma_3');
zlabel(' V_R')
legend('V_R ^N', 'V_R ^{RS}', 'V_R ^{MS}')
hold off
```

S6 Fig 7. (E) Code

```
[y2 y3]=meshgrid(0:0.1:1,0:0.1:1);
A1=[2.*(1.4+y3).*0.6.*[40-
0.3.*0.5.*0.6.*15+0.3.*(1.3+y2).*15]+0.5.*(1+0.3.*0.6).*[35.*0.6-
0.5.*15+(1.4+y3).*0.6.*15]]./[4.*0.3.*(1.3+y2).*(1.4+y3).*0.6-
0.5.*0.5.*(1+0.3.*0.6).*(1+0.3.*0.6)]
B1=[0.6.*[(1+0.3.*0.6).*0.5.*y3+2.*y2.*(1.4+y3).*0.6]]./[4.*0.3.*(1.3+y2).*(1.4+y3
).*0.6-0.5.*0.5.*(1+0.3.*0.6).*(1+0.3.*0.6)]
C1=[35.*0.6-0.5.*15+(1.4+y3).*0.6.*15+0.5.*(1+0.3.*0.6).*A1]./[2.*(1.4+y3).*0.6]
D1=[0.5.*(1+0.3.*0.6).*B1+y3.*0.6]./[2.*(1.4+y3).*0.6]
A2=[0.6.*[2.*(0.9+y3+y2).*40+15.*0.3.*[2.*(0.9+y3+y2).*(1.3+y2)-y2.*y2-
0.6.*[2.*(0.9+y3+y2).*0.5+y2.*y3]]]+y2.*[0.6.*35+15.*( y3+y2)]]./[2.*0.3.*0.6.*[2.
*(0.9+y3+y2).*(1.3+y2)-y2.*y2]]
```

```
B2=[[1+0.3.*0.6].*[2.*(0.9+y3+y2).*0.5+y2.*y3]]./[2.*0.3.*(2.*(1.3+y2).*(0.9+y3+y
2)-y2.*y2)]
C2=[0.6.*[2.*(0.9+y3+y2).*35+15.*[2.*(0.9+y3+y2).*(1.4+y3)-
y3.*y3]+y3.*35]+15.*[y3.*y3-
2.*(0.9+y3+y2).*0.5]]./[2.*0.6.*[2.*(0.9+y3+y2).*(1.4+y3)-y3.*y3]]
D2=[2.*(0.9+y3+y2).*0.5+y2.*y3+0.3.*[2.*(0.9+y3+y2).*0.5+y2].*0.6]./[2.*0.6.*(2.
*(0.9+y3+y2).*(1.4+y3)-y3.*y3)]
E2=[0.3.*y2./(2.*(0.9+y3+y2))].*[A2+B2.*C2]./[1-
B2.*D2]+y3.*[C2+[D2.*(A2+B2.*C2)./(1-B2.*D2)]]./(2.*(0.9+y3+y2))
A3=[2.*(1.4+y3).*0.6.*[40-
0.3.*0.5.*0.6.*15+0.3.*(1.3+y2).*15]+0.5.*(1+0.3.*0.6).*[35.*0.6-
0.5.*15+(1.4+y3).*0.6.*15]]./[4.*0.3.*(1.3+y2).*(1.4+y3).*0.6-
0.5.*0.5.*(1+0.3.*0.6).*(1+0.3.*0.6)]
B3=[0.6.*[(1+0.3.*0.6).*0.5.*y3+2.*y2.*(1.4+y3).*0.6]]./[4.*0.3.*(1.3+y2).*(1.4+y3
).*0.6-0.5.*0.5.*(1+0.3.*0.6).*(1+0.3.*0.6)]
C3=[35.*0.6-0.5.*15+(1.4+y3).*0.6.*15+0.5.*(1+0.3.*0.6).*A3]./[2.*(1.4+y3).*0.6]
D3=[0.5.*(1+0.3.*0.6).*B3+y3.*0.6]./[2.*(1.4+y3).*0.6]
E3=15.*[-0.3.*(1.3+y2).*B3+0.5.*D3+y2+0.3.*0.5.*B3-
(1.4+y3).*D3+y3]./[2.*0.6.*[(0.9+y3+y2)-0.3.*y2.*B3-y3.*D3]]
F3=[35+0.3.*y2.*A3+y3.*C3]./[2.*[0.9+y3+y2-0.3.*y2.*B3-y3.*D3]]
P1N=A1+B1.*[0.6.*[35+0.3.*y2.*A1+y3.*C1]+15.*[y2+y3]]./[0.6.*[2.*(0.9+y3+y2)
-0.3.*y2.*B1-y3.*D1]]
P1R=(A2+B2.*C2)./(1-B2.*D2)
P1M=A3+B3.*[E3+F3]
P2M=C3+D3.*[E3+F3]
P2R=C2+[D2.*(A2+B2.*C2)]./(1-B2.*D2)
P2N=C1+D1.*[0.6.*[35+0.3.*y2.*A1+y3.*C1]+15.*[y2+y3]]./[0.6.*[2.*(0.9+y3+y2)
-0.3.*y2.*B1-y3.*D1]]
P3N=[0.6.*[35+0.3.*y2.*A1+y3.*C1]+15.*[y2+y3]]./[0.6.*[2.*(0.9+y3+y2)-
0.3.*y2.*B1-y3.*D1]]
P3R=E2+[0.6.*35+15.*( y3+y2)]./(2.*(0.9+y3+y2).*0.6)
P3M=[E3+F3]
Q1N=40-0.3.*(1.3+y2).*P1N+0.5.*P2N+y2.*P3N
Q2N=35-(1.4+y3).*P2N+0.3.*0.5.*P1N+y3.*P3N
Q3N=35-(0.9+y3+y2).*P3N+0.3.*y2.*P1N+y3.*P2N
Q1R=40-0.3.*(1.3+y2).*P1R+0.5.*P2R+y2.*P3R
Q2R=35-(1.4+y3).*P2R+0.3.*0.5.*P1R+y3.*P3R
Q3R=35-(0.9+y3+y2).*P3R+0.3.*y2.*P1R+y3.*P2R
Q1M=40-0.3.*(1.3+y2).*P1M+0.5.*P2M+y2.*P3M
Q2M=35-(1.4+y3).*P2M+0.3.*0.5.*P1M+y3.*P3M
Q3M=35-(0.9+y3+y2).*P3M+0.3.*y2.*P1M+y3.*P2M
V1M=0.6.*P3N.*Q3N+15.*(Q1N+Q2N)
V2M=0.6.*P3R.*Q3R+15.*(Q1R+Q2R)
V3M=0.6.*P3M.*Q3M+15.*(Q1M+Q2M)
surf(y2,y3,V1M);
hold on
surf(y2,y3,V2M);
surf(y2,y3,V3M);
xlabel('\gamma_2');
```

```
ylabel('\gamma_3');
zlabel(' V_M')
legend('V_M^N', 'V_M^{RS}', 'V_M^{MS}')
hold off
```

S6 Fig 7. (F) Code

```
[y2 y3]=meshgrid(0:0.1:1,0:0.1:1);
A1=[2.*(1.4+y3).*0.6.*[40-
0.3.*0.5.*0.6.*15+0.3.*(1.3+y2).*15]+0.5.*(1+0.3.*0.6).*[35.*0.6-
0.5.*15+(1.4+y3).*0.6.*15]]./[4.*0.3.*(1.3+y2).*(1.4+y3).*0.6-
0.5.*0.5.*(1+0.3.*0.6).*(1+0.3.*0.6)]
B1=[0.6.*[(1+0.3.*0.6).*0.5.*y3+2.*y2.*(1.4+y3).*0.6]]./[4.*0.3.*(1.3+y2).*(1.4+y3
).*0.6-0.5.*0.5.*(1+0.3.*0.6).*(1+0.3.*0.6)]
C1=[35.*0.6-0.5.*15+(1.4+y3).*0.6.*15+0.5.*(1+0.3.*0.6).*A1]./[2.*(1.4+y3).*0.6]
D1=[0.5.*(1+0.3.*0.6).*B1+y3.*0.6]./[2.*(1.4+y3).*0.6]
A2=[0.6.*[2.*(0.9+y3+y2).*40+15.*0.3.*[2.*(0.9+y3+y2).*(1.3+y2)-y2.*y2-
0.6.*[2.*(0.9+y3+y2).*0.5+y2.*y3]]]+y2.*[0.6.*35+15.*( y3+y2)]]./[2.*0.3.*0.6.*[2.
*(0.9+y3+y2).*(1.3+y2)-y2.*y2]]
B2=[[1+0.3.*0.6].*[2.*(0.9+y3+y2).*0.5+y2.*y3]]./[2.*0.3.*(2.*(1.3+y2).*(0.9+y3+y
2)-y2.*y2)]
C2=[0.6.*[2.*(0.9+y3+y2).*35+15.*[2.*(0.9+y3+y2).*(1.4+y3)-
y3.*y3]+y3.*35]+15.*[y3.*y3-
2.*(0.9+y3+y2).*0.5]]./[2.*0.6.*[2.*(0.9+y3+y2).*(1.4+y3)-y3.*y3]]
D2=[2.*(0.9+y3+y2).*0.5+y2.*y3+0.3.*[2.*(0.9+y3+y2).*0.5+y2].*0.6]./[2.*0.6.*(2.
*(0.9+y3+y2).*(1.4+y3)-y3.*y3)]
E2=[0.3.*y2./(2.*(0.9+y3+y2))].*[A2+B2.*C2]./[1-
B2.*D2]+y3.*[C2+[D2.*(A2+B2.*C2)./(1-B2.*D2)]]./(2.*(0.9+y3+y2))
A3=[2.*(1.4+y3).*0.6.*[40-
0.3.*0.5.*0.6.*15+0.3.*(1.3+y2).*15]+0.5.*(1+0.3.*0.6).*[35.*0.6-
0.5.*15+(1.4+y3).*0.6.*15]]./[4.*0.3.*(1.3+y2).*(1.4+y3).*0.6-
0.5.*0.5.*(1+0.3.*0.6).*(1+0.3.*0.6)]
B3=[0.6.*[(1+0.3.*0.6).*0.5.*y3+2.*y2.*(1.4+y3).*0.6]]./[4.*0.3.*(1.3+y2).*(1.4+y3
).*0.6-0.5.*0.5.*(1+0.3.*0.6).*(1+0.3.*0.6)]
C3=[35.*0.6-0.5.*15+(1.4+y3).*0.6.*15+0.5.*(1+0.3.*0.6).*A3]./[2.*(1.4+y3).*0.6]
D3=[0.5.*(1+0.3.*0.6).*B3+y3.*0.6]./[2.*(1.4+y3).*0.6]
E3=15.*[-0.3.*(1.3+y2).*B3+0.5.*D3+y2+0.3.*0.5.*B3-
(1.4+y3).*D3+y3]./[2.*0.6.*[(0.9+y3+y2)-0.3.*y2.*B3-y3.*D3]]
F3=[35+0.3.*y2.*A3+y3.*C3]./[2.*[0.9+y3+y2-0.3.*y2.*B3-y3.*D3]]
P1N=A1+B1.*[0.6.*[35+0.3.*y2.*A1+y3.*C1]+15.*[y2+y3]]./[0.6.*[2.*(0.9+y3+y2)
-0.3.*y2.*B1-y3.*D1]]
P1R=(A2+B2.*C2)./(1-B2.*D2)
P1M=A3+B3.*[E3+F3]
P2M=C3+D3.*[E3+F3]
P2R=C2+[D2.*(A2+B2.*C2)]./(1-B2.*D2)
P2N=C1+D1.*[0.6.*[35+0.3.*y2.*A1+y3.*C1]+15.*[y2+y3]]./[0.6.*[2.*(0.9+y3+y2)
-0.3.*y2.*B1-y3.*D1]]
P3N=[0.6.*[35+0.3.*y2.*A1+y3.*C1]+15.*[y2+y3]]./[0.6.*[2.*(0.9+y3+y2)-
0.3.*y2.*B1-y3.*D1]]
```

```
P3R=E2+[0.6.*35+15.*( y3+y2)]./(2.*(0.9+y3+y2).*0.6)
P3M=[E3+F3]
Q1N=40-0.3.*(1.3+y2).*P1N+0.5.*P2N+y2.*P3N
Q2N=35-(1.4+y3).*P2N+0.3.*0.5.*P1N+y3.*P3N
Q3N=35-(0.9+y3+y2).*P3N+0.3.*y2.*P1N+y3.*P2N
Q1R=40-0.3.*(1.3+y2).*P1R+0.5.*P2R+y2.*P3R
Q2R=35-(1.4+y3).*P2R+0.3.*0.5.*P1R+y3.*P3R
Q3R=35-(0.9+y3+y2).*P3R+0.3.*y2.*P1R+y3.*P2R
Q1M=40-0.3.*(1.3+y2).*P1M+0.5.*P2M+y2.*P3M
Q2M=35-(1.4+y3).*P2M+0.3.*0.5.*P1M+y3.*P3M
Q3M=35-(0.9+y3+y2).*P3M+0.3.*y2.*P1M+y3.*P2M
V1R=(P1N-15).*Q1N+0.6.*(P2N-15).*Q2N
V2R=(P1R-15).*Q1R+0.6.*(P2R-15).*Q2R
V3R=(P1M-15).*Q1M+0.6.*(P2M-15).*Q2M
V1M=0.6.*P3N.*Q3N+15.*(Q1N+Q2N)
V2M=0.6.*P3R.*Q3R+15.*(Q1R+Q2R)
V3M=0.6.*P3M.*Q3M+15.*(Q1M+Q2M)
V1=V1R+V1M
V2=V2R+V2M
V3=V3R+V3M
surf(y2,y3,V1);
hold on
surf(y2,y3,V2);
surf(y2,y3,V3);
xlabel('\gamma_2');
ylabel('\gamma_3');
zlabel('V')
legend('V^N', 'V^{RS}', 'V^{MS}')
hold off
```
